# Identification of Pipe Damage by Line-Structured Light and Regional Resonance Pairs

**DOI:** 10.3390/s25227020

**Published:** 2025-11-17

**Authors:** Mingyuan Wang, Yiqing Gu, Yuhua Wu, Jiuhong Jia, Shan-Tung Tu

**Affiliations:** Key Laboratory of Pressure Systems and Safety, Ministry of Education, East China University of Science and Technology, Shanghai 200237, China; y10230085@mail.ecust.edu.cn (M.W.); gyq@mail.ecust.edu.cn (Y.G.); y30230691@mail.ecust.edu.cn (Y.W.); sttu@ecust.edu.cn (S.-T.T.)

**Keywords:** line-structured light, baseline-free, regional resonance pairs, damage identification, vibration

## Abstract

Baseline-free methods in structural health monitoring (SHM) are crucial as they allow for real-time damage detection and assessment, eliminating the dependency on initial condition data. This study proposes a baseline-free damage detection method for pipes by combining line-structured light (LSL) with regional resonance pairs (RRPs). The 5 damage indices, combined with the RRP frequency ratio, enable Level 3 damage identification (detection, location, and quantification). The key idea is to sort the RRP frequencies to predict the damage location. A pre-established quantitative relationship is then used to estimate the damage based on the form of material volume loss. The proposed method is evaluated by 800 simulated test cases, showing an accuracy of around 90% for damage detection, location, and quantification. The method is further validated through modal data from a pipe with artificial damage, which provides a novel idea for baseline-free damage identification in pipes.

## 1. Introduction

Pipes are widely used for the long-distance transportation of oil, gas, and chemicals. These structures are vulnerable to various types of damage, such as corrosion, dents, and cracks [1,2]. Such damage can seriously affect the performance of the structure and may even lead to failure and catastrophic consequences. Therefore, early detection and assessment of damage are critical for ensuring the safety of the structure. Since the early 1970s, Structural Health Monitoring (SHM) has been extensively researched. Multi-level damage identification technology has been developed primarily for various types of structures such as beams [3], plates [4], railways [5], bridges [6], and large-scale infrastructures like space frames [7]. Traditional Non-Destructive Testing (NDT) methods usually rely on fixed inspection intervals, which have certain limitations [8]. Long inspection intervals may miss early-stage damage, allowing it to become severe by the time the next check is performed. On the other hand, very short intervals lead to frequent and costly inspections. To address these issues, SHM technology has become an effective and cost-efficient solution for assessing structural integrity [9,10]. The main goal of SHM is to detect damage in real time, accurately locate its position, assess its severity, and predict the remaining service life of the structure [11,12]. Some SHM methods focus on damage detection [13,14], while others go further to locate [15,16,17] and quantify [18] the damage. By installing sensors and continuously collecting data, SHM systems can turn a structure into a “smart structure” for automated damage diagnosis [19,20]. This approach reduces maintenance costs, prevents catastrophic failures, and improves the accuracy and efficiency of detection. As a result, SHM not only reduces the frequency of traditional inspections but also covers hard-to-reach areas, enhancing the overall safety of the structure.

Technological advancements, such as improvements in sensor devices and increases in computational power and speed, have also contributed to the development of SHM [21]. Recent studies have been based on guided wave ultrasound technology [22,23,24], while others have focused on line-structured light (LSL) techniques [25,26,27]. Vibration-based pipe condition monitoring methods are less common [28]. Some identified methods [29] include numerical studies by models and wavelet analysis to detect thin walls and cracks. Other methods [30,31] involve experimental studies on frequency shift curves for identifying local defects and numerical approaches for corrosion detection based on shifts in natural frequencies and Rayleigh’s law. These damage-identification methods are only focused on detecting and locating damage. Few attempts have been made for the Level 3 damage identification (quantification) and the Level 4 damage identification (life prediction) is still in its early stages [32]. In most methods, baseline modal signatures are not always available, which cannot be compared with the modal signatures of the tested structure in a damaged state. Most vibration-based methods rely on modal shapes for damage localization [3,21]. The acquisition of modal shapes usually relies on a large number of sensors, optimal sensor locations, and other rigorous analyses before damage detection [33]. This study aims to address these issues by developing a baseline-free method for damage detection, localization, and quantification.

There is research on measuring the rotational symmetry of N-sided polygons [34], which developed a method called continuous symmetry measurement (CSM). It could be used to quantify the symmetry of radial mode shapes of corroded pipe sections [35], but it is only applicable to polygonal cross-sections. There are also studies on symmetry detection methods for fluid flow in vortex rings [36], but they cannot be used to detect structural damage. This study is also exploring a symmetry-measurement method for pipes (circular cross-sections).

**Figure 1:**
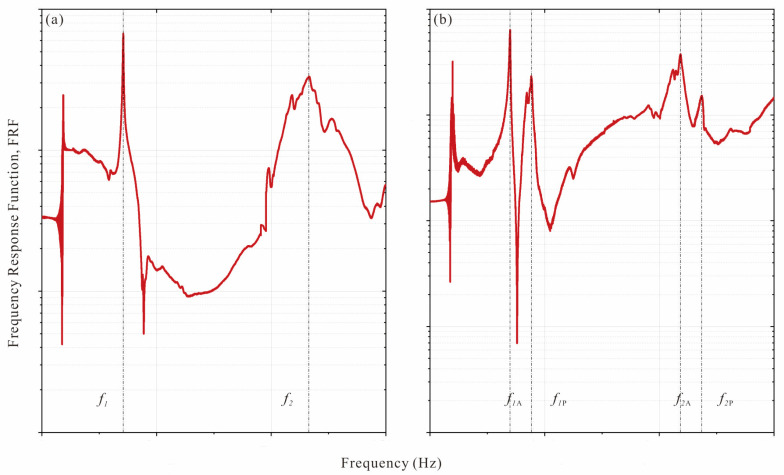
FRF examples of the pipe: (**a**) undamaged, showing a single peak for each valid vibration mode; (**b**) non-axisymmetric damage, showing a double peak for each valid vibration mode, corresponding to the active and passive RRP components (subscripts “A” and “P”, respectively).

The regional resonance pair (RRP) phenomenon is introduced, which is directly related to the proposed method. We have already implemented pipe damage detection in previous research [37], and the experimental results show that this phenomenon only occurs in axisymmetric pipes with non-axisymmetric damage. A pair of RRPs usually consists of active and passive components, which are represented by _A_ and _P_, respectively, as shown in Figure 1. Previous research results have shown that the RRP phenomenon only occurs when the pipe is damaged by non-axisymmetric damage. RRP cannot be observed in axisymmetric damage and undamaged structures, that is, *f*_1A_ = *f*_1P_ = *f*_1_ and Φ_1A_ = Φ_1P_ = Φ_1_. The appearance of RRP could prove the existence of damage.

The stability of the RRP frequency ratio can be influenced by measurement noise, excitation direction, and variations in boundary conditions. Measurement noise may interfere with the identification of frequency response peaks, leading to slight deviations in the characteristic frequencies and their ratios. Although noise is inevitable, high-frequency random disturbances can be effectively suppressed through techniques such as bandpass filtering, signal smoothing, or wavelet denoising. These methods help maintain the stability of frequency ratio rankings without altering the primary modal characteristics. The excitation direction determines the extent to which each mode is activated. As demonstrated in previous studies [37], combining the results of modes excited in different directions is essential for a comprehensive assessment. Finally, variations in boundary conditions have a direct impact on the system’s frequency distribution and represent one of the most significant factors influencing RRP ranking. While such changes typically cause only a shift in frequency values, structural damage can still lead to alterations in the RRP of the modes.

In vibration mode *i*, if the ratio of *f_i_*_A_/*f_i_*_P_ is less than 1.0, it indicates that the current pipe is damaged. Since the active RRP frequency (and mode shape) is less sensitive than the passive one, it could be used to replace the undamaged modal characteristics to achieve baseline-free damage detection. In previous studies [37], the occurrence law of the RRP phenomenon is mainly studied when it is known in advance whether the pipe is damaged. This study introduces a novel damage-identification method based on LSL and RRP, mainly solving its inverse problem. The study describes the method of RRP and LSL damage identification process and then demonstrates its application. The method is verified, and the accuracy is evaluated through 600 simulation test cases. It is also experimentally verified based on laboratory test data. The results show that the method achieves good Level 3 damage identification without the need for a baseline.

## 2. Methodology

### 2.1. Overview

**Figure 2:**
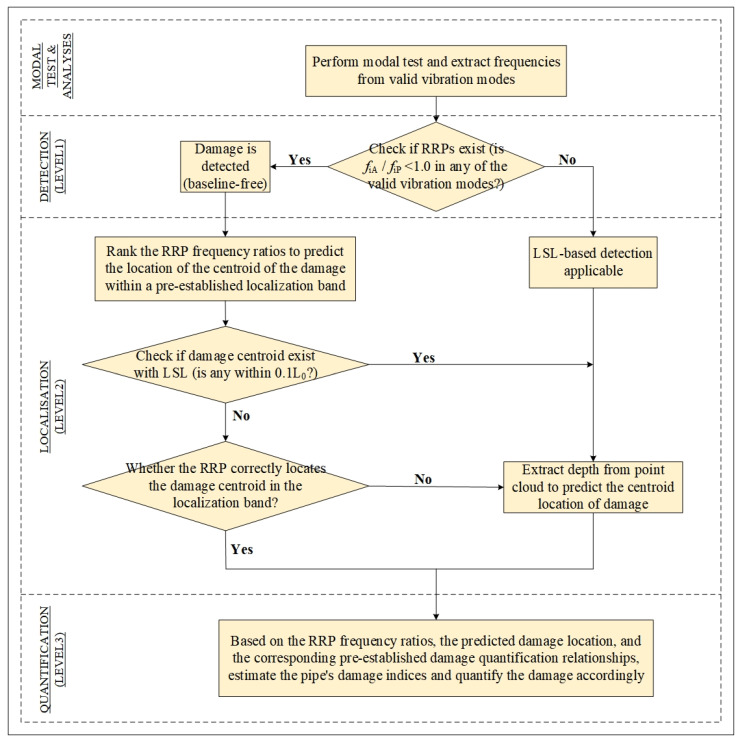
Flowchart of the proposed damage-identification method.

Level 3 damage identification aims to quantify the damage in a structure by detecting (Level 1) and locating (Level 2) it, all without relying on prior knowledge of the structure’s original undamaged condition. Essentially, this method solves the inverse problem, where damage is identified based on the current vibration data or modal characteristics of the structure. This study introduces a baseline-free method combining RRP frequency and LSL to achieve damage detection, localization, and quantification in pipes. This method could be further applied to other axisymmetric structures. The entire damage identification process is shown in Figure 2.

Damage identification begins by performing a modal test and extracting frequencies from valid vibration modes. Damage is detected if the RRP frequency ratio (*f_i_*_A_/*f_i_*_P_) is found to be less than 1.0 in any of the valid vibration modes. If damage is detected, the process moves to the next step, which is damage localization.

Once damage is detected, the RRP frequency ratios are ranked to predict the centroid location of the damage within predefined localization bands. The setting of the minimum *M_d_* value influences the localization band’s accuracy, so the LSL is used to assist in confirming the predicted damage centroid location. When the damage centroid falls within the range defined by the minimum of *M_d_*, LSL is directly used to extract depth from point cloud to precisely locate the damage. In cases where the damage centroid is outside the range of the minimum of *M_d_*, the localization band is first used to verify the centroid location. If the centroid is correctly located, the process proceeds to the quantification stage. Otherwise, depth from point cloud is employed to further refine the centroid position and the process advances to damage quantification. In the damage quantification, based on the RRP frequency ratios, the predicted location of the damage, and the corresponding pre-established damage quantitative relationships, the geometric damage index of the structure is estimated, and the damage is quantified accordingly.

The proposed hybrid RRP–LSL system establishes an integrated NDT–SHM diagnostic framework that combines vibration-based modal analysis with surface-scanning inspection to enable multi-level structural assessment. Through this cooperative configuration, the limitations of individual SHM or NDT methods are overcome, forming a complementary diagnostic scheme in which continuous modal monitoring is provided by RRP, while high-resolution surface characterization is achieved by LSL. By integrating these two approaches, both damage visualization and quantitative accuracy are enhanced, effectively bridging the gap between global monitoring and localized inspection.

### 2.2. Damage Database

**Figure 3:**
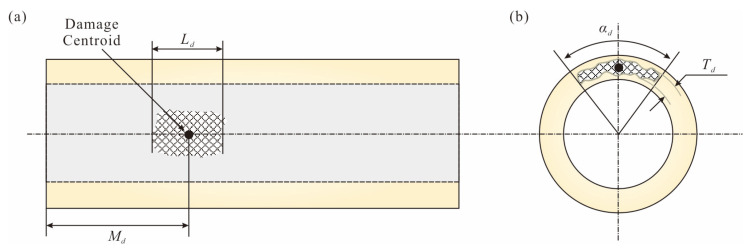
Example diagram of parameters for a damaged pipe: (**a**) damage centroid, *L_d_* and *M_d_*; (**b**) *α_d_* and *T_d_*.

To develop a database for locating damage, it is necessary to create finite element models of pipes with different damage characteristics, including length, angle, depth, and centroid position. The damage characteristics of the pipe are defined as shown in Figure 3.

**Figure 4:**
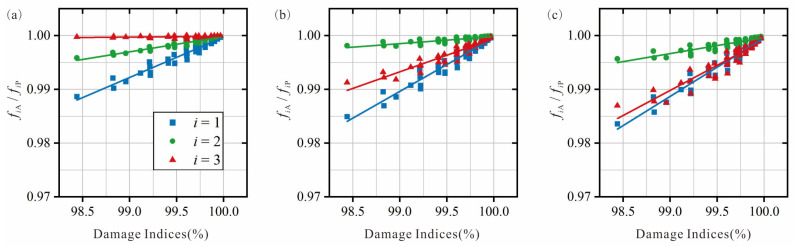
Example diagram of the relationship between the damage indices of each vibration mode *i* and the RRP frequency ratio: Damaged centroid, *M_d_* in (**a**) L_1_, (**b**) L_2_, and (**c**) L_3_.

The modal analysis results for each test case in the database are counted, the presence of RRP is checked based on the frequency results for each vibration mode *i*, and the corresponding ratio *f_i_*_A_/*f_i_*_P_ is calculated. The database contains the damage attributes, indices, and RRP frequencies for each test case. The definition of damage indices can be found in Section 2.3. Plot the damage index against the corresponding RRP frequency ratio for each damage centroid position *M_d_* (for each vibration mode *i*), as shown in Figure 4. The plots show how the damage centroid *M_d_* changes across three positions (*M_d_* = L_1_, L_2_, L_3_) for the three vibration modes (*i* = 1–3). The trend line equations in this study are referred to as quantitative relationships. Their primary function is to initialize localization bands (for damage location) and estimate damage indices (for damage quantification).

**Figure 5:**
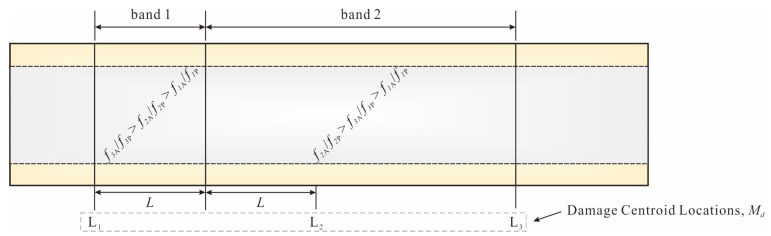
Example of two localization bands for pipe.

The pipe could be divided into multiple damage localization bands based on the sorting of the RRP frequency ratios for each test case. In Figure 4a, the order of the intercepts *b* at each vibration mode *i* is *b*_3_ > *b*_2_ > *b*_1_. Therefore, when L_1_ ≤ *M_d_* ≤ L_2_, the order is *f*_3A_/*f*_3P_ > *f*_2A_/*f*_2P_ > *f*_1A_/*f*_1P_. Similarly, as shown in Figure 4b,c, the order of the RRP frequency ratios at the L_2_ and L_3_ damage centroids is *f*_2A_/*f*_2P_ > *f*_3A_/*f*_3P_ > *f*_1A_/*f*_1P_. The number of frequency ratios in the order corresponds to the number of vibration modes *i* studied. The range of centroid locations is used to define the bandwidth of the damage localization band. As shown in Figure 5, the pipe is divided into two bands, with the bandwidth L_1_ ≤ *M_d_* ≤ (average of L_2_ and L_3_) corresponding to band 1 and (average of L_2_ and L_3_) < *M_d_* ≤ L_3_ corresponding to band 2.

The RRP ratio ranking offers a qualitative physical explanation for this rule through modal analysis [37]. The underlying physical principle of this rule arises from the modal splitting phenomenon caused by damage. Damage disrupts the symmetry of the pipe structure, introducing anisotropy in the pipe’s stiffness at the affected location. This disruption leads to the splitting of an originally degenerate mode into two independent modes with similar but distinct frequencies. Our results indicate that the magnitude of this split is closely related to the degree of anisotropy introduced by the damage, which depends on the relative magnitude of the strain energy at the damage site in that mode. The observed RRP ratio ranking (e.g., *f*_3A_/*f*_3P_ > *f*_2A_/*f_2_*_P_ > *f*_1A_/*f*_1P_) could be interpreted as follows: at the damaged region, the strain energy density of the third mode is the highest, and the symmetry disruption of the third mode is most severe, resulting in the largest splitting amplitude.

### 2.3. Damage Indices

Five damage indices are developed in this study to characterize the damage disturbance of RRP to pipes. Given that RRP exhibits characteristics of symmetrical disturbances, in addition to traditional damage indices, this study also develops damage indices driven by symmetry. When these indices are linked to their corresponding RRP frequency ratios, they generate relationships that allow for damage quantification. The first group of traditional damage indices are given in:

(1) $$V=\frac{V_{0}-V_{d}}{V_{0}}(\%)$$

(2) $$A=\frac{A_{0}-A_{d}}{A_{0}}(\%)$$

(3) $$S=\frac{S_{0}-S_{d}}{S_{0}}(\%)$$

where *V*_0_, *A*_0_, and *S*_0_ represent the volume, cross-sectional area, and surface area of the undamaged pipe, while *V_d_*, *A_d_*, and *S_d_* represent the corresponding damage amounts in the damaged pipe.

**Figure 6:**
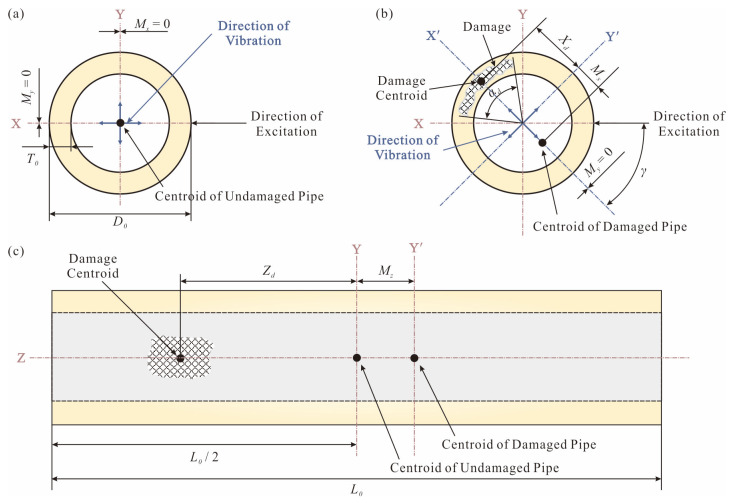
Geometric characteristics of the pipe: (**a**) undamaged cross-section, (**b**) damaged radial cross-section, and (**c**) damaged axial cross-section.

The second group consists of symmetry-driven damage indices. *δ_x_* and *δ_z_* are related to the radial and axial symmetry of the pipe, respectively. As shown in Figure 6, an undamaged pipe has an outer diameter *D*_0_ and a wall thickness *T*_0_. When an undamaged pipe is excited, it will vibrate in any orthogonal directions, here labeled X-axis and Y-axis. The centroid of the pipe along the two vibration axes coincides with its geometric center, i.e., *M_x_* = *M_y_* = 0. Therefore, in each vibration mode *i*, the frequency and mode are equal in each direction, i.e., *f_i_*(X) = *f_i_*(Y) and Φ*_i_*(X) = Φ*_i_*(Y).

Damage interrupts the axial symmetry of the pipe over a certain length. When a damaged pipe is excited, a line passing through the damaged centroid and its geometric center is defined as the main vibration axis X’. Complementary vibration Y’ occurs in the direction perpendicular to X’, as shown in Figure 6b. The damage of the pipe causes the modal characteristics obtained from the two directions of the radial section to be different, i.e., *f_i_*(X) ≠ *f_i_*(Y) and Φ*_i_*(X) ≠ Φ*_i_*(Y). The centroid in the Y’ direction is obviously equal to zero on the main vibration axis X’ of the pipe, so *M_y_* = 0. However, the centroid in the X’ direction is not equal to zero on the complementary vibration axis Y’ of the pipe, and its offset distance is *M_x_* defined by Equation (4):

(4) $$M_{x}=-\frac{A_{d}\times X_{d}\times 2\sin(\alpha_{d}/2)/\alpha_{d}}{A_{0}-A_{d}}$$

where *X_d_* is the distance along the X’ axis from the damage centroid to the geometric center of the undamaged pipe, and *α_d_* is the angle between the damage and the geometric center of the undamaged pipe. *A*_0_ is the cross-sectional area of the undamaged pipe. When the radial cross-section of the pipe is reduced to a semicircular shape, *M_x_* reaches its maximum value *M_x_*_(max)_ and it is defined by Equation (5):

(5) $$M_{x(\max)}=\frac{D_{0}-T_{0}}{\pi }$$

The axial symmetry of the undamaged pipe has not changed, so *M_z_* = 0. The axial symmetry *M_z_* of the damaged pipe is calculated by Equation (6):

(6) $$M_{z}=-\frac{V_{d}\times Z_{d}}{V_{0}-V_{d}}$$

where *V_d_* represents the volume of the damage, *Z_d_* is the distance from the damage centroid to the geometric center of the undamaged pipe along the Z-axis, and *V*_0_ is the volume of the undamaged pipe. When half of the pipe section is completely lost, *M_z_* reaches its maximum. Therefore, the definition of *M_z_*_(max)_ is as shown in Equation (7):

(7) $$M_{z(\max)}=\frac{L_{0}}{4}$$

Based on *M_x_* and *M_x_*_(max)_, the degree of pipe radial symmetry *δ_x_* by Equation (8) and its range goes from 0 (minimal radial symmetry) to 100 (maximum radial symmetry).

(8) $$\delta_{x}=\frac{M_{x(\max)}-M_{x}}{M_{x(\max)}}(\%)$$

Similarly, based on *M_z_* and *M_z_*_(max)_, the axial symmetry *δ_z_* is defined as follows:

(9) $$\delta_{z}=\frac{M_{z(\max)}-M_{z}}{M_{z(\max)}}(\%)$$

### 2.4. Damage Identification Process

**Figure 7:**
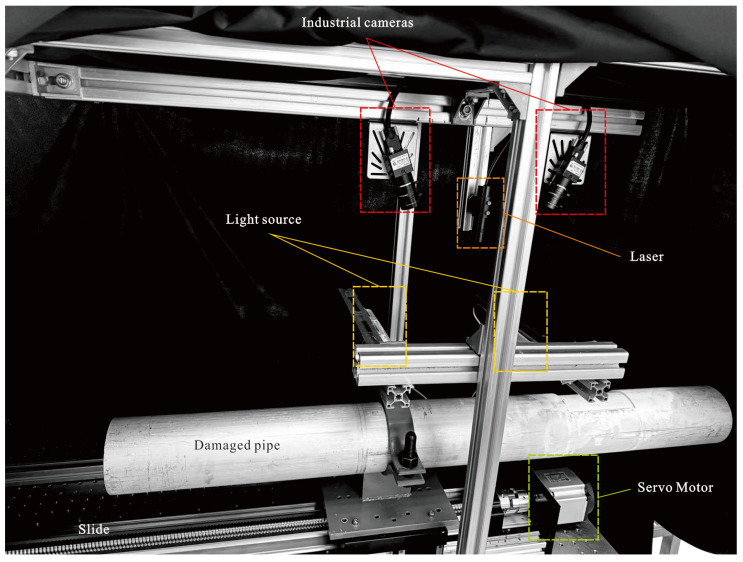
Composition of the line-structured light system.

For damage detection, if the RRP frequency ratio is observed to be equal to 1.00, it means that the pipe is not damaged or symmetrically damaged, while the opposite is true for damaged pipes. For damage centroid location, it is necessary to implement the sorting based on RRP frequency ratio and the assistance of LSL system. The damage centroid location could be predicted by reverse associating the sorted frequency ratios with predefined localization bands. It is important to note that, if the RRP frequency ratio order in a specific case does not match any of the predefined localization bands, the LSL system should be used to assist in locating the damage centroid, as shown in Figure 7, using the LSL system. The model reconstruction work based on the point cloud collected during the scanning is carried out with the OpenCV C++ library. The entire LSL system consists of the following equipment: a line laser, two 4W LED lights, two Hikvision industrial cameras (model MV-CU013-80UM) with MVL-HF0824M-10MP lenses. This setup gives the scanning field of view a length of 264.9 mm and a width of 211.9 mm, with a heavy-duty lead screw slide driven by a servo motor that has an effective stroke of 1100 mm.

For example, if the RRP frequency ratio order is *f*_3A_/*f*_3P_ > *f_2_*_A_/*f*_2P_ > *f*_1A_/*f*_1P_, the damage centroid will be located in band 1, as shown in Figure 5. However, if the order is *f*_1A_/*f*_1P_ > *f*_2A_/*f*_2P_ > *f*_3A_/*f*_3P_, the damage cannot be localized within any of the predefined localization bands. The RRP frequency ratios and the corresponding quantitative relationship (for each localization band) obtained through testing could be used to estimate the corresponding damage index, thereby achieving damage quantification. For example, if the predicted damage centroid is within band 1, the corresponding damage index could be calculated by the quantitative relationship in Figure 4a. Then, damaged pipes could be quantified by Equations (1)–(9). It is important to note that, if the predicted damage range is within band 2, either of the 2 quantitative relationships shown in Figure 4b,c could be applied. In this case, the sum (average) of all relationships (for each vibration mode) within the localization band could be used for the damage quantification process, leading to more accurate prediction results. For further details, refer to Section 3.3 on evaluation of damage identification accuracy.

## 3. Application of Proposed Method

### 3.1. Database Development

In ANSYS Workbench 2024, the undamaged pipes listed in Table 1 are modeled and subjected to modal analysis. The boundary conditions of the pipes are set to clamp–clamp, and the size of the finite element tetrahedral mesh is 5 mm. As shown in Table 2, the geometric characteristics and RRP modal frequency values of the undamaged pipe are obtained based on Equations (5) and (7) and the dimensions in Table 1.

**Figure 8:**
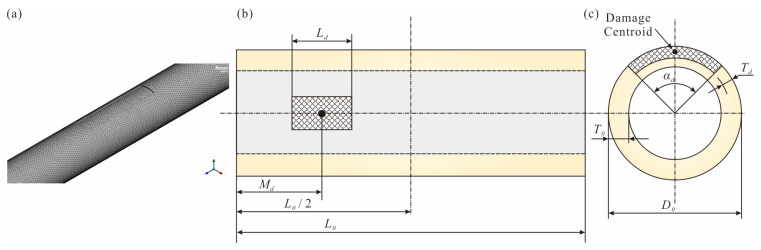
Representation of damage parameters: (**a**) finite element mesh; (**b**) axial cross-section; and (**c**) radial cross-section.

Figure 8a shows the mesh model of the damaged pipe, while Figure 8b,c show the damaged dimensions of the pipe in the axial and radial directions. Table 3 lists the range of values for each parameter. In the orthogonal experiment, nine different positions for the damage centroid *M_d_* are set, four values for the radial angle *α_d_* and thickness *T_d_*, and three lengths *L_d_* for the damage. Therefore, a total of 432 damage scenarios are included in the numerical database (see Table S1). Specifically, *M_d_* and *L_d_* consider the damage variation along the pipe, *T_d_* considers the variation in damage thickness on the pipe cross-section, and *α_d_* considers the angle of damage within the cross-section. Based on the Python (version 3.12) script in ANSYS, finite element modeling, modal analysis, modal result extraction, and database storage are completed in batches. It should be noted that, since the pipe is modeled with clamped boundary conditions, *M_d_* does not need to be adjusted to be greater than 0.5*L*_0_, as the results will always be symmetric around the central axis of the pipe. In addition, since different damage dimensions are considered, *M_d_* cannot be set to less than 0.1*L*_0_. Therefore, if the centroid of the actual damage is located at the position of *M_d_* < 0.1*L*_0_, it is recommended to use the LSL system to assist in damage identification.

By the damage dimensions provided in Table 3 and the pipe properties in Table 1, the damage indices are calculated for each damage scenario, and the first three pairs of RRP frequencies are determined. These are stored in the database (see Table S1) to establish quantitative relationships and localization bands. The use of an automated script to randomly select non-repeating parameters (*M_d_*, *α_d_*, *L_d_*, and *T_d_*) to define four groups of test cases (see Table S2), with each group containing 200 damage scenarios. Table 4 lists the maximum and minimum values chosen for each random variable in each test case. The script is setup to select the variables with a precision of 1% (i.e., 2 decimal places) within their respective boundaries, providing a wide range of options for selection.

### 3.2. Establishment of Localization Bands

**Figure 9:**
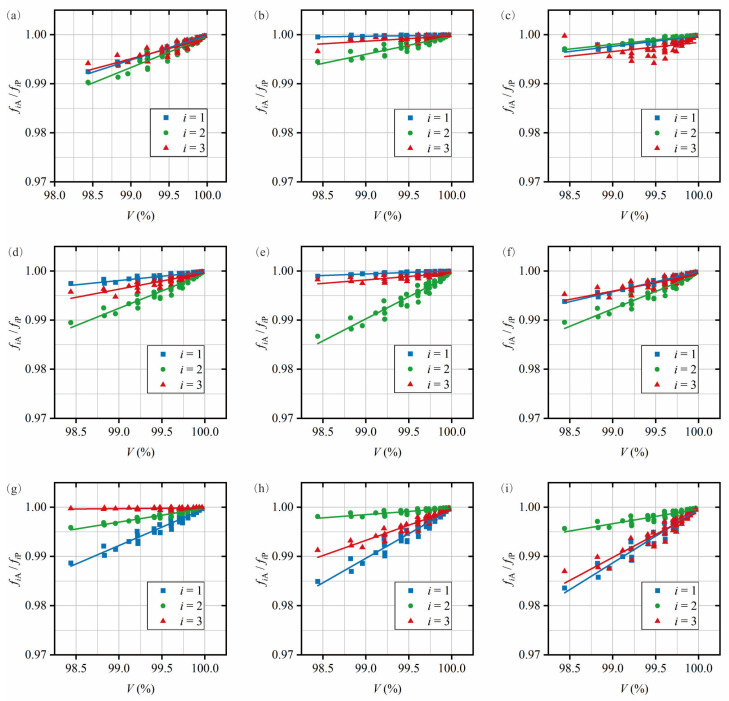
Quantification relationship of the *V* for each vibration mode *i* used to estimate the damage centroid located at (**a**) *M_d_* = 0.1*L*_0_, (**b**) *M_d_* = 0.15*L*_0_, (**c**) *M_d_* = 0.2*L*_0_, (**d**) *M_d_* = 0.25*L*_0_, (**e**) *M_d_* = 0.3*L*_0_, (**f**) *M_d_* = 0.35*L*_0_, (**g**) *M_d_* = 0.4*L*_0_, (**h**) *M_d_* = 0.45*L*_0_, and (**i**) *M_d_* = 0.5*L*_0_.

For each damage scenario in the database, the damage index is associated with the corresponding RRP frequency ratio (*f_i_*_A_/*f_i_*_P_), and a least squares linear regression trend line expression is provided for each damage centroid location, *M_d_*. In order to achieve a more direct damage quantification, the volume damage index is selected. Figure 9 shows the relationship between the volume damage index *V* and the RRP frequency ratio of different vibration modes *i*, and the respective curves are plotted according to the different damage centroid locations. Table 5 details the definition of each localization band.

Table 6 includes the trend line equations (quantification relationships) for each graph. The pipe is divided into five localization bands (with initial bandwidths). During the construction of the localization bands, the initial RRP frequency sorting order in Figure 9b is *f*_1A_/*f*_1P_ > *f*_3A_/*f*_3P_ > *f*_2A_/*f*_2P_, as the intercept sizes for the vibration modes followed the order *b*_1_ > *b*_3_ > *b*_2_. In Figure 9f, the initial sorting order is *f*_3A_/*f*_3P_ > *f*_1A_/*f*_1P_ > *f*_2A_/*f*_2P_. The localization bands are continuously adjusted based on the first group of test cases to achieve higher accuracy (see Section 3.3). As a result, the RRP frequency ratio sorting orders in Figure 9b,f are swapped. For the first group of test cases, when the initial RRP frequency sorting order is *f*_1A_/*f*_1P_ > *f*_2A_/*f*_2P_ > *f*_3A_/*f*_3P_, localizing it in band 2 significantly improved localization accuracy. This shows the importance of adjusting the initial bandwidth RRP frequency ratio sorting.

**Figure 10:**
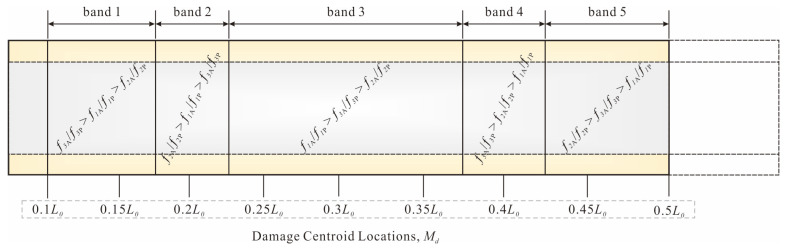
Damage localization bands and corresponding RRP frequency ratio ranking.

Many incorrectly located cases in bands 1 and 3 gathered at the boundaries of the bands. Therefore, when cases are located in band 1 or band 3, the LSL system should be used to assist in detecting the damage centroid. The final damage localization bands and their corresponding RRP frequency ratio sorting are included in Table 5 and are graphically displayed in Figure 10.

**Figure 11:**
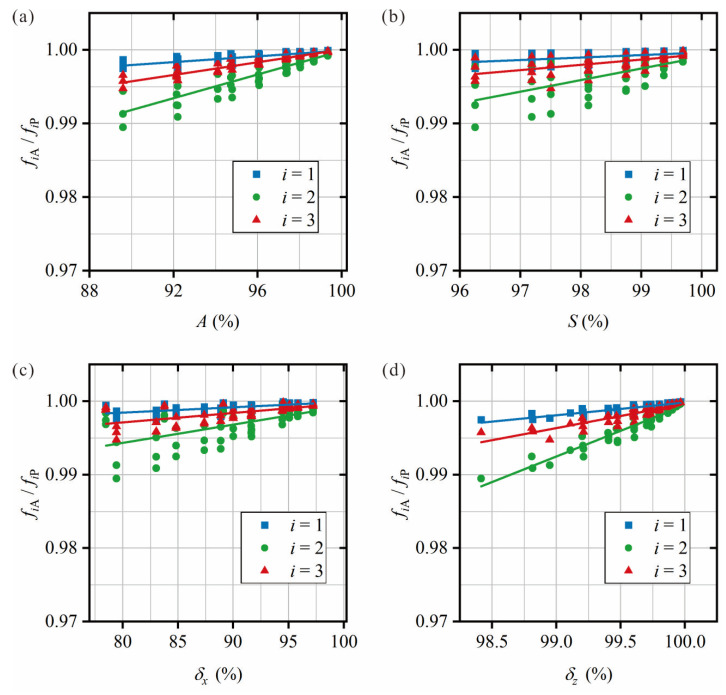
Relationship between damage indices and RRP frequency ratio for each vibration mode *i*, with the damage centroid located at *M_d_* = 0.25*L*_0_: (**a**) radial cross-sectional area, A; (**b**) surface area, s; (**c**) radial symmetry, *δ_x_*; (**d**) axial symmetry, *δ_z_*.

As shown in Figure 11, the relationships between other indices in the database (*S*, *A*, *δ_x_*, and *δ_z_*) and the RRP frequency ratio for each damage scenario are presented. The linear regression fit for surface area *S* and radial symmetry *δ_x_* is not as strong as the fit for cross-sectional area *A* and axial symmetry *δ_z_* with the RRP frequency ratio, as shown in Figure 11a,c for the damage at *M_d_* = 0.25*L*_0_. The plot in Figure 11d for axial symmetry *δ_z_* with the RRP frequency ratio shows the most consistent relationship. This means that the axial symmetry of a given set of RRP frequencies could be estimated, allowing an effective estimation of the pipe’s *M_z_* shift due to damage, as defined in Equation (9). However, for clearer damage quantification, the damage index used in this study is volume, *V*.

### 3.3. Evaluation of Damage Identification Accuracy

A total of 800 cases (see Table S2) were all tested by the localization bands and quantitative relationships established in Section 3.2. The accuracy of Level 1 identification, *P*_1_, is determined by Equation (10):

(10) $$P_{1}=\frac{N_{1}}{N}(\%)$$

where *N*_1_ is the number of cases that successfully achieve Level 1 damage identification, and *N* is the total number of three test sets excluding that used for sensitivity analysis. The Level 1 damage identification accuracy for the 600 test cases in the remaining three groups reached 100%, which means that the damage is successfully detected in all 600 damage test cases. This first group is not included in the calculations for the overall accuracy evaluation, because it is used for sensitivity analysis to improve the ability of the localization.

The accuracy in Level 2 identification is represented by *P*_2_, which is determined by Equation (11):

(11) $$P_{2}=\frac{N_{2}}{N_{1}}(\%)$$

where *N*_2_ is the number of cases correctly located within the localization band. The results of 200 numerical cases in each group (four groups in total) are listed in Table 7. The first group does not participate in the calculation of the overall accuracy, and the overall level 2 damage identification accuracy is 97.17%. The results for band 4 had the worst localization, while band 2 showed the best localization. This due to the fact that their bandwidth is narrower than that of the other localization bands (see Table 5) and influenced by adjustments made.

**Figure 12:**
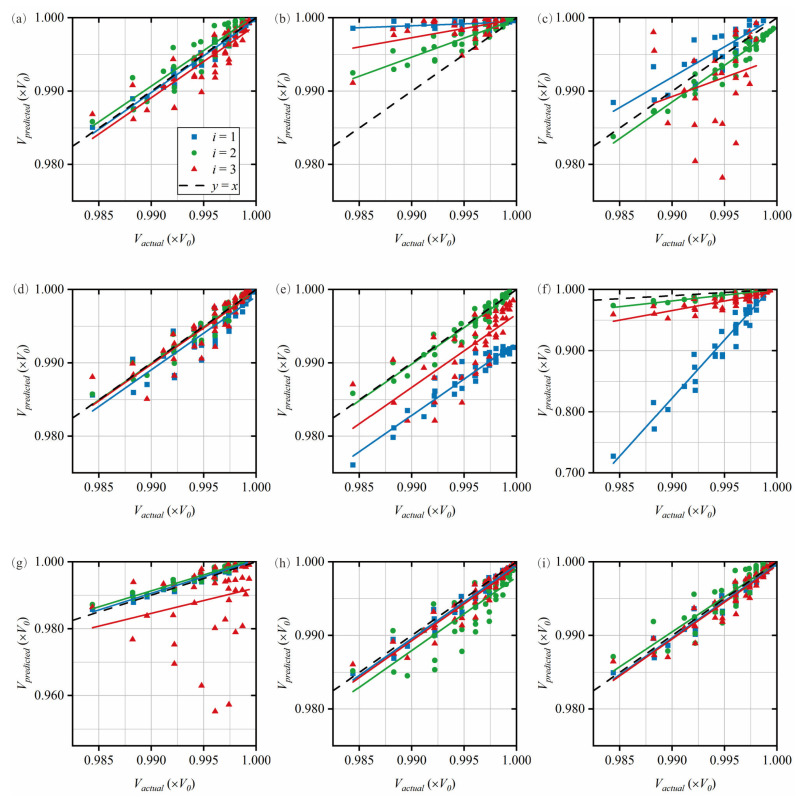
Comparison of the actual volume and predicted volume for each localization band of the damaged pipe. (**a**) band 1, *M_d_* = 0.10*L*_0_, (**b**) band 1, *M_d_* = 0.15*L*_0_, (**c**) band 2, *M_d_* = 0.20*L*_0_, (**d**) band 3, *M_d_* = 0.25*L*_0_, (**e**) band 3, *M_d_* = 0.30*L*_0_, (**f**) band 3, *M_d_* = 0.35*L*_0_, (**g**) band 4, *M_d_* = 0.40*L*_0_, (**h**) band 5, *M_d_* = 0.45*L*_0_, and (**i**) band 5, *M_d_* = 0.50*L*_0_.

The first group of 200 simulation test cases is used to adjust the damage localization band, i.e., to analyze its sensitivity to damage. Taking the first group as an example, 7 cases did not successfully achieve secondary damage identification, so only 193 cases are included in the third-level damage identification. As shown in Figure 12, the predicted value and actual value of the pipe volume at different locations of the damage centroid are given, and a 45° line is given for easy observation. These results are obtained based on the quantification relationship given in Table 6.

**Figure 13:**
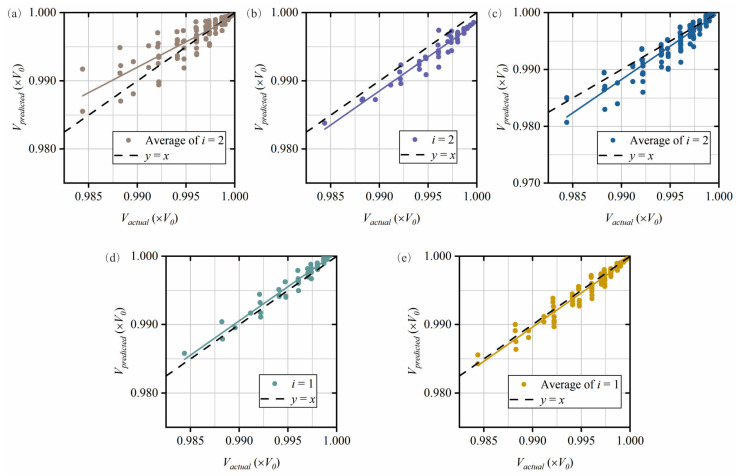
Best quantification relationship for Level 3 damage identification at (**a**) band 1, (**b**) band 2, (**c**) band 3, (**d**) band 4, and (**e**) band 5.

In the first three localization bands, we observe that the relationship of the vibration mode 2 plots to the 45° line is closer than that of the vibration mode 1 plots. In the last two bands, the situation is exactly opposite. Therefore, the first three bands are quantified using vibration mode 1, while the last two are quantified using vibration mode 2. In localization bands having multiple ratios, the total sum of all ratios within the band is used. The final plots and set of ratios are considered as the best quantitative estimate for each localization band, as shown in Figure 13 and Table 8.

The error of the proposed method in simulated test cases is evaluated based on Equation (12) to predict the accuracy of subsequent Level 3 identification.

(12) $$\varepsilon_{j}=\frac{{\left|V_{actual}-V_{predicted}\right|}_{j}}{Range\;of\;V_{actual}}(\%)$$

where *j* represents the case number, and *ε* represents the error magnitude. The range calculation method of the actual pipe damage volume *V_actual_* follows|max(*V_actual_*) − min(*V_actual_*)|. Based on the previous calculation error, the accuracy of the Level 3 identification method could be obtained by Equation (13):

(13) $$P_{3}=\frac{\sum_{j=1}^{N_{2}}\left(1-\varepsilon_{j}\right)}{N_{2}}(\%)$$

The overall Level 3 accuracy is obtained from the results of three sets of 200 numerical cases, with *P*_3_ = 86.55%. Table 9 lists the detailed results for each set of 100 cases in each localization band. As shown in Figure 13, the damage quantification accuracy within band 2 is lower than in the other bands. This is because, compared to the other localization bands, the best quantification relationship for band 2 in Figure 13b shows a weaker correlation with the actual damage amount. To further improve the quantization accuracy of Band 2, higher-order modal results need to be incorporated. However, this typically leads to a significant increase in costs. Therefore, it is essential that LSL technology be introduced into the fusion framework shown in Figure 2 to support the quantization of these localization bands.

## 4. Experimental Verification and Discussion

### 4.1. Test Specimen

**Figure 14:**
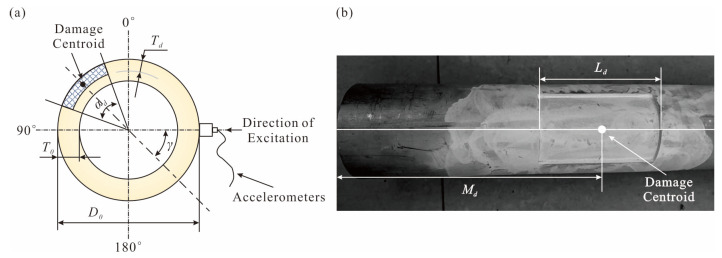
Experimental test pipe: (**a**) radial cross-sectional damage size and excitation details; (**b**) manually induced damage and its axial dimensions.

The proposed method is verified on Pipe 1 (an undamaged pipe) and Pipe 6 (a damaged pipe) based on previous research [37]. Pipe 1 is only used to compare the RRP with Pipe 6. This method has no baseline and is not required in the entire process of damage identification. The damage parameters and test conditions of the two are shown in Table 10 and Figure 14.

### 4.2. Experimental Setup and Procedure

**Figure 15:**
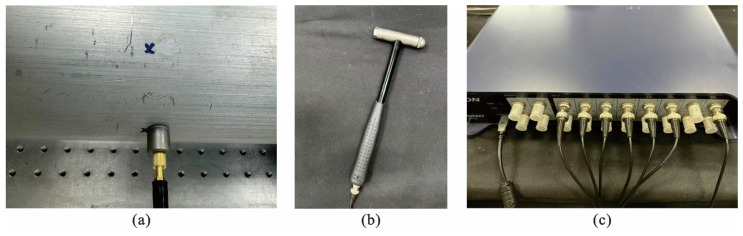
Data acquisition system: (**a**) accelerometer, (**b**) modal hammer, and (**c**) data acquisition card.

**Figure 16:**
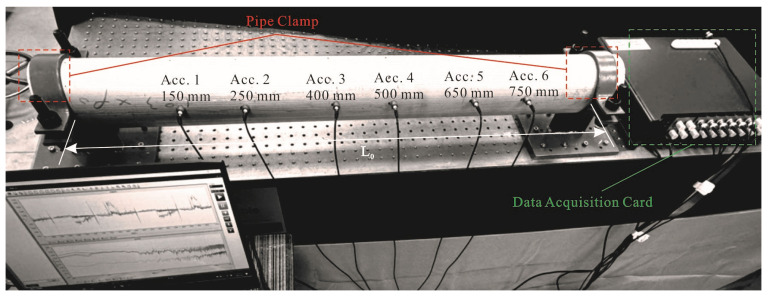
Experimental setup, showing the location of the accelerometers along the undamaged pipe, with the pipe under clamped–clamped boundary conditions.

Six single-axis accelerometers and a modal hammer consist the data acquisition system, as shown in Figure 15a,b and these accelerometers’ locations are shown in Figure 16. As shown in Figure 15c, the other end of the accelerometer and the modal hammer are connected to the data acquisition card. Each sample is fixed with two pipe clamps to simulate the clamping boundary condition, as shown in Figure 16. Eight excitations are provided along the radial section of the pipe with the gama angle (from 270° to 90°) formed by the damage centroid, as shown in Figure 14a. Previous studies [37] have explained the FRF and other data results in detail.

### 4.3. Experimental Damage Identification Results

**Figure 17:**
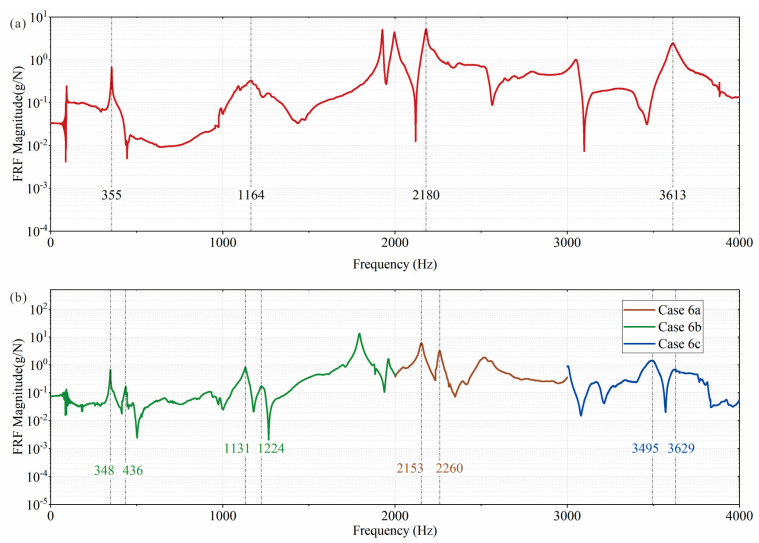
Measured frequency response function: (**a**) undamaged pipe; (**b**) damaged pipe.

As shown in Figure 17a, only one peak is observed for the undamaged pipe. For the damaged pipe, after multiple excitations, the vibration mode of the combined pipe shows two peaks (Figure 17b), corresponding to test cases 6a, 6b, and 6c when the excitation directions *γ* are 0°, 45°, and 90°, respectively. Previous studies have shown the importance of applying excitation multiple times and in at least two radial directions (e.g., 45° apart). Table 11 lists the RRP ratios (*f_i_*_A_/*f_i_*_P_) and their active and passive RRP components for each vibration mode *i* for Pipe 1 (undamaged) and Pipe 6 (damaged).

**Figure 18:**
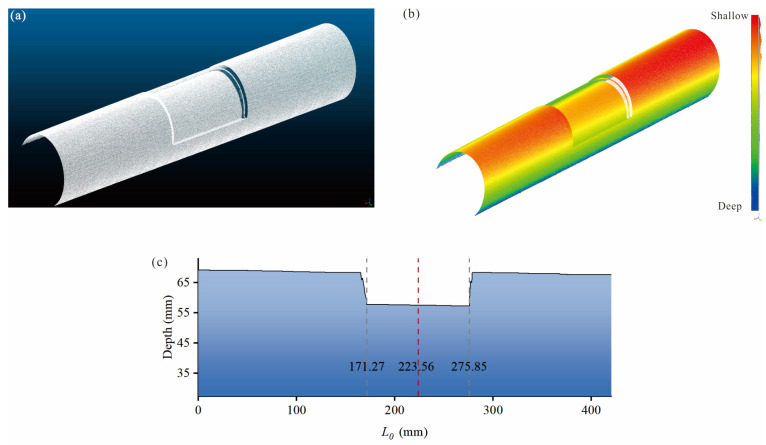
Experimental results of line-structured light scanning: (**a**) point cloud; (**b**) point cloud with depth; (**c**) depth from point cloud of the axial cross-section.

Following Table 11, it is obvious that the RRP frequency ratio of the damaged pipe is less than 1.00, while that of the undamaged pipe is equal to 1.00. The order of the RRP frequency ratio of the damaged pipe is used for positioning, i.e., *f*_3A_/*f*_3P_ > *f*_2A_/*f*_2P_ > *f*_1A_/*f*_1P_. Based on the damage localization bands preset in Table 5, it could be seen that the damage centroid should be located in the band 4, i.e., 0.375*L*_0_ < *M_d_* ≤ 0.425*L*_0_. However, the actual position of *M_d_* given in Table 10 is 0.25*L*_0_ (225 mm), which should fall within the band 3. This indicates the need to use the LSL method to assist with localization. Based on the LSL damage identification system shown in Figure 7, defect data for Table 11 damaged pipe is collected. Using CloudCompare software, the damaged pipe is represented as point cloud, as shown in Figure 18a,b. As seen in the depth from point cloud for the axial cross-section in Figure 18c, the damage centroid is located at 223.56 mm. The relative error compared to the actual position in Table 10 is 0.64%, indicating that when the relative error of *M_d_* from the boundary of the 5 localization bands in Figure 10 exceeds 0.64%, the LSL method could be used to localize *M_d_*.

According to Table 8, the best quantitative relationship for the third damage band is “*y* = 0.00658*x* + 0.3414”, which calculates the material loss volume based on vibration mode 2. Since *y* = *f*_2A_/*f*_2P_ = 0.924 listed in Table 11, the predicted result shows that the damaged pipe volume is 88.54% of the undamaged pipe volume *V*_0_,, while the actual volume of the damaged pipe is 97.81% of *V*_0_, with a relative error of 9.27% and an accuracy of 90.73%. This result may also be limited by the extrapolation ability of the relationship, i.e., the damage depth of the pipe exceeds the limit of *T_d_* in Table 3 of the preset damage localization band.

## 5. Conclusions

In this study, a novel and effective baseline-free method for damage identification in tubular structures was developed and successfully validated. The proposed method was applied to perform Level 3 damage detection on pipes with clamped–clamped boundary conditions. Compared with traditional inspection techniques, the RRP method effectively eliminates the reliance on baseline (non-damaged) reference data, thereby improving inspection efficiency and reducing costs. However, it still exhibits a certain dependence on ANSYS simulation data. Future research will aim to further reduce this dependence to enhance the method’s applicability. Moreover, it is necessary to investigate its performance under other pipe boundary conditions. The centrosymmetry of the pipe under clamped–clamped boundary conditions is known to introduce localization ambiguities. For example, a specific frequency ratio ordering (*f*_3A_/*f*_3P_ > *f*_1A_/*f*_1P_ > *f*_2A_/*f*_2P_) is observed in the region (0.1–0.175)*L*_0_. However, due to this symmetry, identical modal behavior is exhibited by damage located in the expected opposite region, (0.825–0.9)*L*_0_. As a result, damaged localization zones, constructed using the RRP method, are often rendered imprecisely or even inaccurately. By strategically combining the RRP method for rapid preliminary assessment with LSL scanning for final verification, this study establishes a comprehensive framework capable of systematically detecting, locating, and quantifying structural damage. This framework effectively addresses the problem of RRP localization ambiguity. This integrated approach significantly enhances the efficiency and robustness of pipe integrity management. The primary contributions and findings of this research can be summarized as follows:

1. A novel baseline-free damage-identification method is developed. This method successfully combines RRP with the LSL method to systematically detect, locate, and quantify damage in tubular structures.
2. The key relationship between RRP and damage is established based on the proposed damage indices. The study demonstrates that the presence of RRP is a distinct characteristic of damage, and the damage location could be determined by sorting the RRP frequency ratios.
3. The reliability of the method is validated through large-scale simulations. Across 800 test cases, the proposed method achieved a 100% success rate in damage detection, a 97.17% accuracy rate in locating the damage centroid, and an 86.55% accuracy rate for damage quantification.
4. The effectiveness of the method is confirmed by experimental verification. The results from artificially pre-damaged pipes show the method successfully detected, located, and quantified the damage, achieving a quantification accuracy of 90.73%.
5. The practical advantages of the combined method are demonstrated. By using RRP to guide the LSL scan, the approach enables rapid damage localization, significantly improving the efficiency and robustness of pipe damage identification.

## Figures and Tables

**Table 1:** Properties of pipe specimens [37].

Material	Elasticity Modulus (Mpa)	Density (kg m^−3^)	Poisso’s Ratio -	Effective Length (mm)	Outside Diameter (mm)	Wall Thickness (mm)	Unit Mass (kg m^−1^)
AL 6063 T5	68,900	2700	0.33	900	103	6.5	5.32

**Table 2:** Geometric characteristics and modal frequencies of the undamaged pipe.

*A*_0_ (mm^2^)	*S*_0_ (mm^2^)	*V*_0_ (mm^3^)	*M_x(max)_* (mm)	*M_z(max)_* (mm)	*f*_1__A_ = *f*_1P_ (Hz)	*f*_2__A_ = *f*_2P_ (Hz)	*f*_3A_ = *f*_3P_ (Hz)
1970.56	291,225.64	1,773,507.59	30.72	225	542.63	1318.60	2283.10

**Table 3:** Damage parameters of the database.

*M_d_* (mm)	*α_d_* (°)	*T_d_* (mm)	*L_d_* (mm)
90	22.5	0.65	45
135	45	1.30	90
180	67.5	1.95	135
225	90	2.60	-
270	-	-	-
315	-	-	-
360	-	-	-
405	-	-	-
450	-	-	-

**Table 4:** Boundary constraints for the selection of random damage parameters.

Boundary	*M_d_* [mm (×*L*_0_)]	*α_d_* (°)	*T_d_* [mm (×*T*_0_)]	*L_d_* [mm (×*L*_0_)]
Min	90 (0.10)	22.5	0.65 (0.10)	45 (0.05)
Max	450 (0.50)	90	2.60 (0.40)	135 (0.15)

**Table 5:** Bandwidth of the localization bands and corresponding labels.

Localization Band	Localization Bandwidth	RRP Frequency Ratio Ranking	Quantification Graph
1	0.100*L*_0_ ≤ *M_d_* ≤ 0.175*L*_0_	*f*_3A_/*f*_3P_ > *f*_1A_/*f*_1P_ > *f*_2A_/*f*_2P_	Figure 9a
			Figure 9b
2	0.175*L*_0_ < *M_d_* ≤ 0.225*L*_0_	*f*_2A_/*f*_2P_ > *f*_1A_/*f*_1P_ > *f*_3A_/*f*_3P_	Figure 9c
3	0.225*L*_0_ < *M_d_* ≤ 0.375*L*_0_	*f*_1A_/*f*_1P_ > *f*_3A_/*f*_3P_ > *f*_2A_/*f*_2P_	Figure 9d
			Figure 9e
			Figure 9f
4	0.375*L*_0_ < *M_d_* ≤ 0.425*L*_0_	*f*_3A_/*f*_3P_ > *f*_2A_/*f*_2P_ > *f*_1A_/*f*_1P_	Figure 9g
5	0.425*L*_0_ < *M_d_* ≤ 0.500*L*_0_	*f*_2A_/*f*_2P_ > *f*_3A_/*f*_3P_ > *f*_1A_/*f*_1P_	Figure 9h
			Figure 9i

**Table 6:** Quantification relationship of the localization bands in three vibration modes.

Localization Band	RRP Frequency Ratio Ranking	Quantification Relation, Vibration Mode, *i* = 1	Quantification Relation, Vibration Mode, *i* = 2	Quantification Relation, Vibration Mode, *i* = 3
1	*f*_3A_/*f*_3P_ > *f*_1A_/*f*_1P_ > *f*_2A_/*f*_2P_	*y* = 0.005*x* + 0.49995	*y* = 0.0063*x* + 0.36923	*y* = 0.0042*x* + 0.57967
		*y* = 0.00407*x*+ 0.59312	*y* = 0.00711*x* + 0.28882	*y* = 0.0034*x* + 0.65961
2	*f*_2A_/*f*_2P_ > *f*_1A_/*f*_1P_ > *f*_3A_/*f*_3P_	*y* = 0.00211*x* + 0.78838	*y* = 0.00188*x* + 0.81217	*y* = 0.00185*x* + 0.81323
3	*f*_1A_/*f*_1P_ > *f*_3A_/*f*_3P_ > *f*_2A_/*f*_2P_	*y* = 0.00175*x* + 0.82498	*y* = 0.00705*x* + 0.29453	*y* = 0.00333*x* + 0.66671
		*y* = 0.00063*x* + 0.93748	*y* = 0.009*x* + 0.09943	*y* = 0.00142*x* + 0.85808
		*y* = 0.00021*x* + 0.97854	*y* = 0.00369*x* + 0.63025	*y* = 0.00106*x* + 0.89359
4	*f*_3A_/*f*_3P_ > *f*_2A_/*f*_2P_ > *f*_1A_/*f*_1P_	*y* = 0.00748*x* + 0.25129	*y* = 0.00282*x* + 0.71745	*y* = 0.00015*x* + 0.98494
5	*f*_2A_/*f*_2P_ > *f*_3A_/*f*_3P_ > *f*_1A_/*f*_1P_	*y* = 0.00996*x* + 0.00402	*y* = 0.00131*x* + 0.86907	*y* = 0.00617*x* + 0.38288
		*y* = 0.01086*x* − 0.08609	*y* = 0.00298*x* + 0.70152	*y* = 0.00932*x* + 0.06758

**Table 7:** Accuracy of Level 2 damage identification.

Band	Set 1 (%)	Set 2 (%)	Set 3 (%)	Set 4 (%)	Aggregate (%)
1	97.67	96.97	97.67	97.30	97.35
2	100.00	100.00	100.00	100.00	100.00
3	96.55	97.53	97.59	97.37	97.50
4	88.00	88.00	89.29	96.15	91.14
5	97.67	100.00	100.00	97.44	98.95
Aggregate	96.50	97.00	97.00	97.50	97.17

**Table 8:** Best quantification relationship for Level 3 damage identification in each localization band.

Localization Band	Optimum Quantification Relation	Source
1	*y* = 0.00671*x* + 0.32903	Average of *i* = 2
2	*y* = 0.00188*x* + 0.81217	*i* = 2
3	*y* = 0.00658*x* + 0.3414	Average of *i* = 2
4	*y* = 0.00748*x* + 0.25129	*i* = 1
5	*y* = 0.01041*x* − 0.04104	Average of *i* = 1

**Table 9:** Accuracy of Level 3 damage identification.

Band	Set 1 (%)	Set 2 (%)	Set 3 (%)	Set 4 (%)	Aggregate (%)
1	90.16	88.50	87.46	91.78	89.25
2	75.19	63.39	68.42	70.79	67.53
3	87.53	89.76	89.35	86.42	88.51
4	93.22	93.22	92.93	92.31	92.82
5	95.94	93.68	96.06	94.24	94.66
Aggregate	88.41	85.71	86.84	87.11	86.55

**Table 10:** Geometric characteristics of the experimental test pipe.

Pipe	*D*_0_ (mm)	*T*_0_ (mm)	*L*_0_ (mm)	*M_d_* (mm)	*α_d_* (°)	*T_d_* (mm)	*L_d_* (mm)
Undamaged	103	6.5	900	-	-	-	-
Damaged	103	6.5	900	225	90	4	125

**Table 11:** Vibration modal frequencies and RRP frequency ratio.

Pipe	Modal Frequencies (Hz)									RRP Frequency Ratios		
	*f*_1A_	*f*_1_	*f*_1P_	*f*_2A_	*f*_2_	*f*_2P_	*f*_3A_	*f*_3_	*f*_3P_	*f*_1A_/*f*_1P_	*f*_2A_/*f*_2P_	*f*_3A_/*f*_3P_
Undamaged	-	355	-	-	1164	-	-	2180	-	1.0000	1.0000	1.0000
Damaged	348	-	436	1131	-	1224	2153	-	2260	0.7982	0.9240	0.9527

## Data Availability

Dataset available on request from the authors.

## Supplementary Materials

The following supporting information can be downloaded at: https://www.mdpi.com/article/10.3390/s25227020/s1, Table S1: Database of damage properties, geometric indices and LVMP frequencies; Table S2: Test cases and verification results.

## Nomenclature

*D*_0_	Outside diameter of pipe
*V*	Damage indices: volume
*V_0_*	Volume of undamaged pipe
*V_d_*	Volume of damage
*V_actual_*	Actual volume of damaged pipes
*V_predicted_*	Predicted volume of damaged pipes
*A*	Damage indices: Cross-sectional area
*A_0_*	Cross-sectional area of undamaged pipe
*A_d_*	Cross-sectional area of damage
*S*	Damage indices: Surface area
*S*_0_	Surface area of undamaged pipe
*S_d_*	Surface area of damage
*M_d_*	Axial distance from damage centroid to end of pipe
*X_d_*	Offset of damage centroid from centroid of undamaged pipe in X axis
*Z_d_*	Offset of damage centroid from centroid of undamaged pipe in Z axis
*P*_1_, *P*_2_, *P*_3_	Accuracy of Level 1, 2 and 3 damage identification prediction
*α_d_*	Angle subtended by damage
*ε*	Error in Level 3 damage identification prediction
*j*	Level 3 damage identification test case index
*N*_1_, *N*_2_	Number of cases in which Level 1 and 2 damage identification is correctly predicted
*N*	Total number of test cases in set (or sub-set)
*δ_x_*	Damage indices: cross-sectional symmetry
*δ_z_*	Damage indices: axial symmetry
*M_x_, M_y_, M_z_*	Shift in centroidal axis of pipe in X, Y and Z axes
