# Supplementary material for: Identification of Pipe Damage by Line-Structured Light and Regional Resonance Pairs
Source: Sensors (Basel). 2025 Nov 17;25(22):7020. doi: 10.3390/s25227020 (PMC12656081; doi:10.3390/s25227020)
Supplement: Supplementary file 1 [file sensors-25-07020-s001.zip › sensors-3946650-supplementary.pdf]

Supplementary material

Identification of pipe damage by line structured light and regional resonance pairs

Table S1 Database of damage properties, geometric indices and LVMP frequencies.

S/N	Damage Properties				Geometric Indices (%)					RRP Frequencies (Hz)					
	α_d (°)	M_d (mm)	L_d (mm)	T_d (mm)	V	A	S	δx	δz	f_{1A}	f_{1P}	f_{2A}	f_{2P}	f_{3A}	f_{3P}
1	22.5	90	45	0.65	99.967	99.337	99.687	94.52	99.987	643.489	643.605	1536.605	1537.062	2628.046	2628.778
2	45	90	45	0.65	99.934	98.674	99.375	97.223	99.973	643.247	643.481	1536.238	1537.052	2627.703	2629.001
3	67.5	90	45	0.65	99.901	98.011	99.062	95.853	99.96	643.021	643.344	1535.934	1536.97	2627.454	2629.091
4	90	90	45	0.65	99.867	97.348	98.75	95.084	99.947	642.837	643.188	1535.726	1536.85	2627.363	2629.125
5	22.5	90	45	1.3	99.934	98.683	99.687	89.107	99.974	643.254	643.495	1536.114	1537.159	2627.466	2629.147
6	45	90	45	1.3	99.868	97.365	99.375	94.443	99.947	642.699	643.236	1535.304	1537.101	2626.663	2629.508
7	67.5	90	45	1.3	99.802	96.048	99.062	91.644	99.921	642.216	642.958	1534.645	1536.972	2626.092	2629.711
8	90	90	45	1.3	99.737	94.731	98.75	90.023	99.894	641.791	642.61	1534.124	1536.671	2625.754	2629.635
9	22.5	90	45	1.95	99.902	98.037	99.687	83.763	99.961	642.991	643.365	1535.502	1537.241	2626.689	2629.484
10	45	90	45	1.95	99.804	96.073	99.375	91.659	99.921	642.094	642.971	1534.159	1537.196	2625.291	2630.053
11	67.5	90	45	1.95	99.705	94.11	99.062	87.371	99.882	641.246	642.497	1532.952	1536.914	2624.158	2630.197
12	90	90	45	1.95	99.607	92.146	98.75	84.812	99.842	640.53	641.917	1531.984	1536.396	2623.421	2629.922
13	22.5	90	45	2.6	99.87	97.399	99.687	78.488	99.948	642.669	643.15	1534.583	1537.256	2625.437	2629.716
14	45	90	45	2.6	99.74	94.798	99.375	88.874	99.896	641.294	642.559	1532.461	1537.095	2623.118	2630.294
15	67.5	90	45	2.6	99.61	92.197	99.062	83.033	99.843	639.987	641.844	1530.484	1536.62	2621.159	2630.282
16	90	90	45	2.6	99.48	89.596	98.75	79.439	99.791	638.855	640.955	1528.778	1535.744	2619.716	2629.557
17	22.5	90	90	0.65	99.934	99.337	99.375	94.52	99.973	643.249	643.503	1536.244	1536.997	2627.679	2628.919
18	45	90	90	0.65	99.867	98.674	98.75	97.223	99.947	642.762	643.244	1535.507	1536.874	2626.988	2629.191
19	67.5	90	90	0.65	99.801	98.011	98.125	95.853	99.92	642.358	643.008	1534.988	1536.756	2626.637	2629.438
20	90	90	90	0.65	99.735	97.348	97.5	95.084	99.894	641.978	642.699	1534.541	1536.446	2626.398	2629.365
21	22.5	90	90	1.3	99.868	98.683	99.375	89.107	99.947	642.787	643.247	1535.217	1537.03	2626.469	2629.413
22	45	90	90	1.3	99.737	97.365	98.75	94.443	99.894	641.751	642.729	1533.622	1536.755	2624.947	2629.847
23	67.5	90	90	1.3	99.605	96.048	98.125	91.644	99.841	640.83	642.206	1532.361	1536.364	2623.965	2629.993
24	90	90	90	1.3	99.473	94.731	97.5	90.023	99.788	640.007	641.565	1531.331	1535.678	2623.349	2629.654
25	22.5	90	90	1.95	99.804	98.037	99.375	83.763	99.921	642.32	642.976	1533.97	1537.078	2624.923	2629.91
26	45	90	90	1.95	99.607	96.073	98.75	91.659	99.842	640.72	642.222	1531.384	1536.706	2622.453	2630.512
27	67.5	90	90	1.95	99.411	94.11	98.125	87.371	99.763	639.189	641.349	1529.073	1535.962	2620.579	2630.395

28	90	90	90	1.95	99.215	92.146	97.5	84.812	99.683	637.839	640.316	1527.162	1534.786	2619.36	2629.542
29	22.5	90	90	2.6	99.74	97.399	99.375	78.488	99.896	641.773	642.539	1532.081	1536.989	2622.442	2630.185
30	45	90	90	2.6	99.48	94.798	98.75	88.874	99.791	639.447	641.412	1527.968	1536.29	2618.477	2630.573
31	67.5	90	90	2.6	99.22	92.197	98.125	83.033	99.685	637.173	640.125	1524.187	1535.029	2615.443	2629.921
32	90	90	90	2.6	98.96	89.596	97.5	79.439	99.579	635.135	638.617	1520.917	1533.125	2613.368	2628.157
33	22.5	90	135	0.65	99.901	99.337	99.062	94.52	99.96	643.002	643.377	1535.886	1536.808	2627.294	2628.848
34	45	90	135	0.65	99.801	98.674	98.125	97.223	99.92	642.325	643.06	1534.963	1536.573	2626.486	2629.132
35	67.5	90	135	0.65	99.702	98.011	97.187	95.853	99.88	641.697	642.699	1534.181	1536.224	2625.931	2629.208
36	90	90	135	0.65	99.602	97.348	96.25	95.084	99.84	641.199	642.301	1533.614	1535.834	2625.716	2629.171
37	22.5	90	135	1.3	99.802	98.683	99.062	89.107	99.921	642.304	643.003	1534.432	1536.635	2625.591	2629.236
38	45	90	135	1.3	99.605	97.365	98.125	94.443	99.841	640.888	642.359	1532.389	1536.08	2623.807	2629.614
39	67.5	90	135	1.3	99.407	96.048	97.187	91.644	99.761	639.554	641.574	1530.521	1535.255	2622.425	2629.447
40	90	90	135	1.3	99.21	94.731	96.25	90.023	99.681	638.382	640.676	1529.022	1534.142	2621.651	2628.755
41	22.5	90	135	1.95	99.705	98.037	99.062	83.763	99.882	641.648	642.611	1532.65	1536.54	2623.426	2629.707
42	45	90	135	1.95	99.411	96.073	98.125	91.659	99.763	639.394	641.529	1529.001	1535.481	2620.225	2629.832
43	67.5	90	135	1.95	99.116	94.11	97.187	87.371	99.643	637.249	640.316	1525.753	1533.997	2617.966	2629.102
44	90	90	135	1.95	98.822	92.146	96.25	84.812	99.523	635.333	638.886	1522.977	1532.039	2616.489	2627.508
45	22.5	90	135	2.6	99.61	97.399	99.062	78.488	99.843	640.872	641.96	1529.872	1536.185	2619.905	2629.789
46	45	90	135	2.6	99.22	94.798	98.125	88.874	99.685	637.691	640.357	1524.043	1534.443	2614.929	2629.342
47	67.5	90	135	2.6	98.83	92.197	97.187	83.033	99.526	634.604	638.6	1518.754	1532.046	2611.514	2627.655
48	90	90	135	2.6	98.439	89.596	96.25	79.439	99.366	631.779	636.562	1514.095	1528.905	2609.215	2624.568
49	22.5	135	45	0.65	99.967	99.337	99.687	94.52	99.98	643.635	643.661	1536.909	1537.191	2628.652	2628.968
50	45	135	45	0.65	99.934	98.674	99.375	97.223	99.96	643.549	643.586	1536.821	1537.318	2628.803	2629.43
51	67.5	135	45	0.65	99.901	98.011	99.062	95.853	99.94	643.469	643.508	1536.768	1537.4	2628.972	2629.832
52	90	135	45	0.65	99.867	97.348	98.75	95.084	99.92	643.384	643.426	1536.755	1537.441	2629.222	2630.169
53	22.5	135	45	1.3	99.934	98.683	99.687	89.107	99.96	643.545	643.622	1536.744	1537.412	2628.823	2629.436
54	45	135	45	1.3	99.868	97.365	99.375	94.443	99.921	643.367	643.436	1536.521	1537.624	2628.925	2630.29
55	67.5	135	45	1.3	99.802	96.048	99.062	91.644	99.881	643.206	643.273	1536.393	1537.807	2629.177	2631.068
56	90	135	45	1.3	99.737	94.731	98.75	90.023	99.841	643.044	643.118	1536.359	1537.905	2629.574	2631.722
57	22.5	135	45	1.95	99.902	98.037	99.687	83.763	99.941	643.453	643.563	1536.527	1537.612	2628.901	2629.873
58	45	135	45	1.95	99.804	96.073	99.375	91.659	99.882	643.174	643.294	1536.107	1537.991	2628.912	2631.07

59	67.5	135	45	1.95	99.705	94.11	99.062	87.371	99.823	642.89	643.005	1535.775	1538.212	2628.953	2632.06
60	90	135	45	1.95	99.607	92.146	98.75	84.812	99.763	642.605	642.726	1535.585	1538.272	2629.207	2632.834
61	22.5	135	45	2.6	99.87	97.399	99.687	78.488	99.922	643.277	643.467	1536.069	1537.744	2628.791	2630.046
62	45	135	45	2.6	99.74	94.798	99.375	88.874	99.844	642.842	643.037	1535.278	1538.177	2628.267	2631.38
63	67.5	135	45	2.6	99.61	92.197	99.062	83.033	99.765	642.398	642.575	1534.586	1538.379	2627.642	2632.374
64	90	135	45	2.6	99.48	89.596	98.75	79.439	99.686	641.937	642.113	1534.064	1538.303	2627.302	2632.98
65	22.5	135	90	0.65	99.934	99.337	99.375	94.52	99.96	643.505	643.521	1536.714	1537.173	2628.687	2629.19
66	45	135	90	0.65	99.867	98.674	98.75	97.223	99.92	643.364	643.403	1536.596	1537.463	2629.118	2630.029
67	67.5	135	90	0.65	99.801	98.011	98.125	95.853	99.88	643.231	643.263	1536.559	1537.658	2629.561	2630.824
68	90	135	90	0.65	99.735	97.348	97.5	95.084	99.84	643.093	643.12	1536.581	1537.758	2630.034	2631.47
69	22.5	135	90	1.3	99.868	98.683	99.375	89.107	99.921	643.354	643.446	1536.436	1537.58	2629.181	2629.988
70	45	135	90	1.3	99.737	97.365	98.75	94.443	99.841	642.985	643.081	1535.97	1537.932	2629.606	2631.328
71	67.5	135	90	1.3	99.605	96.048	98.125	91.644	99.762	642.695	642.757	1535.777	1538.235	2630.132	2632.553
72	90	135	90	1.3	99.473	94.731	97.5	90.023	99.682	642.374	642.441	1535.683	1538.374	2630.722	2633.222
73	22.5	135	90	1.95	99.804	98.037	99.375	83.763	99.882	643.137	643.34	1535.926	1537.935	2629.571	2630.556
74	45	135	90	1.95	99.607	96.073	98.75	91.659	99.763	642.617	642.81	1535.166	1538.533	2630.081	2632.43
75	67.5	135	90	1.95	99.411	94.11	98.125	87.371	99.644	642.1	642.242	1534.588	1538.847	2630.317	2633.345
76	90	135	90	1.95	99.215	92.146	97.5	84.812	99.525	641.572	641.67	1534.233	1538.85	2630.463	2632.16
77	22.5	135	90	2.6	99.74	97.399	99.375	78.488	99.844	642.778	643.164	1534.968	1538.15	2629.704	2630.653
78	45	135	90	2.6	99.48	94.798	98.75	88.874	99.686	641.955	642.345	1533.47	1538.767	2629.797	2632.33
79	67.5	135	90	2.6	99.22	92.197	98.125	83.033	99.528	641.148	641.453	1532.22	1538.956	2629.254	2630.963
80	90	135	90	2.6	98.96	89.596	97.5	79.439	99.369	640.335	640.546	1531.276	1538.65	2625.66	2628.321
81	22.5	135	135	0.65	99.901	99.337	99.062	94.52	99.94	643.447	643.452	1536.665	1537.254	2628.945	2629.323
82	45	135	135	0.65	99.801	98.674	98.125	97.223	99.88	643.206	643.243	1536.493	1537.506	2629.46	2630.283
83	67.5	135	135	0.65	99.702	98.011	97.187	95.853	99.82	642.948	643.024	1536.387	1537.65	2629.931	2631.13
84	90	135	135	0.65	99.602	97.348	96.25	95.084	99.76	642.768	642.866	1536.501	1537.838	2630.625	2632.019
85	22.5	135	135	1.3	99.802	98.683	99.062	89.107	99.881	643.155	643.208	1536.111	1537.543	2629.451	2629.941
86	45	135	135	1.3	99.605	97.365	98.125	94.443	99.762	642.674	642.731	1535.661	1537.955	2630.186	2631.625
87	67.5	135	135	1.3	99.407	96.048	97.187	91.644	99.642	642.116	642.256	1535.282	1538.138	2630.62	2632.611
88	90	135	135	1.3	99.21	94.731	96.25	90.023	99.522	641.589	641.787	1535.127	1538.149	2631.02	2632.48
89	22.5	135	135	1.95	99.705	98.037	99.062	83.763	99.823	642.86	643.021	1535.399	1537.936	2630.071	2630.444

90	45	135	135	1.95	99.411	96.073	98.125	91.659	99.644	642.101	642.124	1534.298	1538.35	2630.503	2632.249
91	67.5	135	135	1.95	99.116	94.11	97.187	87.371	99.465	641.192	641.34	1533.433	1538.414	2630.375	2631.815
92	90	135	135	1.95	98.822	92.146	96.25	84.812	99.285	640.276	640.568	1532.876	1538.114	2627.489	2629.9
93	22.5	135	135	2.6	99.61	97.399	99.062	78.488	99.765	642.316	642.721	1533.933	1538.084	2630.117	2630.283
94	45	135	135	2.6	99.22	94.798	98.125	88.874	99.528	641.136	641.378	1531.775	1538.322	2629.732	2631.123
95	67.5	135	135	2.6	98.83	92.197	97.187	83.033	99.289	639.95	639.995	1530.004	1537.977	2625.363	2628.515
96	90	135	135	2.6	98.439	89.596	96.25	79.439	99.049	638.53	638.823	1528.586	1537.067	2617.428	2626.385
97	22.5	180	45	0.65	99.967	99.337	99.687	94.52	99.973	643.671	643.742	1537.062	1537.222	2628.401	2629.31
98	45	180	45	0.65	99.934	98.674	99.375	97.223	99.947	643.627	643.776	1537.143	1537.409	2628.416	2630.061
99	67.5	180	45	0.65	99.901	98.011	99.062	95.853	99.92	643.604	643.783	1537.199	1537.592	2628.479	2630.709
100	90	180	45	0.65	99.867	97.348	98.75	95.084	99.894	643.587	643.788	1537.323	1537.739	2628.772	2631.195
101	22.5	180	45	1.3	99.934	98.683	99.687	89.107	99.947	643.633	643.822	1537.176	1537.444	2628.359	2630.232
102	45	180	45	1.3	99.868	97.365	99.375	94.443	99.894	643.54	643.853	1537.18	1537.8	2628.03	2631.736
103	67.5	180	45	1.3	99.802	96.048	99.062	91.644	99.842	643.488	643.882	1537.268	1538.147	2628.01	2633.037
104	90	180	45	1.3	99.737	94.731	98.75	90.023	99.789	643.466	643.897	1537.462	1538.431	2628.421	2634.019
105	22.5	180	45	1.95	99.902	98.037	99.687	83.763	99.921	643.586	643.883	1537.224	1537.635	2628.152	2631.18
106	45	180	45	1.95	99.804	96.073	99.375	91.659	99.843	643.447	643.963	1537.178	1538.163	2627.345	2633.475
107	67.5	180	45	1.95	99.705	94.11	99.062	87.371	99.764	643.34	644.009	1537.19	1538.603	2626.875	2635.366
108	90	180	45	1.95	99.607	92.146	98.75	84.812	99.685	643.273	644	1537.313	1538.923	2627.051	2636.75
109	22.5	180	45	2.6	99.87	97.399	99.687	78.488	99.896	643.459	643.912	1537.16	1537.659	2627.697	2631.974
110	45	180	45	2.6	99.74	94.798	99.375	88.874	99.791	643.226	644.008	1536.891	1538.246	2625.881	2634.949
111	67.5	180	45	2.6	99.61	92.197	99.062	83.033	99.687	643.028	644.037	1536.64	1538.69	2624.305	2637.342
112	90	180	45	2.6	99.48	89.596	98.75	79.439	99.582	642.881	643.998	1536.563	1538.938	2623.719	2639.046
113	22.5	180	90	0.65	99.934	99.337	99.375	94.52	99.947	643.638	643.764	1537.147	1537.429	2628.511	2629.978
114	45	180	90	0.65	99.867	98.674	98.75	97.223	99.894	643.529	643.782	1537.248	1537.738	2628.565	2631.234
115	67.5	180	90	0.65	99.801	98.011	98.125	95.853	99.841	643.517	643.819	1537.462	1538.174	2628.912	2632.527
116	90	180	90	0.65	99.735	97.348	97.5	95.084	99.787	643.483	643.822	1537.697	1538.461	2629.453	2633.423
117	22.5	180	90	1.3	99.868	98.683	99.375	89.107	99.894	643.487	643.802	1537.23	1537.679	2628.444	2631.353
118	45	180	90	1.3	99.737	97.365	98.75	94.443	99.789	643.337	643.872	1537.422	1538.425	2628.425	2634.028
119	67.5	180	90	1.3	99.605	96.048	98.125	91.644	99.683	643.254	643.921	1537.696	1539.151	2628.736	2636.298
120	90	180	90	1.3	99.473	94.731	97.5	90.023	99.576	643.198	643.935	1538.103	1539.725	2629.561	2637.92

121	22.5	180	90	1.95	99.804	98.037	99.375	83.763	99.843	643.416	643.946	1537.504	1538.075	2628.586	2633.021
122	45	180	90	1.95	99.607	96.073	98.75	91.659	99.685	643.159	644.052	1537.67	1539.101	2628.191	2636.876
123	67.5	180	90	1.95	99.411	94.11	98.125	87.371	99.526	642.968	644.096	1537.883	1540.024	2628.067	2639.625
124	90	180	90	1.95	99.215	92.146	97.5	84.812	99.367	642.85	644.057	1538.226	1540.779	2628.568	2640.387
125	22.5	180	90	2.6	99.74	97.399	99.375	78.488	99.791	643.155	643.987	1537.533	1538.063	2628.321	2634.273
126	45	180	90	2.6	99.48	94.798	98.75	88.874	99.582	642.721	644.091	1537.515	1539.205	2627.127	2638.868
127	67.5	180	90	2.6	99.22	92.197	98.125	83.033	99.371	642.374	644.084	1537.441	1540.192	2625.859	2640.099
128	90	180	90	2.6	98.96	89.596	97.5	79.439	99.159	642.112	643.946	1537.486	1540.919	2625.25	2636.943
129	22.5	180	135	0.65	99.901	99.337	99.062	94.52	99.92	643.576	643.736	1537.161	1537.516	2628.643	2630.189
130	45	180	135	0.65	99.801	98.674	98.125	97.223	99.841	643.489	643.781	1537.386	1538.073	2628.969	2631.861
131	67.5	180	135	0.65	99.702	98.011	97.187	95.853	99.761	643.436	643.794	1537.639	1538.609	2629.448	2633.35
132	90	180	135	0.65	99.602	97.348	96.25	95.084	99.681	643.457	643.826	1538.051	1539.164	2630.282	2634.669
133	22.5	180	135	1.3	99.802	98.683	99.062	89.107	99.842	643.422	643.791	1537.353	1537.957	2628.872	2631.892
134	45	180	135	1.3	99.605	97.365	98.125	94.443	99.683	643.24	643.85	1537.682	1539.038	2629.308	2635.096
135	67.5	180	135	1.3	99.407	96.048	97.187	91.644	99.523	643.088	643.851	1538.04	1539.987	2629.869	2637.413
136	90	180	135	1.3	99.21	94.731	96.25	90.023	99.363	643.007	643.815	1538.565	1540.827	2630.83	2638.383
137	22.5	180	135	1.95	99.705	98.037	99.062	83.763	99.764	643.252	643.915	1537.676	1538.338	2629.271	2633.604
138	45	180	135	1.95	99.411	96.073	98.125	91.659	99.526	642.904	643.949	1537.956	1539.762	2629.489	2637.879
139	67.5	180	135	1.95	99.116	94.11	97.187	87.371	99.287	642.642	643.903	1538.253	1541.064	2629.678	2639.21
140	90	180	135	1.95	98.822	92.146	96.25	84.812	99.046	642.455	643.76	1538.682	1542.134	2630.092	2635.73
141	22.5	180	135	2.6	99.61	97.399	99.062	78.488	99.687	642.872	643.93	1537.756	1538.248	2629.313	2634.817
142	45	180	135	2.6	99.22	94.798	98.125	88.874	99.371	642.27	643.894	1537.823	1539.785	2628.977	2639.019
143	67.5	180	135	2.6	98.83	92.197	97.187	83.033	99.053	641.783	643.703	1537.729	1541.136	2628.039	2634.903
144	90	180	135	2.6	98.439	89.596	96.25	79.439	98.732	641.414	643.379	1537.758	1542.189	2627.076	2627.772
145	22.5	225	45	0.65	99.967	99.337	99.687	94.52	99.967	643.731	643.783	1536.908	1537.464	2628.396	2628.92
146	45	225	45	0.65	99.934	98.674	99.375	97.223	99.934	643.739	643.848	1536.826	1537.846	2628.355	2629.309
147	67.5	225	45	0.65	99.901	98.011	99.062	95.853	99.9	643.756	643.901	1536.802	1538.148	2628.346	2629.616
148	90	225	45	0.65	99.867	97.348	98.75	95.084	99.867	643.794	643.946	1536.903	1538.377	2628.472	2629.879
149	22.5	225	45	1.3	99.934	98.683	99.687	89.107	99.934	643.75	643.895	1536.811	1537.947	2628.362	2629.402
150	45	225	45	1.3	99.868	97.365	99.375	94.443	99.868	643.768	644.01	1536.463	1538.724	2628.051	2630.194
151	67.5	225	45	1.3	99.802	96.048	99.062	91.644	99.802	643.82	644.122	1536.309	1539.364	2627.925	2630.874

152	90	225	45	1.3	99.737	94.731	98.75	90.023	99.736	643.885	644.222	1536.42	1539.758	2628.057	2631.335
153	22.5	225	45	1.95	99.902	98.037	99.687	83.763	99.902	643.754	643.987	1536.611	1538.418	2628.195	2629.855
154	45	225	45	1.95	99.804	96.073	99.375	91.659	99.803	643.791	644.195	1535.927	1539.619	2627.556	2631.083
155	67.5	225	45	1.95	99.705	94.11	99.062	87.371	99.705	643.855	644.366	1535.47	1540.555	2627.082	2632.087
156	90	225	45	1.95	99.607	92.146	98.75	84.812	99.606	643.934	644.497	1535.412	1541.066	2626.959	2632.686
157	22.5	225	45	2.6	99.87	97.399	99.687	78.488	99.87	643.702	644.058	1536.29	1538.806	2627.871	2630.159
158	45	225	45	2.6	99.74	94.798	99.375	88.874	99.739	643.709	644.315	1534.969	1540.357	2626.512	2631.709
159	67.5	225	45	2.6	99.61	92.197	99.062	83.033	99.608	643.729	644.519	1533.903	1541.484	2625.26	2632.88
160	90	225	45	2.6	99.48	89.596	98.75	79.439	99.477	643.8	644.668	1533.445	1542.058	2624.52	2633.554
161	22.5	225	90	0.65	99.934	99.337	99.375	94.52	99.934	643.704	643.798	1536.793	1537.781	2628.327	2629.183
162	45	225	90	0.65	99.867	98.674	98.75	97.223	99.867	643.745	643.939	1536.739	1538.552	2628.384	2629.958
163	67.5	225	90	0.65	99.801	98.011	98.125	95.853	99.801	643.823	644.066	1536.821	1539.239	2628.573	2630.711
164	90	225	90	0.65	99.735	97.348	97.5	95.084	99.734	643.892	644.155	1537.039	1539.682	2628.837	2631.221
165	22.5	225	90	1.3	99.868	98.683	99.375	89.107	99.868	643.738	643.994	1536.732	1538.674	2628.413	2630.026
166	45	225	90	1.3	99.737	97.365	98.75	94.443	99.736	643.778	644.193	1536.312	1540.15	2628.161	2631.507
167	67.5	225	90	1.3	99.605	96.048	98.125	91.644	99.603	643.885	644.417	1536.242	1541.411	2628.233	2632.885
168	90	225	90	1.3	99.473	94.731	97.5	90.023	99.47	644.038	644.603	1536.558	1542.305	2628.615	2634.002
169	22.5	225	90	1.95	99.804	98.037	99.375	83.763	99.803	643.765	644.183	1536.615	1539.594	2628.439	2630.887
170	45	225	90	1.95	99.607	96.073	98.75	91.659	99.606	643.837	644.546	1535.887	1541.856	2627.967	2633.189
171	67.5	225	90	1.95	99.411	94.11	98.125	87.371	99.407	643.97	644.866	1535.473	1543.705	2627.676	2635.267
172	90	225	90	1.95	99.215	92.146	97.5	84.812	99.208	644.151	645.112	1535.632	1544.936	2627.79	2636.902
173	22.5	225	90	2.6	99.74	97.399	99.375	78.488	99.739	643.65	644.303	1536.299	1540.291	2628.184	2631.374
174	45	225	90	2.6	99.48	94.798	98.75	88.874	99.477	643.666	644.762	1534.944	1543.203	2627.083	2634.325
175	67.5	225	90	2.6	99.22	92.197	98.125	83.033	99.214	643.761	645.131	1533.861	1545.541	2625.931	2637.012
176	90	225	90	2.6	98.96	89.596	97.5	79.439	98.949	643.927	645.401	1533.549	1547.036	2625.253	2639.133
177	22.5	225	135	0.65	99.901	99.337	99.062	94.52	99.9	643.733	643.862	1536.839	1538.111	2628.489	2629.428
178	45	225	135	0.65	99.801	98.674	98.125	97.223	99.801	643.8	644.039	1536.798	1539.176	2628.652	2630.444
179	67.5	225	135	0.65	99.702	98.011	97.187	95.853	99.701	643.897	644.181	1536.895	1540.089	2628.899	2631.392
180	90	225	135	0.65	99.602	97.348	96.25	95.084	99.601	644.066	644.367	1537.354	1540.858	2629.479	2632.312
181	22.5	225	135	1.3	99.802	98.683	99.062	89.107	99.802	643.748	644.055	1536.722	1539.239	2628.602	2630.364
182	45	225	135	1.3	99.605	97.365	98.125	94.443	99.603	643.886	644.388	1536.485	1541.391	2628.788	2632.443

183	67.5	225	135	1.3	99.407	96.048	97.187	91.644	99.404	644.035	644.662	1536.492	1543.113	2629.072	2634.24
184	90	225	135	1.3	99.21	94.731	96.25	90.023	99.203	644.261	644.913	1537.023	1544.405	2629.715	2635.838
185	22.5	225	135	1.95	99.705	98.037	99.062	83.763	99.705	643.774	644.309	1536.709	1540.442	2628.864	2631.349
186	45	225	135	1.95	99.411	96.073	98.125	91.659	99.407	643.909	644.765	1536.089	1543.549	2628.846	2634.338
187	67.5	225	135	1.95	99.116	94.11	97.187	87.371	99.109	644.114	645.152	1535.801	1546.092	2628.922	2637.035
188	90	225	135	1.95	98.822	92.146	96.25	84.812	98.808	644.378	645.465	1536.21	1547.841	2629.356	2638.999
189	22.5	225	135	2.6	99.61	97.399	99.062	78.488	99.608	643.602	644.457	1536.456	1541.327	2628.778	2631.797
190	45	225	135	2.6	99.22	94.798	98.125	88.874	99.214	643.664	645	1535.255	1545.287	2628.337	2635.573
191	67.5	225	135	2.6	98.83	92.197	97.187	83.033	98.816	643.832	645.428	1534.328	1548.454	2627.723	2638.716
192	90	225	135	2.6	98.439	89.596	96.25	79.439	98.415	644.091	645.728	1534.236	1550.544	2627.389	2638.649
193	22.5	270	45	0.65	99.967	99.337	99.687	94.52	99.96	643.779	643.79	1536.811	1537.497	2628.17	2628.518
194	45	270	45	0.65	99.934	98.674	99.375	97.223	99.92	643.842	643.86	1536.626	1537.929	2627.934	2628.537
195	67.5	270	45	0.65	99.901	98.011	99.062	95.853	99.881	643.902	643.929	1536.522	1538.253	2627.72	2628.475
196	90	270	45	0.65	99.867	97.348	98.75	95.084	99.841	643.959	643.994	1536.557	1538.445	2627.571	2628.375
197	22.5	270	45	1.3	99.934	98.683	99.687	89.107	99.921	643.859	643.889	1536.579	1538.04	2627.828	2628.644
198	45	270	45	1.3	99.868	97.365	99.375	94.443	99.842	643.955	644.008	1535.971	1538.889	2627.263	2628.536
199	67.5	270	45	1.3	99.802	96.048	99.062	91.644	99.762	644.08	644.163	1535.657	1539.566	2626.844	2628.438
200	90	270	45	1.3	99.737	94.731	98.75	90.023	99.683	644.212	644.297	1535.651	1539.921	2626.505	2628.241
201	22.5	270	45	1.95	99.902	98.037	99.687	83.763	99.882	643.946	643.971	1536.24	1538.628	2627.429	2628.7
202	45	270	45	1.95	99.804	96.073	99.375	91.659	99.764	644.101	644.186	1535.157	1539.947	2626.5	2628.611
203	67.5	270	45	1.95	99.705	94.11	99.062	87.371	99.646	644.248	644.387	1534.362	1540.901	2625.684	2628.319
204	90	270	45	1.95	99.607	92.146	98.75	84.812	99.527	644.411	644.577	1534.103	1541.361	2625.037	2627.853
205	22.5	270	45	2.6	99.87	97.399	99.687	78.488	99.844	643.995	644.001	1535.778	1539.115	2626.682	2628.675
206	45	270	45	2.6	99.74	94.798	99.375	88.874	99.687	644.136	644.245	1533.831	1540.824	2625.121	2628.324
207	67.5	270	45	2.6	99.61	92.197	99.062	83.033	99.53	644.281	644.479	1532.261	1542.037	2623.73	2627.715
208	90	270	45	2.6	99.48	89.596	98.75	79.439	99.372	644.443	644.68	1531.464	1542.495	2622.52	2626.821
209	22.5	270	90	0.65	99.934	99.337	99.375	94.52	99.92	643.79	643.836	1536.592	1537.889	2627.939	2628.416
210	45	270	90	0.65	99.867	98.674	98.75	97.223	99.841	643.935	643.981	1536.361	1538.706	2627.56	2628.503
211	67.5	270	90	0.65	99.801	98.011	98.125	95.853	99.761	644.081	644.154	1536.299	1539.412	2627.362	2628.536
212	90	270	90	0.65	99.735	97.348	97.5	95.084	99.681	644.203	644.301	1536.409	1539.835	2627.18	2628.374
213	22.5	270	90	1.3	99.868	98.683	99.375	89.107	99.842	643.973	644.008	1536.323	1538.903	2627.367	2628.635

214	45	270	90	1.3	99.737	97.365	98.75	94.443	99.683	644.174	644.269	1535.532	1540.498	2626.469	2628.546
215	67.5	270	90	1.3	99.605	96.048	98.125	91.644	99.524	644.417	644.578	1535.134	1541.814	2625.885	2628.401
216	90	270	90	1.3	99.473	94.731	97.5	90.023	99.364	644.695	644.868	1535.281	1542.609	2625.476	2628.118
217	22.5	270	90	1.95	99.804	98.037	99.375	83.763	99.764	644.15	644.167	1535.991	1539.945	2626.59	2628.825
218	45	270	90	1.95	99.607	96.073	98.75	91.659	99.527	644.488	644.606	1534.648	1542.445	2625.199	2628.733
219	67.5	270	90	1.95	99.411	94.11	98.125	87.371	99.289	644.82	645.032	1533.708	1544.338	2624.092	2628.334
220	90	270	90	1.95	99.215	92.146	97.5	84.812	99.05	645.162	645.442	1533.537	1545.439	2623.31	2627.593
221	22.5	270	90	2.6	99.74	97.399	99.375	78.488	99.687	644.214	644.253	1535.455	1540.829	2625.191	2628.778
222	45	270	90	2.6	99.48	94.798	98.75	88.874	99.372	644.64	644.729	1533.203	1544.071	2622.833	2628.376
223	67.5	270	90	2.6	99.22	92.197	98.125	83.033	99.056	644.994	645.247	1531.343	1546.509	2620.977	2627.445
224	90	270	90	2.6	98.96	89.596	97.5	79.439	98.738	645.363	645.724	1530.533	1547.798	2619.516	2625.988
225	22.5	270	135	0.65	99.901	99.337	99.062	94.52	99.881	643.87	643.908	1536.588	1538.228	2627.934	2628.492
226	45	270	135	0.65	99.801	98.674	98.125	97.223	99.761	644.061	644.153	1536.308	1539.408	2627.659	2628.557
227	67.5	270	135	0.65	99.702	98.011	97.187	95.853	99.641	644.252	644.388	1536.239	1540.344	2627.473	2628.556
228	90	270	135	0.65	99.602	97.348	96.25	95.084	99.521	644.497	644.664	1536.561	1541.059	2627.502	2628.572
229	22.5	270	135	1.3	99.802	98.683	99.062	89.107	99.762	644.064	644.12	1536.207	1539.525	2627.227	2628.583
230	45	270	135	1.3	99.605	97.365	98.125	94.443	99.524	644.435	644.602	1535.507	1541.868	2626.577	2628.638
231	67.5	270	135	1.3	99.407	96.048	97.187	91.644	99.284	644.777	645.047	1535.122	1543.646	2626.096	2628.428
232	90	270	135	1.3	99.21	94.731	96.25	90.023	99.044	645.163	645.49	1535.417	1544.799	2625.893	2628.118
233	22.5	270	135	1.95	99.705	98.037	99.062	83.763	99.646	644.323	644.368	1535.936	1540.951	2626.377	2628.825
234	45	270	135	1.95	99.411	96.073	98.125	91.659	99.289	644.81	645.029	1534.572	1544.342	2625.086	2628.597
235	67.5	270	135	1.95	99.116	94.11	97.187	87.371	98.93	645.289	645.684	1533.676	1546.936	2624.274	2628.053
236	90	270	135	1.95	98.822	92.146	96.25	84.812	98.569	645.796	646.307	1533.711	1548.479	2623.857	2627.231
237	22.5	270	135	2.6	99.61	97.399	99.062	78.488	99.53	644.437	644.478	1535.436	1542.108	2624.631	2628.72
238	45	270	135	2.6	99.22	94.798	98.125	88.874	99.056	645.036	645.217	1533.185	1546.449	2622.353	2627.893
239	67.5	270	135	2.6	98.83	92.197	97.187	83.033	98.579	645.557	646.015	1531.389	1549.68	2620.938	2626.493
240	90	270	135	2.6	98.439	89.596	96.25	79.439	98.098	646.105	646.769	1530.782	1551.416	2620.104	2624.719
241	22.5	315	45	0.65	99.967	99.337	99.687	94.52	99.954	643.748	643.857	1536.806	1537.365	2627.835	2628.507
242	45	315	45	0.65	99.934	98.674	99.375	97.223	99.907	643.783	643.977	1536.617	1537.645	2627.253	2628.504
243	67.5	315	45	0.65	99.901	98.011	99.062	95.853	99.861	643.819	644.086	1536.47	1537.851	2626.758	2628.347
244	90	315	45	0.65	99.867	97.348	98.75	95.084	99.814	643.898	644.183	1536.488	1537.972	2626.421	2628.16

245	22.5	315	45	1.3	99.934	98.683	99.687	89.107	99.908	643.801	644.013	1536.598	1537.75	2627.087	2628.663
246	45	315	45	1.3	99.868	97.365	99.375	94.443	99.815	643.805	644.259	1536.003	1538.323	2625.843	2628.539
247	67.5	315	45	1.3	99.802	96.048	99.062	91.644	99.723	643.881	644.496	1535.655	1538.762	2624.828	2628.32
248	90	315	45	1.3	99.737	94.731	98.75	90.023	99.63	644.005	644.682	1535.555	1538.954	2624.056	2627.842
249	22.5	315	45	1.95	99.902	98.037	99.687	83.763	99.862	643.823	644.176	1536.291	1538.159	2626.219	2628.761
250	45	315	45	1.95	99.804	96.073	99.375	91.659	99.725	643.821	644.572	1535.255	1539.066	2624.225	2628.682
251	67.5	315	45	1.95	99.705	94.11	99.062	87.371	99.586	643.861	644.903	1534.457	1539.657	2622.456	2628.244
252	90	315	45	1.95	99.607	92.146	98.75	84.812	99.448	643.99	645.152	1534.087	1539.839	2621.053	2627.39
253	22.5	315	45	2.6	99.87	97.399	99.687	78.488	99.818	643.802	644.291	1535.856	1538.475	2624.962	2628.779
254	45	315	45	2.6	99.74	94.798	99.375	88.874	99.635	643.688	644.778	1534.047	1539.592	2621.843	2628.527
255	67.5	315	45	2.6	99.61	92.197	99.062	83.033	99.452	643.633	645.194	1532.538	1540.306	2619.061	2627.783
256	90	315	45	2.6	99.48	89.596	98.75	79.439	99.268	643.695	645.478	1531.647	1540.39	2616.731	2626.381
257	22.5	315	90	0.65	99.934	99.337	99.375	94.52	99.907	643.739	643.955	1536.576	1537.607	2627.3	2628.357
258	45	315	90	0.65	99.867	98.674	98.75	97.223	99.814	643.848	644.222	1536.331	1538.198	2626.38	2628.365
259	67.5	315	90	0.65	99.801	98.011	98.125	95.853	99.721	643.99	644.503	1536.234	1538.73	2625.732	2628.249
260	90	315	90	0.65	99.735	97.348	97.5	95.084	99.628	644.13	644.68	1536.249	1538.939	2625.095	2627.833
261	22.5	315	90	1.3	99.868	98.683	99.375	89.107	99.815	643.865	644.265	1536.306	1538.342	2626.026	2628.527
262	45	315	90	1.3	99.737	97.365	98.75	94.443	99.63	643.954	644.756	1535.482	1539.425	2623.912	2628.229
263	67.5	315	90	1.3	99.605	96.048	98.125	91.644	99.445	644.133	645.246	1534.983	1540.304	2622.263	2627.708
264	90	315	90	1.3	99.473	94.731	97.5	90.023	99.258	644.409	645.637	1534.922	1540.734	2620.949	2626.819
265	22.5	315	90	1.95	99.804	98.037	99.375	83.763	99.725	643.97	644.571	1535.976	1539.073	2624.433	2628.653
266	45	315	90	1.95	99.607	96.073	98.75	91.659	99.448	644.101	645.361	1534.608	1540.775	2621.071	2628.194
267	67.5	315	90	1.95	99.411	94.11	98.125	87.371	99.17	644.288	646.067	1533.547	1541.981	2618.218	2627.177
268	90	315	90	1.95	99.215	92.146	97.5	84.812	98.892	644.611	646.634	1533.124	1542.504	2615.906	2625.532
269	22.5	315	90	2.6	99.74	97.399	99.375	78.488	99.635	643.995	644.78	1535.454	1539.615	2622.062	2628.529
270	45	315	90	2.6	99.48	94.798	98.75	88.874	99.268	644.055	645.797	1533.223	1541.77	2616.908	2627.535
271	67.5	315	90	2.6	99.22	92.197	98.125	83.033	98.899	644.151	646.685	1531.274	1543.192	2612.388	2625.653
272	90	315	90	2.6	98.96	89.596	97.5	79.439	98.528	644.424	647.387	1530.165	1543.671	2608.601	2622.894
273	22.5	315	135	0.65	99.901	99.337	99.062	94.52	99.861	643.809	644.083	1536.547	1537.857	2627.165	2628.33
274	45	315	135	0.65	99.801	98.674	98.125	97.223	99.721	643.95	644.493	1536.209	1538.712	2626.199	2628.18
275	67.5	315	135	0.65	99.702	98.011	97.187	95.853	99.581	644.123	644.868	1536.038	1539.381	2625.417	2627.877

276	90	315	135	0.65	99.602	97.348	96.25	95.084	99.441	644.41	645.216	1536.232	1539.839	2624.884	2627.533
277	22.5	315	135	1.3	99.802	98.683	99.062	89.107	99.723	643.936	644.51	1536.131	1538.814	2625.641	2628.23
278	45	315	135	1.3	99.605	97.365	98.125	94.443	99.445	644.183	645.323	1535.32	1540.466	2623.486	2627.754
279	67.5	315	135	1.3	99.407	96.048	97.187	91.644	99.165	644.466	646.017	1534.757	1541.609	2621.588	2626.865
280	90	315	135	1.3	99.21	94.731	96.25	90.023	98.885	644.894	646.633	1534.766	1542.272	2620.185	2625.693
281	22.5	315	135	1.95	99.705	98.037	99.062	83.763	99.586	644.12	644.953	1535.823	1539.794	2623.743	2628.237
282	45	315	135	1.95	99.411	96.073	98.125	91.659	99.17	644.394	646.134	1534.32	1542.125	2620.012	2627.061
283	67.5	315	135	1.95	99.116	94.11	97.187	87.371	98.752	644.752	647.202	1533.188	1543.77	2616.907	2625.345
284	90	315	135	1.95	98.822	92.146	96.25	84.812	98.331	645.276	648.088	1532.814	1544.517	2614.39	2623.105
285	22.5	315	135	2.6	99.61	97.399	99.062	78.488	99.452	644.195	645.268	1535.285	1540.511	2620.806	2627.822
286	45	315	135	2.6	99.22	94.798	98.125	88.874	98.899	644.43	646.771	1532.883	1543.367	2614.943	2625.424
287	67.5	315	135	2.6	98.83	92.197	97.187	83.033	98.342	644.729	648.134	1530.815	1545.239	2610.054	2622.224
288	90	315	135	2.6	98.439	89.596	96.25	79.439	97.78	645.254	649.269	1529.727	1545.877	2605.993	2618.402
289	22.5	360	45	0.65	99.967	99.337	99.687	94.52	99.947	643.717	643.915	1536.905	1537.111	2628.171	2628.3
290	45	360	45	0.65	99.934	98.674	99.375	97.223	99.894	643.711	644.086	1536.776	1537.171	2627.88	2628.074
291	67.5	360	45	0.65	99.901	98.011	99.062	95.853	99.841	643.726	644.223	1536.653	1537.183	2627.571	2627.791
292	90	360	45	0.65	99.867	97.348	98.75	95.084	99.788	643.792	644.339	1536.608	1537.194	2627.342	2627.558
293	22.5	360	45	1.3	99.934	98.683	99.687	89.107	99.895	643.715	644.129	1536.809	1537.215	2627.792	2628.152
294	45	360	45	1.3	99.868	97.365	99.375	94.443	99.789	643.627	644.473	1536.418	1537.318	2627.129	2627.477
295	67.5	360	45	1.3	99.802	96.048	99.062	91.644	99.683	643.641	644.775	1536.178	1537.385	2626.53	2626.904
296	90	360	45	1.3	99.737	94.731	98.75	90.023	99.577	643.742	644.992	1536.031	1537.359	2625.962	2626.34
297	22.5	360	45	1.95	99.902	98.037	99.687	83.763	99.843	643.674	644.358	1536.647	1537.323	2627.341	2627.884
298	45	360	45	1.95	99.804	96.073	99.375	91.659	99.685	643.498	644.901	1536.018	1537.494	2626.244	2626.738
299	67.5	360	45	1.95	99.705	94.11	99.062	87.371	99.527	643.409	645.334	1535.474	1537.518	2625.142	2625.534
300	90	360	45	1.95	99.607	92.146	98.75	84.812	99.369	643.477	645.622	1535.11	1537.369	2624.036	2624.447
301	22.5	360	45	2.6	99.87	97.399	99.687	78.488	99.792	643.584	644.539	1536.396	1537.29	2626.54	2627.458
302	45	360	45	2.6	99.74	94.798	99.375	88.874	99.583	643.189	645.243	1535.304	1537.413	2624.737	2625.349
303	67.5	360	45	2.6	99.61	92.197	99.062	83.033	99.373	642.894	645.795	1534.291	1537.325	2622.896	2623.119
304	90	360	45	2.6	99.48	89.596	98.75	79.439	99.163	642.824	646.118	1533.525	1536.946	2620.967	2621.211
305	22.5	360	90	0.65	99.934	99.337	99.375	94.52	99.894	643.683	644.069	1536.723	1537.137	2627.877	2627.997
306	45	360	90	0.65	99.867	98.674	98.75	97.223	99.788	643.719	644.421	1536.547	1537.286	2627.408	2627.62

307	67.5	360	90	0.65	99.801	98.011	98.125	95.853	99.681	643.797	644.744	1536.418	1537.427	2627.046	2627.226
308	90	360	90	0.65	99.735	97.348	97.5	95.084	99.575	643.955	644.982	1536.374	1537.451	2626.637	2626.824
309	22.5	360	90	1.3	99.868	98.683	99.375	89.107	99.789	643.728	644.494	1536.6	1537.345	2627.224	2627.716
310	45	360	90	1.3	99.737	97.365	98.75	94.443	99.577	643.668	645.179	1536.023	1537.56	2626.095	2626.571
311	67.5	360	90	1.3	99.605	96.048	98.125	91.644	99.365	643.755	645.813	1535.637	1537.755	2625.172	2625.518
312	90	360	90	1.3	99.473	94.731	97.5	90.023	99.152	644.004	646.276	1535.428	1537.731	2624.232	2624.518
313	22.5	360	90	1.95	99.804	98.037	99.375	83.763	99.685	643.747	644.929	1536.443	1537.517	2626.348	2627.337
314	45	360	90	1.95	99.607	96.073	98.75	91.659	99.369	643.626	646.008	1535.551	1537.848	2624.473	2625.412
315	67.5	360	90	1.95	99.411	94.11	98.125	87.371	99.052	643.624	646.935	1534.757	1537.992	2622.736	2623.38
316	90	360	90	1.95	99.215	92.146	97.5	84.812	98.733	643.866	647.616	1534.206	1537.837	2621.053	2621.495
317	22.5	360	90	2.6	99.74	97.399	99.375	78.488	99.583	643.672	645.269	1536.104	1537.395	2624.761	2626.601
318	45	360	90	2.6	99.48	94.798	98.75	88.874	99.163	643.336	646.701	1534.639	1537.678	2621.694	2623.309
319	67.5	360	90	2.6	99.22	92.197	98.125	83.033	98.742	643.113	647.899	1533.208	1537.629	2618.732	2619.861
320	90	360	90	2.6	98.96	89.596	97.5	79.439	98.318	643.211	648.752	1532.074	1537.155	2615.875	2616.666
321	22.5	360	135	0.65	99.901	99.337	99.062	94.52	99.841	643.722	644.236	1536.683	1537.211	2627.785	2627.842
322	45	360	135	0.65	99.801	98.674	98.125	97.223	99.681	643.797	644.789	1536.459	1537.513	2627.283	2627.366
323	67.5	360	135	0.65	99.702	98.011	97.187	95.853	99.521	643.922	645.258	1536.273	1537.709	2626.688	2626.936
324	90	360	135	0.65	99.602	97.348	96.25	95.084	99.361	644.204	645.667	1536.303	1537.862	2626.257	2626.596
325	22.5	360	135	1.3	99.802	98.683	99.062	89.107	99.683	643.754	644.833	1536.413	1537.476	2626.97	2627.23
326	45	360	135	1.3	99.605	97.365	98.125	94.443	99.365	643.833	645.926	1535.846	1537.971	2625.831	2625.9
327	67.5	360	135	1.3	99.407	96.048	97.187	91.644	99.046	644.012	646.844	1535.34	1538.224	2624.349	2624.807
328	90	360	135	1.3	99.21	94.731	96.25	90.023	98.725	644.423	647.584	1535.1	1538.277	2623.017	2623.805
329	22.5	360	135	1.95	99.705	98.037	99.062	83.763	99.527	643.844	645.473	1536.271	1537.733	2625.9	2626.738
330	45	360	135	1.95	99.411	96.073	98.125	91.659	99.052	643.832	647.081	1535.186	1538.267	2623.805	2624.135
331	67.5	360	135	1.95	99.116	94.11	97.187	87.371	98.574	643.973	648.479	1534.192	1538.511	2621.441	2621.86
332	90	360	135	1.95	98.822	92.146	96.25	84.812	98.093	644.439	649.562	1533.539	1538.363	2618.924	2619.993
333	22.5	360	135	2.6	99.61	97.399	99.062	78.488	99.373	643.814	645.98	1535.891	1537.568	2623.841	2625.761
334	45	360	135	2.6	99.22	94.798	98.125	88.874	98.742	643.617	648.092	1534.117	1537.976	2620.233	2621.59
335	67.5	360	135	2.6	98.83	92.197	97.187	83.033	98.105	643.576	649.923	1532.369	1537.957	2616.84	2617.236
336	90	360	135	2.6	98.439	89.596	96.25	79.439	97.463	643.946	651.331	1530.997	1537.398	2612.981	2613.672
337	22.5	405	45	0.65	99.967	99.337	99.687	94.52	99.94	643.679	643.949	1536.863	1536.988	2627.962	2628.824

338	45	405	45	0.65	99.934	98.674	99.375	97.223	99.881	643.654	644.155	1536.724	1536.962	2627.498	2629.116
339	67.5	405	45	0.65	99.901	98.011	99.062	95.853	99.821	643.65	644.318	1536.583	1536.877	2627.088	2629.258
340	90	405	45	0.65	99.867	97.348	98.75	95.084	99.761	643.712	644.439	1536.488	1536.807	2626.916	2629.279
341	22.5	405	45	1.3	99.934	98.683	99.687	89.107	99.881	643.647	644.203	1536.706	1537.048	2627.441	2629.202
342	45	405	45	1.3	99.868	97.365	99.375	94.443	99.763	643.486	644.613	1536.389	1536.889	2626.105	2629.749
343	67.5	405	45	1.3	99.802	96.048	99.062	91.644	99.644	643.45	644.962	1536.139	1536.783	2625.168	2630.089
344	90	405	45	1.3	99.737	94.731	98.75	90.023	99.524	643.525	645.191	1535.923	1536.615	2624.628	2630.059
345	22.5	405	45	1.95	99.902	98.037	99.687	83.763	99.823	643.564	644.479	1536.544	1537.052	2626.737	2629.602
346	45	405	45	1.95	99.804	96.073	99.375	91.659	99.646	643.256	645.122	1536.054	1536.881	2624.414	2630.432
347	67.5	405	45	1.95	99.705	94.11	99.062	87.371	99.468	643.064	645.621	1535.585	1536.637	2622.486	2630.831
348	90	405	45	1.95	99.607	92.146	98.75	84.812	99.29	643.078	645.929	1535.179	1536.322	2621.306	2630.623
349	22.5	405	45	2.6	99.87	97.399	99.687	78.488	99.766	643.418	644.705	1536.192	1536.994	2625.792	2629.774
350	45	405	45	2.6	99.74	94.798	99.375	88.874	99.531	642.806	645.548	1535.401	1536.668	2621.893	2630.734
351	67.5	405	45	2.6	99.61	92.197	99.062	83.033	99.295	642.323	646.186	1534.617	1536.211	2618.443	2631.083
352	90	405	45	2.6	99.48	89.596	98.75	79.439	99.059	642.159	646.544	1533.923	1535.668	2616.192	2630.547
353	22.5	405	90	0.65	99.934	99.337	99.375	94.52	99.881	643.628	644.137	1536.683	1536.876	2627.496	2629.006
354	45	405	90	0.65	99.867	98.674	98.75	97.223	99.761	643.626	644.556	1536.453	1536.848	2626.745	2629.547
355	67.5	405	90	0.65	99.801	98.011	98.125	95.853	99.641	643.679	644.926	1536.299	1536.777	2626.191	2629.97
356	90	405	90	0.65	99.735	97.348	97.5	95.084	99.521	643.825	645.187	1536.138	1536.658	2625.902	2630.053
357	22.5	405	90	1.3	99.868	98.683	99.375	89.107	99.763	643.612	644.64	1536.408	1536.936	2626.68	2629.662
358	45	405	90	1.3	99.737	97.365	98.75	94.443	99.524	643.45	645.452	1535.835	1536.701	2624.711	2630.646
359	67.5	405	90	1.3	99.605	96.048	98.125	91.644	99.286	643.451	646.172	1535.417	1536.467	2623.225	2631.386
360	90	405	90	1.3	99.473	94.731	97.5	90.023	99.046	643.67	646.683	1535.045	1536.18	2622.418	2631.545
361	22.5	405	90	1.95	99.804	98.037	99.375	83.763	99.646	643.565	645.159	1536.054	1536.982	2625.773	2630.265
362	45	405	90	1.95	99.607	96.073	98.75	91.659	99.29	643.248	646.449	1535.202	1536.673	2622.509	2631.812
363	67.5	405	90	1.95	99.411	94.11	98.125	87.371	98.933	643.09	647.515	1534.428	1536.227	2619.748	2632.812
364	90	405	90	1.95	99.215	92.146	97.5	84.812	98.575	643.26	648.26	1533.741	1535.655	2618.028	2632.978
365	22.5	405	90	2.6	99.74	97.399	99.375	78.488	99.531	643.424	645.604	1535.364	1536.884	2624.553	2630.466
366	45	405	90	2.6	99.48	94.798	98.75	88.874	99.059	642.783	647.311	1533.979	1536.321	2619.487	2632.256
367	67.5	405	90	2.6	99.22	92.197	98.125	83.033	98.584	642.301	648.711	1532.688	1535.528	2614.928	2633.31
368	90	405	90	2.6	98.96	89.596	97.5	79.439	98.108	642.254	649.655	1531.506	1534.509	2611.751	2633.304

369	22.5	405	135	0.65	99.901	99.337	99.062	94.52	99.821	643.654	644.352	1536.645	1536.869	2627.278	2629.133
370	45	405	135	0.65	99.801	98.674	98.125	97.223	99.641	643.662	644.986	1536.414	1536.774	2626.267	2629.846
371	67.5	405	135	0.65	99.702	98.011	97.187	95.853	99.461	643.754	645.523	1536.212	1536.64	2625.505	2630.333
372	90	405	135	0.65	99.602	97.348	96.25	95.084	99.281	644.04	645.97	1536.118	1536.588	2625.257	2630.583
373	22.5	405	135	1.3	99.802	98.683	99.062	89.107	99.644	643.614	645.046	1536.239	1536.801	2626.144	2629.793
374	45	405	135	1.3	99.605	97.365	98.125	94.443	99.286	643.558	646.335	1535.718	1536.568	2623.933	2631.225
375	67.5	405	135	1.3	99.407	96.048	97.187	91.644	98.927	643.635	647.386	1535.17	1536.185	2622.08	2632.12
376	90	405	135	1.3	99.21	94.731	96.25	90.023	98.566	644.021	648.199	1534.746	1535.795	2621.082	2632.469
377	22.5	405	135	1.95	99.705	98.037	99.062	83.763	99.468	643.624	645.813	1535.81	1536.857	2625.164	2630.478
378	45	405	135	1.95	99.411	96.073	98.125	91.659	98.933	643.372	647.713	1534.773	1536.308	2621.398	2632.382
379	67.5	405	135	1.95	99.116	94.11	97.187	87.371	98.395	643.351	649.341	1533.868	1535.637	2618.25	2633.758
380	90	405	135	1.95	98.822	92.146	96.25	84.812	97.854	643.735	650.538	1533.026	1534.799	2616.188	2634.171
381	22.5	405	135	2.6	99.61	97.399	99.062	78.488	99.295	643.509	646.461	1534.871	1536.679	2623.816	2630.521
382	45	405	135	2.6	99.22	94.798	98.125	88.874	98.584	642.966	648.981	1533.133	1535.719	2618.278	2632.661
383	67.5	405	135	2.6	98.83	92.197	97.187	83.033	97.868	642.641	651.129	1531.574	1534.475	2613.293	2633.916
384	90	405	135	2.6	98.439	89.596	96.25	79.439	97.146	642.872	652.706	1530.143	1533.012	2609.724	2632.701
385	22.5	450	45	0.65	99.967	99.337	99.687	94.52	99.934	643.671	643.957	1536.758	1537.038	2627.827	2629.14
386	45	450	45	0.65	99.934	98.674	99.375	97.223	99.867	643.631	644.182	1536.55	1537.041	2627.236	2629.742
387	67.5	450	45	0.65	99.901	98.011	99.062	95.853	99.801	643.63	644.352	1536.346	1536.992	2626.798	2630.106
388	90	450	45	0.65	99.867	97.348	98.75	95.084	99.734	643.69	644.479	1536.218	1536.915	2626.663	2630.271
389	22.5	450	45	1.3	99.934	98.683	99.687	89.107	99.868	643.621	644.231	1536.506	1537.144	2627.125	2629.912
390	45	450	45	1.3	99.868	97.365	99.375	94.443	99.736	643.433	644.657	1536.005	1537.092	2625.515	2631.055
391	67.5	450	45	1.3	99.802	96.048	99.062	91.644	99.604	643.372	645.025	1535.635	1537.034	2624.419	2631.879
392	90	450	45	1.3	99.737	94.731	98.75	90.023	99.472	643.452	645.256	1535.348	1536.888	2623.944	2632.133
393	22.5	450	45	1.95	99.902	98.037	99.687	83.763	99.803	643.514	644.516	1536.218	1537.209	2626.209	2630.724
394	45	450	45	1.95	99.804	96.073	99.375	91.659	99.607	643.16	645.196	1535.471	1537.237	2623.449	2632.555
395	67.5	450	45	1.95	99.705	94.11	99.062	87.371	99.409	642.935	645.721	1534.814	1537.135	2621.272	2633.734
396	90	450	45	1.95	99.607	92.146	98.75	84.812	99.212	642.928	646.035	1534.311	1536.845	2620.062	2633.998
397	22.5	450	45	2.6	99.87	97.399	99.687	78.488	99.74	643.356	644.764	1535.754	1537.249	2625.097	2631.424
398	45	450	45	2.6	99.74	94.798	99.375	88.874	99.478	642.665	645.652	1534.587	1537.246	2620.566	2633.825
399	67.5	450	45	2.6	99.61	92.197	99.062	83.033	99.217	642.12	646.326	1533.548	1537.044	2616.704	2635.326

400	90	450	45	2.6	99.48	89.596	98.75	79.439	98.954	641.914	646.683	1532.715	1536.586	2614.291	2635.48
401	22.5	450	90	0.65	99.934	99.337	99.375	94.52	99.867	643.602	644.151	1536.481	1536.931	2627.254	2629.545
402	45	450	90	0.65	99.867	98.674	98.75	97.223	99.734	643.58	644.6	1536.115	1536.958	2626.331	2630.618
403	67.5	450	90	0.65	99.801	98.011	98.125	95.853	99.601	643.632	644.984	1535.838	1536.932	2625.739	2631.434
404	90	450	90	0.65	99.735	97.348	97.5	95.084	99.468	643.769	645.256	1535.612	1536.779	2625.51	2631.782
405	22.5	450	90	1.3	99.868	98.683	99.375	89.107	99.736	643.569	644.696	1536.041	1537.083	2626.229	2630.908
406	45	450	90	1.3	99.737	97.365	98.75	94.443	99.472	643.361	645.549	1535.15	1536.981	2623.903	2632.984
407	67.5	450	90	1.3	99.605	96.048	98.125	91.644	99.206	643.339	646.312	1534.516	1536.839	2622.286	2634.63
408	90	450	90	1.3	99.473	94.731	97.5	90.023	98.941	643.546	646.829	1533.994	1536.53	2621.601	2635.338
409	22.5	450	90	1.95	99.804	98.037	99.375	83.763	99.607	643.496	645.241	1535.47	1537.215	2625.101	2632.275
410	45	450	90	1.95	99.607	96.073	98.75	91.659	99.212	643.112	646.605	1534.152	1537.171	2621.334	2635.654
411	67.5	450	90	1.95	99.411	94.11	98.125	87.371	98.815	642.901	647.721	1533.024	1536.891	2618.322	2638.136
412	90	450	90	1.95	99.215	92.146	97.5	84.812	98.417	643.038	648.491	1532.145	1536.312	2616.713	2639.28
413	22.5	450	90	2.6	99.74	97.399	99.375	78.488	99.478	643.335	645.72	1534.544	1537.229	2623.702	2633.426
414	45	450	90	2.6	99.48	94.798	98.75	88.874	98.954	642.579	647.528	1532.517	1537.054	2617.921	2637.956
415	67.5	450	90	2.6	99.22	92.197	98.125	83.033	98.427	641.987	648.985	1530.716	1536.519	2612.903	2641.282
416	90	450	90	2.6	98.96	89.596	97.5	79.439	97.897	641.893	649.966	1529.291	1535.578	2609.828	2642.838
417	22.5	450	135	0.65	99.901	99.337	99.062	94.52	99.801	643.628	644.39	1536.411	1536.951	2627.03	2629.8
418	45	450	135	0.65	99.801	98.674	98.125	97.223	99.601	643.627	645.05	1535.964	1536.948	2625.875	2631.092
419	67.5	450	135	0.65	99.702	98.011	97.187	95.853	99.402	643.692	645.611	1535.608	1536.813	2625.018	2632.055
420	90	450	135	0.65	99.602	97.348	96.25	95.084	99.201	643.968	646.076	1535.429	1536.711	2624.849	2632.631
421	22.5	450	135	1.3	99.802	98.683	99.062	89.107	99.604	643.561	645.122	1535.744	1536.97	2625.676	2631.233
422	45	450	135	1.3	99.605	97.365	98.125	94.443	99.206	643.444	646.477	1534.815	1536.871	2623.112	2633.974
423	67.5	450	135	1.3	99.407	96.048	97.187	91.644	98.807	643.487	647.567	1533.944	1536.56	2621.162	2635.927
424	90	450	135	1.3	99.21	94.731	96.25	90.023	98.407	643.864	648.419	1533.368	1536.125	2620.382	2637.042
425	22.5	450	135	1.95	99.705	98.037	99.062	83.763	99.409	643.54	645.934	1535.043	1537.113	2624.496	2632.844
426	45	450	135	1.95	99.411	96.073	98.125	91.659	98.815	643.205	647.944	1533.401	1536.825	2620.277	2636.963
427	67.5	450	135	1.95	99.116	94.11	97.187	87.371	98.217	643.107	649.639	1532.028	1536.271	2616.958	2640.226
428	90	450	135	1.95	98.822	92.146	96.25	84.812	97.616	643.46	650.875	1530.932	1535.385	2615.224	2642.04
429	22.5	450	135	2.6	99.61	97.399	99.062	78.488	99.217	643.395	646.634	1533.797	1537.03	2622.989	2634.035
430	45	450	135	2.6	99.22	94.798	98.125	88.874	98.427	642.726	649.309	1531.245	1536.446	2616.874	2639.586

431	67.5	450	135	2.6	98.83	92.197	97.187	83.033	97.631	642.285	651.55	1529.059	1535.417	2611.68	2644.1
432	90	450	135	2.6	98.439	89.596	96.25	79.439	96.829	642.456	653.184	1527.308	1533.93	2608.611	2643.096

Table S2 Test cases and verification results.

S/N	RRP Frequencies (Hz)						RRP Frequency Ratios			Damage Properties				Damage Identification						
	f_{1A}	f_{1P}	f_{2A}	f_{2P}	f_{3A}	f_{3P}	f_{1A}/f_{1P}	f_{2A}/f_{2P}	f_{3A}/f_{3P}	α_d (°)	M_d (mm)	L_d (mm)	T_d (mm)	Dam. Det.?	RRP Freq. Rank	Loc. Band	Dam. Loc.?	$V_{predicted}$ ($\times V_0$)	V_{actual} ($\times V_0$)	Volume Prediction Accuracy
1	642.564	643.083	1535.289	1536.959	2627.133	2629.541	0.9992	0.9989	0.9991	74.32	92.65	70.51	0.69	Yes	$f_{1A}/f_{1P}>f_{3A}/f_{3P}>f_{2A}/f_{2P}$	3	Yes	0.9944	0.9982	99.690%
2	643.352	644.257	1537.863	1541.678	2629.687	2638.924	0.9986	0.9975	0.9965	82.57	193.04	119.77	1.38	Yes	$f_{1A}/f_{1P}>f_{2A}/f_{2P}>f_{3A}/f_{3P}$	2	Yes	0.9963	0.9932	99.741%
3	643.444	646.352	1536.102	1536.434	2623.555	2628.217	0.9955	0.9998	0.9982	57.78	382.56	82.69	1.76	Yes	$f_{2A}/f_{2P}>f_{3A}/f_{3P}>f_{1A}/f_{1P}$	5	Yes	0.9957	0.9958	99.991%
4	644.107	645.926	1532.979	1541.753	2617.353	2627.256	0.9972	0.9943	0.9962	66.99	315.14	76.01	2.22	Yes	$f_{1A}/f_{1P}>f_{3A}/f_{3P}>f_{2A}/f_{2P}$	3	Yes	0.9711	0.9944	98.068%
5	644.035	644.220	1535.945	1540.038	2628.464	2628.988	0.9997	0.9973	0.9998	43	247.65	60.67	1.55	Yes	$f_{3A}/f_{3P}>f_{1A}/f_{1P}>f_{2A}/f_{2P}$	1	Yes	0.6446	0.9980	70.700%
6	642.484	646.359	1534.616	1536.095	2618.585	2630.907	0.9940	0.9990	0.9953	76.39	403.47	47.55	2.43	Yes	$f_{2A}/f_{2P}>f_{3A}/f_{3P}>f_{1A}/f_{1P}$	5	Yes	0.9943	0.9956	99.890%
7	643.710	646.212	1533.455	1539.731	2618.486	2625.333	0.9961	0.9959	0.9974	75.1	336.04	67	2.2	Yes	$f_{3A}/f_{3P}>f_{1A}/f_{1P}>f_{2A}/f_{2P}$	1	No	-	0.9945	-
8	643.462	646.314	1535.422	1536.284	2622.841	2630.876	0.9956	0.9994	0.9969	75.44	400.69	84.2	1.37	Yes	$f_{2A}/f_{2P}>f_{3A}/f_{3P}>f_{1A}/f_{1P}$	5	Yes	0.9958	0.9956	99.987%
9	643.397	643.542	1537.061	1537.465	2629.068	2630.797	0.9998	0.9997	0.9993	26.54	153.34	129.82	0.9	Yes	$f_{1A}/f_{1P}>f_{2A}/f_{2P}>f_{3A}/f_{3P}$	2	Yes	1.0019	0.9984	-
10	643.506	647.206	1535.932	1536.179	2622.723	2628.299	0.9943	0.9998	0.9979	45.35	384.12	121.74	1.98	Yes	$f_{2A}/f_{2P}>f_{3A}/f_{3P}>f_{1A}/f_{1P}$	5	Yes	0.9945	0.9946	99.995%
11	643.390	646.605	1534.758	1536.902	2622.958	2633.247	0.9950	0.9986	0.9961	33.36	420.27	122.67	1.96	Yes	$f_{2A}/f_{2P}>f_{3A}/f_{3P}>f_{1A}/f_{1P}$	5	Yes	0.9953	0.9960	99.941%
12	643.462	643.498	1536.749	1537.518	2629.079	2630.058	0.9999	0.9995	0.9996	58.1	134.45	48.74	0.78	Yes	$f_{1A}/f_{1P}>f_{3A}/f_{3P}>f_{2A}/f_{2P}$	3	Yes	1.0032	0.9989	-
13	643.619	643.897	1537.041	1538.270	2628.421	2631.198	0.9996	0.9992	0.9989	44.99	199.63	87.51	0.72	Yes	$f_{1A}/f_{1P}>f_{2A}/f_{2P}>f_{3A}/f_{3P}$	2	Yes	1.0009	0.9986	-
14	639.497	640.354	1528.733	1537.579	2626.644	2629.630	0.9987	0.9942	0.9989	63.45	121.76	128.45	2.42	Yes	$f_{3A}/f_{3P}>f_{1A}/f_{1P}>f_{2A}/f_{2P}$	1	Yes	0.6431	0.9902	71.216%
15	642.803	643.105	1535.324	1537.617	2628.385	2629.880	0.9995	0.9985	0.9994	44.79	110.07	73.76	1.19	Yes	$f_{1A}/f_{1P}>f_{3A}/f_{3P}>f_{2A}/f_{2P}$	3	Yes	0.9984	0.9980	99.970%
16	643.337	643.877	1537.801	1537.982	2628.704	2634.069	0.9992	0.9999	0.9980	36.22	170.05	83.3	1.64	Yes	$f_{2A}/f_{2P}>f_{1A}/f_{1P}>f_{3A}/f_{3P}$	2	Yes	0.9990	0.9975	99.878%
17	642.200	642.964	1534.628	1536.826	2626.388	2629.613	0.9988	0.9986	0.9988	47.29	93	110.92	0.86	Yes	$f_{1A}/f_{1P}>f_{3A}/f_{3P}>f_{2A}/f_{2P}$	3	Yes	0.9900	0.9977	99.360%
18	644.294	644.432	1535.430	1541.062	2626.182	2628.561	0.9998	0.9963	0.9991	56.63	271.32	92.29	1.22	Yes	$f_{1A}/f_{1P}>f_{3A}/f_{3P}>f_{2A}/f_{2P}$	3	Yes	1.0014	0.9968	-
19	644.433	645.029	1536.081	1544.399	2628.809	2633.690	0.9991	0.9946	0.9981	84.9	234.49	103.87	1.53	Yes	$f_{1A}/f_{1P}>f_{3A}/f_{3P}>f_{2A}/f_{2P}$	3	Yes	0.9931	0.9933	99.986%
20	642.523	642.724	1534.877	1538.233	2629.989	2630.262	0.9997	0.9978	0.9999	69.64	121.69	50.83	1.83	Yes	$f_{3A}/f_{3P}>f_{1A}/f_{1P}>f_{2A}/f_{2P}$	1	Yes	0.6446	0.9968	70.798%
21	642.918	649.106	1533.184	1534.809	2615.339	2631.289	0.9905	0.9989	0.9939	88.98	398.39	94.21	2.28	Yes	$f_{2A}/f_{2P}>f_{3A}/f_{3P}>f_{1A}/f_{1P}$	5	Yes	0.9909	0.9905	99.969%
22	644.232	644.325	1535.711	1540.542	2626.735	2628.534	0.9999	0.9969	0.9993	62.16	269.09	84.74	1.06	Yes	$f_{1A}/f_{1P}>f_{3A}/f_{3P}>f_{2A}/f_{2P}$	3	Yes	1.0021	0.9972	-
23	642.011	642.483	1534.293	1537.718	2629.406	2630.391	0.9993	0.9978	0.9996	55.33	118.46	118.05	1.27	Yes	$f_{3A}/f_{3P}>f_{1A}/f_{1P}>f_{2A}/f_{2P}$	1	Yes	0.6440	0.9958	70.825%
24	643.117	643.145	1535.576	1537.979	2629.408	2629.666	1.0000	0.9984	0.9999	37.05	123.87	47.92	2.08	Yes	$f_{1A}/f_{1P}>f_{3A}/f_{3P}>f_{2A}/f_{2P}$	3	Yes	1.0033	0.9982	-
25	643.714	645.011	1536.102	1536.690	2625.896	2630.166	0.9980	0.9996	0.9984	84.48	408.53	76.5	0.7	Yes	$f_{2A}/f_{2P}>f_{3A}/f_{3P}>f_{1A}/f_{1P}$	5	Yes	0.9981	0.9977	99.969%

26	644.027	644.847	1534.788	1544.072	2627.941	2631.808	0.9987	0.9940	0.9985	45.24	236.76	104.44	2.38	Yes	$f_{1A}/f_{1P}>f_{3A}/f_{3P}>f_{2A}/f_{2P}$	3	Yes	0.9890	0.9944	99.550%
27	643.901	646.526	1535.427	1538.324	2624.173	2625.017	0.9959	0.9981	0.9997	64.67	357.78	103.19	1.53	Yes	$f_{3A}/f_{3P}>f_{2A}/f_{2P}>f_{1A}/f_{1P}$	4	Yes	0.9955	0.9949	99.950%
28	641.180	641.757	1533.326	1539.043	2628.930	2631.126	0.9991	0.9963	0.9992	71.97	141.07	93.39	2.54	Yes	$f_{3A}/f_{3P}>f_{1A}/f_{1P}>f_{2A}/f_{2P}$	1	Yes	0.6438	0.9916	71.164%
29	643.519	645.125	1535.325	1538.133	2623.502	2626.949	0.9975	0.9982	0.9987	36.53	346.74	55.71	2.54	Yes	$f_{3A}/f_{3P}>f_{2A}/f_{2P}>f_{1A}/f_{1P}$	4	Yes	0.9976	0.9974	99.987%
30	643.746	647.353	1535.646	1536.666	2622.589	2627.420	0.9944	0.9993	0.9982	72.04	378.68	109.44	1.58	Yes	$f_{2A}/f_{2P}>f_{3A}/f_{3P}>f_{1A}/f_{1P}$	5	Yes	0.9947	0.9938	99.925%
31	643.473	646.285	1535.041	1536.658	2622.966	2632.877	0.9956	0.9989	0.9962	69.15	417.7	94.92	1.23	Yes	$f_{2A}/f_{2P}>f_{3A}/f_{3P}>f_{1A}/f_{1P}$	5	Yes	0.9959	0.9960	99.995%
32	643.640	644.479	1536.422	1537.018	2626.920	2629.955	0.9987	0.9996	0.9988	27.14	420.57	95.46	0.83	Yes	$f_{2A}/f_{2P}>f_{3A}/f_{3P}>f_{1A}/f_{1P}$	5	Yes	0.9988	0.9989	99.990%
33	643.634	644.991	1536.557	1536.696	2626.044	2629.146	0.9979	0.9999	0.9988	67.59	390.77	59.39	1.03	Yes	$f_{2A}/f_{2P}>f_{3A}/f_{3P}>f_{1A}/f_{1P}$	5	Yes	0.9980	0.9979	99.994%
34	642.266	642.777	1534.752	1537.430	2628.381	2630.139	0.9992	0.9983	0.9993	61.57	109.75	102.1	0.96	Yes	$f_{3A}/f_{3P}>f_{1A}/f_{1P}>f_{2A}/f_{2P}$	1	Yes	0.6439	0.9970	70.724%
35	643.379	646.971	1534.282	1537.566	2621.919	2622.109	0.9944	0.9979	0.9999	76.89	361.47	74.69	2.17	Yes	$f_{3A}/f_{3P}>f_{2A}/f_{2P}>f_{1A}/f_{1P}$	4	Yes	0.9935	0.9938	99.974%
36	643.000	648.686	1533.165	1535.934	2617.199	2635.087	0.9912	0.9982	0.9932	76.12	411.13	101.07	2.07	Yes	$f_{2A}/f_{2P}>f_{3A}/f_{3P}>f_{1A}/f_{1P}$	5	Yes	0.9916	0.9921	99.959%
37	643.800	645.705	1535.561	1538.256	2624.404	2626.159	0.9971	0.9982	0.9993	54.19	352.04	101.41	1.38	Yes	$f_{3A}/f_{3P}>f_{2A}/f_{2P}>f_{1A}/f_{1P}$	4	Yes	0.9970	0.9962	99.934%
38	643.037	643.651	1537.387	1538.200	2628.962	2635.025	0.9990	0.9995	0.9977	42.37	159.32	85.55	1.86	Yes	$f_{2A}/f_{2P}>f_{1A}/f_{1P}>f_{3A}/f_{3P}$	2	Yes	0.9984	0.9966	99.854%
39	643.404	646.454	1534.740	1536.831	2622.710	2634.007	0.9953	0.9986	0.9957	55.23	431.06	112.61	1.3	Yes	$f_{2A}/f_{2P}>f_{3A}/f_{3P}>f_{1A}/f_{1P}$	5	Yes	0.9955	0.9960	99.962%
40	643.363	644.720	1536.457	1543.231	2627.978	2639.181	0.9979	0.9956	0.9958	55.21	206.32	114.85	2.2	Yes	$f_{1A}/f_{1P}>f_{3A}/f_{3P}>f_{2A}/f_{2P}$	3	Yes	0.9794	0.9931	98.866%
41	643.581	647.663	1535.571	1535.842	2621.078	2628.427	0.9937	0.9998	0.9972	78.91	385.25	97.85	1.76	Yes	$f_{2A}/f_{2P}>f_{3A}/f_{3P}>f_{1A}/f_{1P}$	5	Yes	0.9940	0.9932	99.936%
42	643.559	645.033	1536.070	1536.710	2625.195	2630.070	0.9977	0.9996	0.9981	84.91	405.93	45.71	1.17	Yes	$f_{2A}/f_{2P}>f_{3A}/f_{3P}>f_{1A}/f_{1P}$	5	Yes	0.9978	0.9977	99.994%
43	645.180	645.327	1534.531	1545.440	2625.775	2628.302	0.9998	0.9929	0.9990	82.65	265.51	109.2	1.67	Yes	$f_{1A}/f_{1P}>f_{3A}/f_{3P}>f_{2A}/f_{2P}$	3	Yes	1.0012	0.9925	-
44	642.375	642.657	1534.701	1537.930	2629.855	2630.302	0.9996	0.9979	0.9998	50.63	121.07	103.88	1.36	Yes	$f_{3A}/f_{3P}>f_{1A}/f_{1P}>f_{2A}/f_{2P}$	1	Yes	0.6444	0.9964	70.811%
45	643.700	645.790	1536.069	1537.448	2625.790	2625.878	0.9968	0.9991	1.0000	30.62	365.64	109.18	2.03	Yes	$f_{3A}/f_{3P}>f_{2A}/f_{2P}>f_{1A}/f_{1P}$	4	Yes	0.9966	0.9966	99.998%
46	643.093	643.417	1536.968	1538.090	2628.772	2633.856	0.9995	0.9993	0.9981	79.28	149.61	45.81	1.66	Yes	$f_{1A}/f_{1P}>f_{2A}/f_{2P}>f_{3A}/f_{3P}$	2	Yes	1.0006	0.9970	-
47	643.902	645.937	1536.391	1536.909	2625.428	2628.671	0.9968	0.9997	0.9988	70.33	382.06	128.71	0.84	Yes	$f_{2A}/f_{2P}>f_{3A}/f_{3P}>f_{1A}/f_{1P}$	5	No	-	0.9962	-
48	644.458	644.724	1535.100	1542.838	2628.250	2629.119	0.9996	0.9950	0.9997	77.02	249.81	73.68	1.65	Yes	$f_{3A}/f_{3P}>f_{1A}/f_{1P}>f_{2A}/f_{2P}$	1	Yes	0.6445	0.9953	70.905%
49	643.577	644.913	1536.154	1536.956	2626.094	2630.387	0.9979	0.9995	0.9984	25.24	412.76	98.92	1.34	Yes	$f_{2A}/f_{2P}>f_{3A}/f_{3P}>f_{1A}/f_{1P}$	5	Yes	0.9980	0.9983	99.973%
50	643.633	643.817	1537.129	1537.632	2628.313	2630.578	0.9997	0.9997	0.9991	42.15	184.3	46	0.87	Yes	$f_{1A}/f_{1P}>f_{2A}/f_{2P}>f_{3A}/f_{3P}$	2	Yes	1.0016	0.9992	-
51	643.979	645.642	1533.618	1541.013	2618.948	2627.694	0.9974	0.9952	0.9967	55.97	318.13	74.88	2.22	Yes	$f_{1A}/f_{1P}>f_{3A}/f_{3P}>f_{2A}/f_{2P}$	3	Yes	0.9739	0.9954	98.218%
52	644.451	645.600	1534.196	1541.771	2620.146	2627.624	0.9982	0.9951	0.9972	83.3	305.15	75.55	1.69	Yes	$f_{1A}/f_{1P}>f_{3A}/f_{3P}>f_{2A}/f_{2P}$	3	Yes	0.9831	0.9947	99.038%
53	643.796	645.906	1535.518	1538.451	2624.036	2626.149	0.9967	0.9981	0.9992	50.22	352.3	94.59	1.66	Yes	$f_{3A}/f_{3P}>f_{2A}/f_{2P}>f_{1A}/f_{1P}$	4	Yes	0.9966	0.9961	99.956%
54	643.498	644.695	1535.922	1542.856	2628.040	2635.362	0.9981	0.9955	0.9972	37.45	218.18	116.09	2.55	Yes	$f_{1A}/f_{1P}>f_{3A}/f_{3P}>f_{2A}/f_{2P}$	3	Yes	0.9822	0.9945	98.978%
55	642.342	642.747	1535.253	1537.254	2629.166	2630.072	0.9994	0.9987	0.9997	84.49	113.4	114.81	0.66	Yes	$f_{3A}/f_{3P}>f_{1A}/f_{1P}>f_{2A}/f_{2P}$	1	Yes	0.6441	0.9968	70.757%
56	643.355	647.945	1534.922	1536.492	2620.988	2624.876	0.9929	0.9990	0.9985	52.23	374.47	115.94	2.33	Yes	$f_{2A}/f_{2P}>f_{3A}/f_{3P}>f_{1A}/f_{1P}$	5	Yes	0.9932	0.9930	99.982%

57	643.497	645.722	1536.460	1536.478	2624.648	2629.024	0.9966	1.0000	0.9983	58.68	386.84	69.94	1.52	Yes	$f_{2A}/f_{2P}>f_{3A}/f_{3P}>f_{1A}/f_{1P}$	5	Yes	0.9967	0.9969	99.985%
58	644.313	644.764	1534.168	1543.550	2627.671	2629.611	0.9993	0.9939	0.9993	64.24	245.95	74.58	2.11	Yes	$f_{1A}/f_{1P}>f_{3A}/f_{3P}>f_{2A}/f_{2P}$	3	Yes	0.9957	0.9950	99.940%
59	644.462	644.640	1535.581	1541.917	2626.822	2628.609	0.9997	0.9959	0.9993	57.11	269.03	128.03	1.11	Yes	$f_{1A}/f_{1P}>f_{3A}/f_{3P}>f_{2A}/f_{2P}$	3	Yes	1.0006	0.9959	-
60	642.834	643.296	1536.311	1538.447	2629.237	2634.093	0.9993	0.9986	0.9982	47.1	146.7	76.76	1.98	Yes	$f_{1A}/f_{1P}>f_{2A}/f_{2P}>f_{3A}/f_{3P}$	2	Yes	0.9995	0.9964	99.744%
61	643.540	644.018	1537.368	1538.932	2628.548	2633.865	0.9993	0.9990	0.9980	64.5	189.73	74.91	1.05	Yes	$f_{1A}/f_{1P}>f_{2A}/f_{2P}>f_{3A}/f_{3P}$	2	Yes	0.9994	0.9975	99.839%
62	643.419	647.361	1534.492	1536.818	2621.683	2634.905	0.9939	0.9985	0.9950	52.61	424.48	126.74	1.57	Yes	$f_{2A}/f_{2P}>f_{3A}/f_{3P}>f_{1A}/f_{1P}$	5	Yes	0.9942	0.9948	99.952%
63	643.268	644.213	1537.375	1540.098	2629.087	2635.513	0.9985	0.9982	0.9976	30.74	194.99	134.23	2.09	Yes	$f_{1A}/f_{1P}>f_{2A}/f_{2P}>f_{3A}/f_{3P}$	2	Yes	0.9960	0.9957	99.977%
64	643.779	646.929	1535.830	1537.026	2623.680	2627.262	0.9951	0.9992	0.9986	65	375.9	113.5	1.47	Yes	$f_{2A}/f_{2P}>f_{3A}/f_{3P}>f_{1A}/f_{1P}$	5	Yes	0.9954	0.9946	99.935%
65	636.136	638.948	1522.439	1534.629	2617.138	2628.830	0.9956	0.9921	0.9956	86.32	97.64	94.82	2.59	Yes	$f_{1A}/f_{1P}>f_{3A}/f_{3P}>f_{2A}/f_{2P}$	3	Yes	0.9527	0.9895	96.943%
66	643.843	644.479	1535.949	1538.591	2625.221	2628.634	0.9990	0.9983	0.9987	33.48	321.67	55.36	1.72	Yes	$f_{1A}/f_{1P}>f_{3A}/f_{3P}>f_{2A}/f_{2P}$	3	Yes	0.9924	0.9984	99.502%
67	642.941	643.223	1535.680	1537.480	2628.288	2629.638	0.9996	0.9988	0.9995	57.54	106.94	50.44	1.07	Yes	$f_{1A}/f_{1P}>f_{3A}/f_{3P}>f_{2A}/f_{2P}$	3	Yes	0.9987	0.9984	99.975%
68	642.657	646.780	1533.554	1537.124	2618.868	2636.380	0.9936	0.9977	0.9934	54.65	443.36	69.13	2.29	Yes	$f_{2A}/f_{2P}>f_{1A}/f_{1P}>f_{3A}/f_{3P}$	2	No	-	0.9957	-
69	643.403	643.535	1536.926	1537.660	2629.137	2631.046	0.9998	0.9995	0.9993	62.75	144	55.61	0.85	Yes	$f_{1A}/f_{1P}>f_{2A}/f_{2P}>f_{3A}/f_{3P}$	2	Yes	1.0020	0.9985	-
70	643.071	643.575	1537.104	1538.169	2628.578	2634.483	0.9992	0.9993	0.9978	52.13	154.68	63.31	1.86	Yes	$f_{2A}/f_{2P}>f_{1A}/f_{1P}>f_{3A}/f_{3P}$	2	No	-	0.9969	-
71	644.392	644.543	1534.933	1542.183	2627.871	2628.401	0.9998	0.9953	0.9998	61.79	255.7	71.38	1.73	Yes	$f_{3A}/f_{3P}>f_{1A}/f_{1P}>f_{2A}/f_{2P}$	1	Yes	0.6447	0.9962	70.854%
72	643.648	643.827	1537.203	1537.768	2628.522	2630.819	0.9997	0.9996	0.9991	68.7	186	45.61	0.65	Yes	$f_{1A}/f_{1P}>f_{2A}/f_{2P}>f_{3A}/f_{3P}$	2	Yes	1.0016	0.9990	-
73	644.939	644.953	1534.250	1544.741	2626.319	2628.457	1.0000	0.9932	0.9992	67.4	262.14	100.89	1.83	Yes	$f_{1A}/f_{1P}>f_{3A}/f_{3P}>f_{2A}/f_{2P}$	3	Yes	1.0036	0.9938	-
74	643.517	644.709	1535.886	1542.573	2626.397	2638.822	0.9982	0.9957	0.9953	75.59	207.69	74.21	2.18	Yes	$f_{1A}/f_{1P}>f_{2A}/f_{2P}>f_{3A}/f_{3P}$	2	Yes	0.9942	0.9939	99.980%
75	644.571	644.986	1535.655	1544.223	2628.721	2631.304	0.9994	0.9945	0.9990	78.59	243.41	102.94	1.52	Yes	$f_{1A}/f_{1P}>f_{3A}/f_{3P}>f_{2A}/f_{2P}$	3	Yes	0.9963	0.9939	99.798%
76	644.410	646.398	1532.932	1542.940	2616.427	2626.748	0.9969	0.9935	0.9961	65.31	312.69	100.47	2.13	Yes	$f_{1A}/f_{1P}>f_{3A}/f_{3P}>f_{2A}/f_{2P}$	3	Yes	0.9681	0.9931	97.930%
77	643.057	645.540	1535.340	1537.026	2622.488	2632.275	0.9962	0.9989	0.9963	57.3	420.56	46.31	1.97	Yes	$f_{2A}/f_{2P}>f_{3A}/f_{3P}>f_{1A}/f_{1P}$	5	Yes	0.9963	0.9974	99.909%
78	643.633	643.741	1537.199	1537.206	2628.519	2629.748	0.9998	1.0000	0.9995	23.07	166.07	55.95	0.8	Yes	$f_{2A}/f_{2P}>f_{1A}/f_{1P}>f_{3A}/f_{3P}$	2	Yes	1.0021	0.9995	-
79	644.296	644.584	1535.487	1542.248	2628.537	2629.485	0.9996	0.9956	0.9996	56.32	247.73	87.68	1.57	Yes	$f_{3A}/f_{3P}>f_{1A}/f_{1P}>f_{2A}/f_{2P}$	1	Yes	0.6444	0.9961	70.835%
80	642.261	642.604	1533.458	1538.047	2627.357	2630.415	0.9995	0.9970	0.9988	35.41	111.67	72.72	2.34	Yes	$f_{1A}/f_{1P}>f_{3A}/f_{3P}>f_{2A}/f_{2P}$	3	Yes	0.9976	0.9970	99.950%
81	642.958	643.529	1537.556	1538.402	2629.684	2635.554	0.9991	0.9994	0.9978	49.54	158.1	106.5	1.57	Yes	$f_{2A}/f_{2P}>f_{1A}/f_{1P}>f_{3A}/f_{3P}$	2	Yes	0.9987	0.9959	99.765%
82	643.073	643.293	1535.895	1538.114	2629.041	2631.360	0.9997	0.9986	0.9991	36.84	136.65	55.25	2.18	Yes	$f_{1A}/f_{1P}>f_{3A}/f_{3P}>f_{2A}/f_{2P}$	3	Yes	0.9999	0.9978	99.829%
83	643.957	648.380	1535.277	1536.239	2620.887	2627.743	0.9932	0.9994	0.9974	82.92	381.67	123.61	1.62	Yes	$f_{2A}/f_{2P}>f_{3A}/f_{3P}>f_{1A}/f_{1P}$	5	No	-	0.9917	-
84	644.344	645.005	1535.445	1544.310	2628.174	2633.306	0.9990	0.9943	0.9981	81.29	235	90.16	1.76	Yes	$f_{1A}/f_{1P}>f_{3A}/f_{3P}>f_{2A}/f_{2P}$	3	Yes	0.9919	0.9936	99.861%
85	643.681	644.719	1536.714	1536.853	2626.877	2628.625	0.9984	0.9999	0.9993	54.11	383.25	73.54	0.81	Yes	$f_{2A}/f_{2P}>f_{3A}/f_{3P}>f_{1A}/f_{1P}$	5	Yes	0.9985	0.9984	99.990%
86	639.071	640.873	1527.046	1536.711	2620.563	2630.554	0.9972	0.9937	0.9962	52.66	99.83	101.49	2.57	Yes	$f_{1A}/f_{1P}>f_{3A}/f_{3P}>f_{2A}/f_{2P}$	3	Yes	0.9712	0.9932	98.171%
87	643.158	643.643	1538.338	1538.992	2630.493	2636.372	0.9992	0.9996	0.9978	87.41	170.31	121.32	0.94	Yes	$f_{2A}/f_{2P}>f_{1A}/f_{1P}>f_{3A}/f_{3P}$	2	Yes	0.9994	0.9950	99.635%

88	643.023	643.646	1538.431	1539.179	2630.316	2637.390	0.9990	0.9995	0.9973	83.88	170.39	115.73	1.17	Yes	$f_{2A}/f_{2P}>f_{1A}/f_{1P}>f_{3A}/f_{3P}$	2	Yes	0.9984	0.9943	99.661%
89	644.357	644.678	1534.791	1543.368	2627.087	2628.575	0.9995	0.9944	0.9994	35.77	255.59	110.01	2.38	Yes	$f_{1A}/f_{1P}>f_{3A}/f_{3P}>f_{2A}/f_{2P}$	3	Yes	0.9980	0.9954	99.781%
90	644.508	644.731	1534.955	1542.517	2623.655	2628.744	0.9997	0.9951	0.9981	30.17	277.54	121.66	2.31	Yes	$f_{1A}/f_{1P}>f_{3A}/f_{3P}>f_{2A}/f_{2P}$	3	Yes	0.9998	0.9958	99.668%
91	642.672	642.797	1534.644	1538.247	2629.647	2630.013	0.9998	0.9977	0.9999	43.08	121.39	63.52	2.09	Yes	$f_{3A}/f_{3P}>f_{1A}/f_{1P}>f_{2A}/f_{2P}$	1	Yes	0.6448	0.9972	70.783%
92	641.806	642.345	1533.236	1538.019	2628.367	2630.733	0.9992	0.9969	0.9991	45.26	114.79	103.89	1.84	Yes	$f_{1A}/f_{1P}>f_{3A}/f_{3P}>f_{2A}/f_{2P}$	3	Yes	0.9941	0.9957	99.866%
93	643.988	644.550	1535.661	1542.232	2628.512	2630.065	0.9991	0.9957	0.9994	30.35	240.32	113.54	2.31	Yes	$f_{3A}/f_{3P}>f_{1A}/f_{1P}>f_{2A}/f_{2P}$	1	Yes	0.6438	0.9961	70.791%
94	641.993	642.792	1534.910	1538.826	2629.092	2635.026	0.9988	0.9975	0.9977	49.28	146.7	92.95	2.57	Yes	$f_{1A}/f_{1P}>f_{3A}/f_{3P}>f_{2A}/f_{2P}$	3	Yes	0.9894	0.9942	99.601%
95	643.214	645.492	1535.307	1537.219	2623.158	2633.091	0.9965	0.9988	0.9962	53.54	438.52	52.79	1.73	Yes	$f_{2A}/f_{2P}>f_{1A}/f_{1P}>f_{3A}/f_{3P}$	2	No	-	0.9976	-
96	643.744	645.613	1535.776	1537.319	2625.282	2625.560	0.9971	0.9990	0.9999	84.74	364.36	63.17	1.39	Yes	$f_{3A}/f_{3P}>f_{2A}/f_{2P}>f_{1A}/f_{1P}$	4	Yes	0.9971	0.9963	99.932%
97	643.357	646.614	1534.419	1537.025	2621.722	2635.493	0.9950	0.9983	0.9948	72.7	448.17	81.55	1.46	Yes	$f_{2A}/f_{2P}>f_{1A}/f_{1P}>f_{3A}/f_{3P}$	2	No	-	0.9957	-
98	643.595	643.824	1537.057	1537.984	2628.475	2630.781	0.9996	0.9994	0.9991	29.51	194.57	112.67	0.75	Yes	$f_{1A}/f_{1P}>f_{2A}/f_{2P}>f_{3A}/f_{3P}$	2	Yes	1.0012	0.9987	-
99	643.782	645.049	1535.976	1538.042	2625.451	2627.172	0.9980	0.9987	0.9993	42.48	348.62	101.85	1.19	Yes	$f_{3A}/f_{3P}>f_{2A}/f_{2P}>f_{1A}/f_{1P}$	4	Yes	0.9983	0.9974	99.927%
100	643.588	644.577	1536.335	1537.049	2626.498	2630.334	0.9985	0.9995	0.9985	36.26	421.6	65.21	1	Yes	$f_{2A}/f_{2P}>f_{3A}/f_{3P}>f_{1A}/f_{1P}$	5	Yes	0.9986	0.9988	99.982%
101	644.928	645.485	1533.191	1545.127	2621.609	2628.060	0.9991	0.9923	0.9975	71.25	278.03	98.4	2.01	Yes	$f_{1A}/f_{1P}>f_{3A}/f_{3P}>f_{2A}/f_{2P}$	3	Yes	0.9938	0.9930	99.933%
102	643.620	648.456	1533.771	1536.459	2620.115	2636.732	0.9925	0.9983	0.9937	75.68	428.32	131.38	1.48	Yes	$f_{2A}/f_{2P}>f_{3A}/f_{3P}>f_{1A}/f_{1P}$	5	Yes	0.9929	0.9926	99.979%
103	643.938	646.660	1534.931	1538.364	2622.366	2624.197	0.9958	0.9978	0.9993	83.9	354.67	82.76	1.69	Yes	$f_{3A}/f_{3P}>f_{2A}/f_{2P}>f_{1A}/f_{1P}$	4	Yes	0.9953	0.9942	99.905%
104	643.677	643.976	1536.971	1538.535	2628.292	2631.447	0.9995	0.9990	0.9988	49.48	204.2	64.72	0.93	Yes	$f_{1A}/f_{1P}>f_{2A}/f_{2P}>f_{3A}/f_{3P}$	2	Yes	1.0007	0.9985	-
105	643.422	643.670	1537.124	1537.432	2628.692	2631.034	0.9996	0.9998	0.9991	23.93	159.67	84.96	1.25	Yes	$f_{2A}/f_{2P}>f_{1A}/f_{1P}>f_{3A}/f_{3P}$	2	Yes	1.0011	0.9987	-
106	643.842	644.283	1535.925	1540.294	2627.933	2630.948	0.9993	0.9972	0.9989	36.71	230.4	64.87	2.01	Yes	$f_{1A}/f_{1P}>f_{3A}/f_{3P}>f_{2A}/f_{2P}$	3	Yes	0.9959	0.9976	99.857%
107	643.940	647.519	1533.444	1540.078	2617.599	2623.280	0.9945	0.9957	0.9978	67.03	343.28	108.34	2.15	Yes	$f_{3A}/f_{3P}>f_{2A}/f_{2P}>f_{1A}/f_{1P}$	4	Yes	0.9936	0.9923	99.888%
108	643.664	645.742	1535.342	1536.942	2624.462	2633.038	0.9968	0.9990	0.9967	75.09	444.1	94.69	0.88	Yes	$f_{2A}/f_{2P}>f_{1A}/f_{1P}>f_{3A}/f_{3P}$	2	No	-	0.9969	-
109	643.506	645.445	1535.591	1537.028	2624.869	2632.168	0.9970	0.9991	0.9972	35.07	432.4	107.92	1.26	Yes	$f_{2A}/f_{2P}>f_{3A}/f_{3P}>f_{1A}/f_{1P}$	5	Yes	0.9972	0.9976	99.966%
110	644.368	644.445	1535.768	1541.661	2627.941	2628.851	0.9999	0.9962	0.9997	37.38	258.44	118.52	1.53	Yes	$f_{1A}/f_{1P}>f_{3A}/f_{3P}>f_{2A}/f_{2P}$	3	Yes	1.0024	0.9966	-
111	644.892	645.459	1532.988	1545.687	2620.309	2627.990	0.9991	0.9918	0.9971	47.98	279.25	122.25	2.48	Yes	$f_{1A}/f_{1P}>f_{3A}/f_{3P}>f_{2A}/f_{2P}$	3	Yes	0.9936	0.9928	99.934%
112	643.752	644.695	1535.893	1542.893	2628.260	2634.208	0.9985	0.9955	0.9977	40.17	223.49	114.86	2.23	Yes	$f_{1A}/f_{1P}>f_{3A}/f_{3P}>f_{2A}/f_{2P}$	3	Yes	0.9868	0.9949	99.329%
113	644.352	644.744	1535.302	1541.516	2623.615	2628.720	0.9994	0.9960	0.9981	27.09	286.44	123.05	2.15	Yes	$f_{1A}/f_{1P}>f_{3A}/f_{3P}>f_{2A}/f_{2P}$	3	Yes	0.9968	0.9964	99.970%
114	643.997	644.947	1535.479	1539.602	2623.327	2628.485	0.9985	0.9973	0.9980	36.49	319.61	83.12	1.79	Yes	$f_{1A}/f_{1P}>f_{3A}/f_{3P}>f_{2A}/f_{2P}$	3	Yes	0.9867	0.9973	99.121%
115	644.506	647.491	1534.816	1539.402	2621.244	2623.585	0.9954	0.9970	0.9991	89.86	348.84	119.47	1.48	Yes	$f_{3A}/f_{3P}>f_{2A}/f_{2P}>f_{1A}/f_{1P}$	4	Yes	0.9948	0.9921	99.773%
116	644.011	644.946	1536.163	1538.647	2625.702	2627.681	0.9986	0.9984	0.9992	61.46	333.84	124.77	0.69	Yes	$f_{3A}/f_{3P}>f_{1A}/f_{1P}>f_{2A}/f_{2P}$	1	Yes	0.6430	0.9973	70.617%
117	643.353	647.981	1534.495	1536.301	2620.499	2633.552	0.9929	0.9988	0.9950	58.56	408.05	123.7	1.78	Yes	$f_{2A}/f_{2P}>f_{3A}/f_{3P}>f_{1A}/f_{1P}$	5	Yes	0.9932	0.9936	99.969%
118	643.242	643.462	1537.031	1537.776	2629.330	2632.129	0.9997	0.9995	0.9989	46.8	150.51	103.67	0.93	Yes	$f_{1A}/f_{1P}>f_{2A}/f_{2P}>f_{3A}/f_{3P}$	2	Yes	1.0013	0.9977	-

119	643.294	644.235	1536.251	1539.381	2625.459	2635.794	0.9985	0.9980	0.9961	50.26	193.3	49.14	2.59	Yes	$f_{1A}/f_{1P}>f_{2A}/f_{2P}>f_{3A}/f_{3P}$	2	Yes	0.9960	0.9968	99.930%
120	643.566	645.338	1535.653	1537.038	2624.952	2632.304	0.9973	0.9991	0.9972	59.91	438.23	84.63	0.93	Yes	$f_{2A}/f_{2P}>f_{1A}/f_{1P}>f_{3A}/f_{3P}$	2	No	-	0.9976	-
121	643.290	643.669	1537.301	1537.912	2629.145	2632.914	0.9994	0.9996	0.9986	35.5	157.39	90.84	1.4	Yes	$f_{2A}/f_{2P}>f_{1A}/f_{1P}>f_{3A}/f_{3P}$	2	No	-	0.9977	-
122	643.356	643.557	1536.932	1537.528	2628.989	2631.013	0.9997	0.9996	0.9992	23.95	152.06	107.33	1.2	Yes	$f_{1A}/f_{1P}>f_{2A}/f_{2P}>f_{3A}/f_{3P}$	2	Yes	1.0015	0.9985	-
123	643.091	644.272	1537.523	1540.778	2627.502	2640.305	0.9982	0.9979	0.9952	76.77	186.23	81.29	2	Yes	$f_{1A}/f_{1P}>f_{2A}/f_{2P}>f_{3A}/f_{3P}$	2	Yes	0.9943	0.9938	99.958%
124	643.615	644.371	1536.439	1537.069	2626.925	2630.070	0.9988	0.9996	0.9988	28.53	430.37	64.3	0.96	Yes	$f_{2A}/f_{2P}>f_{1A}/f_{1P}>f_{3A}/f_{3P}$	2	No	-	0.9991	-
125	643.213	644.365	1536.653	1540.589	2625.880	2639.239	0.9982	0.9974	0.9949	77.91	191.78	60.99	2.24	Yes	$f_{1A}/f_{1P}>f_{2A}/f_{2P}>f_{3A}/f_{3P}$	2	Yes	0.9945	0.9947	99.982%
126	642.829	643.195	1536.883	1538.415	2629.867	2634.715	0.9994	0.9990	0.9982	67.46	148.74	91.3	1.37	Yes	$f_{1A}/f_{1P}>f_{2A}/f_{2P}>f_{3A}/f_{3P}$	2	Yes	1.0002	0.9958	-
127	643.944	644.162	1536.296	1538.674	2626.595	2628.709	0.9997	0.9985	0.9992	40.65	293.41	71.99	0.89	Yes	$f_{1A}/f_{1P}>f_{3A}/f_{3P}>f_{2A}/f_{2P}$	3	Yes	0.9999	0.9987	99.900%
128	644.345	645.651	1534.902	1541.447	2621.892	2627.923	0.9980	0.9958	0.9977	49.02	312.34	121.6	1.58	Yes	$f_{1A}/f_{1P}>f_{3A}/f_{3P}>f_{2A}/f_{2P}$	3	Yes	0.9803	0.9953	98.756%
129	643.990	644.222	1536.624	1540.215	2628.791	2630.503	0.9996	0.9977	0.9993	47.61	234.74	134.9	0.83	Yes	$f_{1A}/f_{1P}>f_{3A}/f_{3P}>f_{2A}/f_{2P}$	3	Yes	0.9996	0.9973	99.811%
130	643.367	645.776	1536.086	1536.236	2623.433	2628.239	0.9963	0.9999	0.9982	83.4	385.55	49.66	1.76	Yes	$f_{2A}/f_{2P}>f_{3A}/f_{3P}>f_{1A}/f_{1P}$	5	Yes	0.9965	0.9964	99.989%
131	641.998	642.404	1534.055	1538.827	2629.819	2633.270	0.9994	0.9969	0.9987	49.99	137.72	93.32	2.41	Yes	$f_{1A}/f_{1P}>f_{3A}/f_{3P}>f_{2A}/f_{2P}$	3	Yes	0.9965	0.9944	99.830%
132	640.306	641.694	1531.013	1537.184	2624.692	2630.870	0.9978	0.9960	0.9977	70.65	100.58	85.14	1.84	Yes	$f_{1A}/f_{1P}>f_{3A}/f_{3P}>f_{2A}/f_{2P}$	3	Yes	0.9787	0.9945	98.689%
133	643.446	644.768	1536.051	1537.018	2625.485	2630.837	0.9980	0.9994	0.9980	48.22	423.64	48.62	1.28	Yes	$f_{2A}/f_{2P}>f_{3A}/f_{3P}>f_{1A}/f_{1P}$	5	Yes	0.9981	0.9985	99.967%
134	643.421	644.308	1537.569	1541.088	2629.242	2637.485	0.9986	0.9977	0.9969	55.5	194.33	125.75	1.52	Yes	$f_{1A}/f_{1P}>f_{2A}/f_{2P}>f_{3A}/f_{3P}$	2	Yes	0.9964	0.9947	99.859%
135	643.419	648.154	1534.402	1535.745	2619.187	2631.941	0.9927	0.9991	0.9952	85.03	400.04	96.58	1.84	Yes	$f_{2A}/f_{2P}>f_{3A}/f_{3P}>f_{1A}/f_{1P}$	5	Yes	0.9930	0.9925	99.957%
136	642.297	648.382	1531.916	1536.582	2615.072	2638.655	0.9906	0.9970	0.9911	65.24	430.63	86.34	2.47	Yes	$f_{2A}/f_{2P}>f_{3A}/f_{3P}>f_{1A}/f_{1P}$	5	Yes	0.9910	0.9931	99.824%
137	643.711	645.891	1532.991	1540.288	2617.842	2626.401	0.9966	0.9953	0.9967	70.45	327.32	61.33	2.37	Yes	$f_{3A}/f_{3P}>f_{1A}/f_{1P}>f_{2A}/f_{2P}$	1	Yes	0.6402	0.9949	70.589%
138	643.607	646.445	1536.116	1536.239	2624.335	2627.341	0.9956	0.9999	0.9989	25.95	382.76	131.21	2.48	Yes	$f_{2A}/f_{2P}>f_{3A}/f_{3P}>f_{1A}/f_{1P}$	5	Yes	0.9958	0.9958	99.998%
139	642.068	642.427	1536.502	1538.818	2630.720	2635.293	0.9994	0.9985	0.9983	70.81	148.55	134.06	1.68	Yes	$f_{1A}/f_{1P}>f_{2A}/f_{2P}>f_{3A}/f_{3P}$	2	Yes	1.0003	0.9920	-
140	643.196	643.744	1537.384	1537.949	2628.604	2633.990	0.9991	0.9996	0.9980	36.12	161.22	73.3	1.94	Yes	$f_{2A}/f_{2P}>f_{1A}/f_{1P}>f_{3A}/f_{3P}$	2	Yes	0.9989	0.9974	99.879%
141	643.221	643.648	1537.705	1537.873	2629.300	2633.350	0.9993	0.9999	0.9985	36.28	168.03	133.48	1.22	Yes	$f_{2A}/f_{2P}>f_{1A}/f_{1P}>f_{3A}/f_{3P}$	2	Yes	0.9998	0.9970	99.771%
142	639.022	641.226	1529.173	1535.631	2621.632	2630.166	0.9966	0.9958	0.9968	69.14	92.48	117.96	1.67	Yes	$f_{3A}/f_{3P}>f_{1A}/f_{1P}>f_{2A}/f_{2P}$	1	Yes	0.6401	0.9932	70.721%
143	643.558	643.751	1537.424	1537.562	2628.827	2631.275	0.9997	0.9999	0.9991	80.01	169.06	46.23	0.69	Yes	$f_{2A}/f_{2P}>f_{1A}/f_{1P}>f_{3A}/f_{3P}$	2	Yes	1.0015	0.9987	-
144	643.446	643.922	1537.268	1537.769	2627.666	2632.587	0.9993	0.9997	0.9981	29.18	176.89	47.2	2.28	Yes	$f_{2A}/f_{2P}>f_{1A}/f_{1P}>f_{3A}/f_{3P}$	2	Yes	0.9994	0.9984	99.921%
145	643.420	643.883	1537.385	1538.524	2628.922	2632.898	0.9993	0.9993	0.9985	30.18	185.22	132.5	1.26	Yes	$f_{1A}/f_{1P}>f_{2A}/f_{2P}>f_{3A}/f_{3P}$	2	Yes	0.9995	0.9975	99.832%
146	643.595	645.776	1535.890	1536.831	2625.100	2630.325	0.9966	0.9994	0.9980	24.89	401.69	123	1.91	Yes	$f_{2A}/f_{2P}>f_{3A}/f_{3P}>f_{1A}/f_{1P}$	5	Yes	0.9968	0.9971	99.976%
147	644.195	645.111	1535.411	1540.473	2621.716	2628.146	0.9986	0.9967	0.9976	22.94	311.6	130.14	2.41	Yes	$f_{1A}/f_{1P}>f_{3A}/f_{3P}>f_{2A}/f_{2P}$	3	Yes	0.9873	0.9964	99.242%
148	643.372	644.450	1536.682	1541.018	2628.420	2634.646	0.9983	0.9972	0.9976	27.88	210.39	116.99	2.57	Yes	$f_{1A}/f_{1P}>f_{3A}/f_{3P}>f_{2A}/f_{2P}$	3	Yes	0.9844	0.9959	99.050%
149	643.665	644.189	1536.924	1539.497	2627.927	2633.826	0.9992	0.9983	0.9978	64.67	201.31	56.11	1.42	Yes	$f_{1A}/f_{1P}>f_{2A}/f_{2P}>f_{3A}/f_{3P}$	2	Yes	0.9991	0.9974	99.861%

150	644.525	645.803	1533.008	1543.432	2618.274	2627.795	0.9980	0.9932	0.9964	68.16	298.76	89.31	2.07	Yes	$f_{1A}/f_{1P}>f_{3A}/f_{3P}>f_{2A}/f_{2P}$	3	Yes	0.9808	0.9937	98.927%
151	642.525	642.797	1536.428	1538.490	2630.338	2634.616	0.9996	0.9987	0.9984	85.12	143.79	88.66	1.38	Yes	$f_{1A}/f_{1P}>f_{2A}/f_{2P}>f_{3A}/f_{3P}$	2	Yes	1.0009	0.9948	-
152	643.694	644.778	1536.631	1536.848	2626.684	2628.346	0.9983	0.9999	0.9994	70.51	380.84	57.3	0.9	Yes	$f_{2A}/f_{2P}>f_{3A}/f_{3P}>f_{1A}/f_{1P}$	5	No	-	0.9982	-
153	643.385	645.534	1535.499	1537.180	2624.091	2632.608	0.9967	0.9989	0.9968	42.31	426.6	71.87	1.61	Yes	$f_{2A}/f_{2P}>f_{3A}/f_{3P}>f_{1A}/f_{1P}$	5	Yes	0.9968	0.9976	99.937%
154	643.937	647.697	1535.176	1535.875	2621.724	2631.293	0.9942	0.9995	0.9964	88.08	399.38	124.81	1.28	Yes	$f_{2A}/f_{2P}>f_{3A}/f_{3P}>f_{1A}/f_{1P}$	5	Yes	0.9945	0.9930	99.872%
155	643.760	644.553	1536.540	1537.466	2627.348	2627.635	0.9988	0.9994	0.9999	35.88	357.52	115.65	0.73	Yes	$f_{3A}/f_{3P}>f_{2A}/f_{2P}>f_{1A}/f_{1P}$	4	Yes	0.9993	0.9985	99.932%
156	642.849	647.151	1533.768	1536.805	2619.474	2635.179	0.9934	0.9980	0.9940	53.11	421.65	84.98	2.19	Yes	$f_{2A}/f_{2P}>f_{3A}/f_{3P}>f_{1A}/f_{1P}$	5	Yes	0.9937	0.9951	99.884%
157	643.986	644.701	1536.475	1543.377	2628.938	2635.216	0.9989	0.9955	0.9976	71.98	222.34	126.05	1.39	Yes	$f_{1A}/f_{1P}>f_{3A}/f_{3P}>f_{2A}/f_{2P}$	3	Yes	0.9909	0.9937	99.768%
158	643.785	645.159	1534.653	1539.361	2622.220	2627.774	0.9979	0.9969	0.9979	62.24	327.29	52.15	1.96	Yes	$f_{3A}/f_{3P}>f_{1A}/f_{1P}>f_{2A}/f_{2P}$	1	Yes	0.6420	0.9968	70.578%
159	642.103	642.771	1533.682	1537.701	2626.136	2630.365	0.9990	0.9974	0.9984	41.59	101.57	56.89	2.16	Yes	$f_{1A}/f_{1P}>f_{3A}/f_{3P}>f_{2A}/f_{2P}$	3	Yes	0.9918	0.9975	99.527%
160	642.701	643.611	1538.611	1539.304	2630.020	2638.560	0.9986	0.9995	0.9968	67.29	169.3	124.75	1.69	Yes	$f_{2A}/f_{2P}>f_{1A}/f_{1P}>f_{3A}/f_{3P}$	2	Yes	0.9962	0.9929	99.729%
161	643.093	643.107	1535.997	1537.906	2629.469	2630.664	1.0000	0.9988	0.9995	65.3	128.54	48.82	1.38	Yes	$f_{1A}/f_{1P}>f_{3A}/f_{3P}>f_{2A}/f_{2P}$	3	Yes	1.0036	0.9978	-
162	643.790	645.131	1536.182	1537.811	2626.207	2626.996	0.9979	0.9989	0.9997	29.47	355.35	129.55	1.34	Yes	$f_{3A}/f_{3P}>f_{2A}/f_{2P}>f_{1A}/f_{1P}$	4	Yes	0.9982	0.9974	99.937%
163	643.819	644.847	1536.407	1537.267	2626.880	2626.974	0.9984	0.9994	1.0000	83.54	364.43	59.92	0.88	Yes	$f_{3A}/f_{3P}>f_{2A}/f_{2P}>f_{1A}/f_{1P}$	4	Yes	0.9988	0.9978	99.916%
164	642.965	643.088	1535.182	1537.877	2628.755	2629.903	0.9998	0.9982	0.9996	28.64	116.71	72.03	1.91	Yes	$f_{1A}/f_{1P}>f_{3A}/f_{3P}>f_{2A}/f_{2P}$	3	Yes	1.0016	0.9980	-
165	644.081	646.126	1535.236	1539.208	2622.987	2625.950	0.9968	0.9974	0.9989	68.36	343.04	115	1.27	Yes	$f_{3A}/f_{3P}>f_{2A}/f_{2P}>f_{1A}/f_{1P}$	4	Yes	0.9967	0.9950	99.859%
166	643.202	643.311	1536.161	1537.406	2629.105	2629.323	0.9998	0.9992	0.9999	39.47	119.63	95.6	0.72	Yes	$f_{3A}/f_{3P}>f_{1A}/f_{1P}>f_{2A}/f_{2P}$	1	Yes	0.6448	0.9986	70.661%
167	643.342	643.657	1537.487	1537.806	2629.058	2632.934	0.9995	0.9998	0.9985	60.86	159.01	71.14	1.01	Yes	$f_{2A}/f_{2P}>f_{1A}/f_{1P}>f_{3A}/f_{3P}$	2	Yes	1.0006	0.9978	-
168	642.878	642.926	1536.257	1538.050	2630.172	2632.356	0.9999	0.9988	0.9992	78.51	135.75	88.81	0.99	Yes	$f_{1A}/f_{1P}>f_{3A}/f_{3P}>f_{2A}/f_{2P}$	3	Yes	1.0030	0.9965	-
169	644.500	644.815	1534.967	1544.102	2628.395	2628.691	0.9995	0.9941	0.9999	42.63	252.64	126.12	2.05	Yes	$f_{3A}/f_{3P}>f_{1A}/f_{1P}>f_{2A}/f_{2P}$	1	Yes	0.6443	0.9945	70.963%
170	640.993	641.733	1531.284	1538.258	2627.019	2630.557	0.9988	0.9955	0.9987	61.62	112.87	68.47	2.47	Yes	$f_{1A}/f_{1P}>f_{3A}/f_{3P}>f_{2A}/f_{2P}$	3	Yes	0.9904	0.9948	99.634%
171	643.947	644.845	1536.347	1538.049	2626.071	2627.574	0.9986	0.9989	0.9994	73.48	345.22	85.99	0.72	Yes	$f_{3A}/f_{3P}>f_{2A}/f_{2P}>f_{1A}/f_{1P}$	4	Yes	0.9991	0.9977	99.885%
172	643.473	645.715	1535.299	1536.643	2623.354	2631.778	0.9965	0.9991	0.9968	88.82	416.6	56.84	1.36	Yes	$f_{2A}/f_{2P}>f_{3A}/f_{3P}>f_{1A}/f_{1P}$	5	Yes	0.9967	0.9966	99.989%
173	644.556	645.304	1534.665	1542.845	2622.661	2628.472	0.9988	0.9947	0.9978	45.11	290.4	126.01	1.77	Yes	$f_{1A}/f_{1P}>f_{3A}/f_{3P}>f_{2A}/f_{2P}$	3	Yes	0.9904	0.9950	99.620%
174	642.793	642.804	1536.177	1538.083	2630.297	2632.515	1.0000	0.9988	0.9992	62.73	137.71	122.87	1.04	Yes	$f_{1A}/f_{1P}>f_{3A}/f_{3P}>f_{2A}/f_{2P}$	3	Yes	1.0036	0.9960	-
175	643.598	644.817	1536.383	1536.895	2626.275	2629.904	0.9981	0.9997	0.9986	43.05	403.44	76.99	0.98	Yes	$f_{2A}/f_{2P}>f_{3A}/f_{3P}>f_{1A}/f_{1P}$	5	Yes	0.9982	0.9984	99.986%
176	643.233	643.710	1537.289	1537.916	2628.820	2633.428	0.9993	0.9996	0.9983	32.93	159.55	78.95	1.82	Yes	$f_{2A}/f_{2P}>f_{1A}/f_{1P}>f_{3A}/f_{3P}$	2	Yes	0.9994	0.9976	99.854%
177	644.334	646.244	1535.143	1539.770	2621.833	2625.771	0.9970	0.9970	0.9985	85.35	336.14	108.84	1.23	Yes	$f_{3A}/f_{3P}>f_{1A}/f_{1P}>f_{2A}/f_{2P}$	1	No	-	0.9943	-
178	643.692	648.895	1532.895	1536.267	2619.088	2638.755	0.9920	0.9978	0.9925	88.07	448.84	124.01	1.54	Yes	$f_{2A}/f_{2P}>f_{3A}/f_{3P}>f_{1A}/f_{1P}$	5	Yes	0.9923	0.9916	99.942%
179	643.686	647.812	1532.980	1539.278	2616.724	2621.579	0.9936	0.9959	0.9981	74.69	348.6	96.42	2.33	Yes	$f_{3A}/f_{3P}>f_{2A}/f_{2P}>f_{1A}/f_{1P}$	4	Yes	0.9924	0.9917	99.941%
180	643.805	644.311	1536.421	1537.937	2626.302	2628.383	0.9992	0.9990	0.9992	24.4	331.91	73.61	1.33	Yes	$f_{1A}/f_{1P}>f_{3A}/f_{3P}>f_{2A}/f_{2P}$	3	Yes	0.9947	0.9988	99.660%

181	643.599	645.612	1536.450	1536.479	2625.273	2629.104	0.9969	1.0000	0.9985	44.11	388.34	113.39	1.2	Yes	$f_{2A}/f_{2P}>f_{3A}/f_{3P}>f_{1A}/f_{1P}$	5	Yes	0.9970	0.9970	99.999%
182	643.427	643.935	1537.586	1538.967	2628.956	2634.610	0.9992	0.9991	0.9979	63.98	183.67	99.12	0.98	Yes	$f_{1A}/f_{1P}>f_{2A}/f_{2P}>f_{3A}/f_{3P}$	2	Yes	0.9992	0.9969	99.808%
183	643.959	644.132	1536.330	1539.508	2628.596	2628.976	0.9997	0.9979	0.9999	28.1	246.38	74.56	1.56	Yes	$f_{3A}/f_{3P}>f_{1A}/f_{1P}>f_{2A}/f_{2P}$	1	Yes	0.6447	0.9984	70.670%
184	643.756	644.208	1536.687	1539.671	2628.279	2632.023	0.9993	0.9981	0.9986	37.1	216.63	79.65	1.48	Yes	$f_{1A}/f_{1P}>f_{3A}/f_{3P}>f_{2A}/f_{2P}$	3	Yes	0.9957	0.9978	99.825%
185	643.929	644.800	1534.447	1539.986	2622.598	2628.659	0.9986	0.9964	0.9977	56.79	307.88	48.75	2.04	Yes	$f_{1A}/f_{1P}>f_{3A}/f_{3P}>f_{2A}/f_{2P}$	3	Yes	0.9881	0.9972	99.246%
186	642.236	643.693	1538.185	1538.193	2627.400	2638.740	0.9977	1.0000	0.9957	54.94	169.12	95.69	2.59	Yes	$f_{2A}/f_{2P}>f_{1A}/f_{1P}>f_{3A}/f_{3P}$	2	Yes	0.9922	0.9933	99.911%
187	643.847	644.642	1536.665	1542.938	2628.915	2635.981	0.9988	0.9959	0.9973	68.38	216.7	122.6	1.43	Yes	$f_{1A}/f_{1P}>f_{3A}/f_{3P}>f_{2A}/f_{2P}$	3	Yes	0.9895	0.9940	99.626%
188	642.771	643.851	1538.083	1539.047	2629.448	2637.614	0.9983	0.9994	0.9969	41.4	175.41	130.44	2.11	Yes	$f_{2A}/f_{2P}>f_{1A}/f_{1P}>f_{3A}/f_{3P}$	2	Yes	0.9950	0.9943	99.946%
189	643.065	643.227	1536.248	1537.936	2629.663	2631.525	0.9997	0.9989	0.9993	33.92	139.84	110.49	1.38	Yes	$f_{1A}/f_{1P}>f_{3A}/f_{3P}>f_{2A}/f_{2P}$	3	Yes	1.0009	0.9974	-
190	643.102	643.380	1536.612	1538.223	2629.625	2632.831	0.9996	0.9990	0.9988	44.4	144.4	85.8	1.46	Yes	$f_{1A}/f_{1P}>f_{2A}/f_{2P}>f_{3A}/f_{3P}$	2	Yes	1.0009	0.9972	-
191	643.669	644.780	1537.723	1544.177	2629.953	2640.478	0.9983	0.9958	0.9960	87.72	204.28	125.35	1.63	Yes	$f_{1A}/f_{1P}>f_{3A}/f_{3P}>f_{2A}/f_{2P}$	3	Yes	0.9838	0.9911	99.398%
192	643.600	644.979	1536.298	1537.403	2626.757	2626.887	0.9979	0.9993	1.0000	47.4	364.91	53.01	1.62	Yes	$f_{3A}/f_{3P}>f_{2A}/f_{2P}>f_{1A}/f_{1P}$	4	Yes	0.9981	0.9980	99.989%
193	643.779	644.482	1536.674	1541.069	2627.894	2635.529	0.9989	0.9971	0.9971	84.29	209.78	61.72	1.62	Yes	$f_{1A}/f_{1P}>f_{2A}/f_{2P}>f_{3A}/f_{3P}$	2	Yes	0.9978	0.9958	99.836%
194	644.406	644.659	1535.003	1542.969	2628.498	2628.524	0.9996	0.9948	1.0000	52.66	252.48	93.71	1.79	Yes	$f_{3A}/f_{3P}>f_{1A}/f_{1P}>f_{2A}/f_{2P}$	1	Yes	0.6445	0.9956	70.885%
195	643.798	646.011	1535.421	1536.737	2624.492	2632.642	0.9966	0.9991	0.9969	81.89	429.24	116.41	0.78	Yes	$f_{2A}/f_{2P}>f_{3A}/f_{3P}>f_{1A}/f_{1P}$	5	Yes	0.9967	0.9963	99.964%
196	643.650	646.308	1536.039	1536.706	2624.337	2627.989	0.9959	0.9996	0.9986	54.34	379.86	117.89	1.35	Yes	$f_{2A}/f_{2P}>f_{3A}/f_{3P}>f_{1A}/f_{1P}$	5	Yes	0.9961	0.9957	99.966%
197	643.621	643.762	1537.229	1537.358	2628.499	2630.222	0.9998	0.9999	0.9993	35.66	170.85	52.77	0.74	Yes	$f_{2A}/f_{2P}>f_{1A}/f_{1P}>f_{3A}/f_{3P}$	2	Yes	1.0019	0.9993	-
198	644.395	645.572	1534.767	1541.579	2621.589	2627.931	0.9982	0.9956	0.9976	62.79	308.4	107.66	1.44	Yes	$f_{1A}/f_{1P}>f_{3A}/f_{3P}>f_{2A}/f_{2P}$	3	Yes	0.9826	0.9951	98.961%
199	643.907	644.782	1535.011	1543.545	2627.938	2631.938	0.9986	0.9945	0.9985	40.11	234.54	103.06	2.51	Yes	$f_{1A}/f_{1P}>f_{3A}/f_{3P}>f_{2A}/f_{2P}$	3	Yes	0.9881	0.9949	99.439%
200	644.115	645.979	1534.868	1540.242	2621.452	2626.978	0.9971	0.9965	0.9979	56.57	331	104.34	1.62	Yes	$f_{3A}/f_{3P}>f_{1A}/f_{1P}>f_{2A}/f_{2P}$	1	Yes	0.6409	0.9952	70.622%
201	643.731	646.197	1535.548	1538.008	2623.931	2625.419	0.9962	0.9984	0.9994	35.29	358.27	114.52	2.13	Yes	$f_{3A}/f_{3P}>f_{2A}/f_{2P}>f_{1A}/f_{1P}$	4	Yes	0.9958	0.9957	99.994%
202	644.259	644.475	1534.378	1541.929	2627.897	2627.975	0.9997	0.9951	1.0000	58.96	253.28	58.13	2.08	Yes	$f_{3A}/f_{3P}>f_{1A}/f_{1P}>f_{2A}/f_{2P}$	1	Yes	0.6446	0.9965	70.821%
203	644.050	644.067	1536.096	1539.313	2627.205	2628.773	1.0000	0.9979	0.9994	24.61	266.95	63.05	1.89	Yes	$f_{1A}/f_{1P}>f_{3A}/f_{3P}>f_{2A}/f_{2P}$	3	Yes	1.0035	0.9985	-
204	642.331	643.369	1537.855	1538.578	2629.557	2637.724	0.9984	0.9995	0.9969	52.66	163.36	122.67	2.14	Yes	$f_{2A}/f_{2P}>f_{1A}/f_{1P}>f_{3A}/f_{3P}$	2	Yes	0.9953	0.9931	99.821%
205	643.623	644.558	1536.387	1541.804	2628.203	2634.986	0.9985	0.9965	0.9974	39.93	214.89	106.73	2.09	Yes	$f_{1A}/f_{1P}>f_{3A}/f_{3P}>f_{2A}/f_{2P}$	3	Yes	0.9870	0.9956	99.289%
206	643.842	646.792	1535.499	1537.461	2623.949	2625.954	0.9954	0.9987	0.9992	66.48	368.5	125.4	1.34	Yes	$f_{3A}/f_{3P}>f_{2A}/f_{2P}>f_{1A}/f_{1P}$	4	Yes	0.9949	0.9944	99.960%
207	643.066	643.249	1535.980	1537.544	2629.051	2629.581	0.9997	0.9990	0.9998	67.46	114.06	49.05	0.96	Yes	$f_{3A}/f_{3P}>f_{1A}/f_{1P}>f_{2A}/f_{2P}$	1	Yes	0.6446	0.9984	70.663%
208	644.413	644.552	1535.946	1541.265	2627.016	2628.614	0.9998	0.9965	0.9994	69.06	269.4	120.3	0.88	Yes	$f_{1A}/f_{1P}>f_{3A}/f_{3P}>f_{2A}/f_{2P}$	3	Yes	1.0013	0.9963	-
209	643.455	648.000	1532.138	1538.477	2614.820	2619.287	0.9930	0.9959	0.9983	89.17	350.93	83.64	2.51	Yes	$f_{3A}/f_{3P}>f_{2A}/f_{2P}>f_{1A}/f_{1P}$	4	Yes	0.9916	0.9907	99.929%
210	643.359	645.206	1535.591	1536.966	2624.231	2631.801	0.9971	0.9991	0.9971	61.48	425.99	53.77	1.35	Yes	$f_{2A}/f_{2P}>f_{1A}/f_{1P}>f_{3A}/f_{3P}$	2	No	-	0.9978	-
211	644.110	644.477	1536.121	1539.604	2626.207	2628.574	0.9994	0.9977	0.9991	47.32	293.45	125.42	0.77	Yes	$f_{1A}/f_{1P}>f_{3A}/f_{3P}>f_{2A}/f_{2P}$	3	Yes	0.9972	0.9977	99.958%

212	640.383	641.757	1531.397	1537.015	2624.763	2630.774	0.9979	0.9963	0.9977	76.42	98.83	81.55	1.71	Yes	$f_{1A}/f_{1P}>f_{3A}/f_{3P}>f_{2A}/f_{2P}$	3	Yes	0.9789	0.9947	98.693%
213	644.095	644.217	1535.785	1539.690	2626.253	2628.900	0.9998	0.9975	0.9990	31.39	277.35	62.62	1.81	Yes	$f_{1A}/f_{1P}>f_{3A}/f_{3P}>f_{2A}/f_{2P}$	3	Yes	1.0016	0.9982	-
214	643.127	647.881	1534.420	1536.140	2619.871	2632.479	0.9927	0.9989	0.9952	55.96	402.7	109	2.07	Yes	$f_{2A}/f_{2P}>f_{3A}/f_{3P}>f_{1A}/f_{1P}$	5	Yes	0.9930	0.9937	99.940%
215	644.228	644.903	1535.874	1544.245	2628.924	2633.442	0.9990	0.9946	0.9983	56.41	232.8	130.69	1.7	Yes	$f_{1A}/f_{1P}>f_{3A}/f_{3P}>f_{2A}/f_{2P}$	3	Yes	0.9917	0.9938	99.830%
216	639.841	641.641	1531.255	1535.377	2623.447	2629.460	0.9972	0.9973	0.9977	78.59	90.44	130.29	1.12	Yes	$f_{3A}/f_{3P}>f_{2A}/f_{2P}>f_{1A}/f_{1P}$	4	No	-	0.9943	-
217	643.726	643.956	1536.749	1538.432	2628.193	2630.439	0.9996	0.9989	0.9991	36.54	216.08	56.61	1.12	Yes	$f_{1A}/f_{1P}>f_{3A}/f_{3P}>f_{2A}/f_{2P}$	3	Yes	0.9997	0.9988	99.929%
218	644.975	645.184	1533.410	1546.826	2626.208	2628.160	0.9997	0.9913	0.9993	54.52	258.74	125.08	2.36	Yes	$f_{1A}/f_{1P}>f_{3A}/f_{3P}>f_{2A}/f_{2P}$	3	Yes	1.0001	0.9920	-
219	639.554	640.519	1529.785	1537.650	2626.764	2630.233	0.9985	0.9949	0.9987	86.98	116.92	99.59	2.13	Yes	$f_{3A}/f_{3P}>f_{1A}/f_{1P}>f_{2A}/f_{2P}$	1	Yes	0.6429	0.9908	71.149%
220	643.262	644.070	1537.891	1540.326	2629.403	2637.812	0.9987	0.9984	0.9968	73.07	185.63	112.75	1.31	Yes	$f_{1A}/f_{1P}>f_{2A}/f_{2P}>f_{3A}/f_{3P}$	2	Yes	0.9970	0.9946	99.801%
221	644.158	648.741	1535.222	1536.227	2620.660	2628.069	0.9929	0.9993	0.9972	86.78	382.65	133.93	1.56	Yes	$f_{2A}/f_{2P}>f_{3A}/f_{3P}>f_{1A}/f_{1P}$	5	Yes	0.9933	0.9910	99.805%
222	644.147	644.362	1534.290	1541.521	2627.196	2628.040	0.9997	0.9953	0.9997	43.17	255.36	57.56	2.48	Yes	$f_{3A}/f_{3P}>f_{1A}/f_{1P}>f_{2A}/f_{2P}$	1	Yes	0.6446	0.9970	70.780%
223	643.892	644.918	1536.243	1538.328	2625.271	2627.912	0.9984	0.9986	0.9990	24.03	341.83	105.33	1.73	Yes	$f_{3A}/f_{3P}>f_{2A}/f_{2P}>f_{1A}/f_{1P}$	4	Yes	0.9988	0.9978	99.919%
224	642.971	643.764	1538.013	1538.147	2629.495	2635.400	0.9988	0.9999	0.9978	31.76	170.79	129.65	2	Yes	$f_{2A}/f_{2P}>f_{1A}/f_{1P}>f_{3A}/f_{3P}$	2	Yes	0.9971	0.9959	99.901%
225	642.820	643.094	1535.300	1537.717	2628.517	2629.888	0.9996	0.9984	0.9995	46.66	111.56	61.69	1.37	Yes	$f_{1A}/f_{1P}>f_{3A}/f_{3P}>f_{2A}/f_{2P}$	3	Yes	0.9989	0.9980	99.929%
226	643.375	647.742	1535.471	1535.952	2621.489	2627.138	0.9933	0.9997	0.9978	45.66	381.79	123.57	2.28	Yes	$f_{2A}/f_{2P}>f_{3A}/f_{3P}>f_{1A}/f_{1P}$	5	No	-	0.9936	-
227	643.787	644.280	1537.081	1540.557	2629.015	2633.482	0.9992	0.9977	0.9983	59.52	212.21	124.95	0.95	Yes	$f_{1A}/f_{1P}>f_{3A}/f_{3P}>f_{2A}/f_{2P}$	3	Yes	0.9950	0.9965	99.880%
228	644.284	644.779	1534.629	1541.637	2621.871	2628.795	0.9992	0.9955	0.9974	31.35	290.07	94.78	2.45	Yes	$f_{1A}/f_{1P}>f_{3A}/f_{3P}>f_{2A}/f_{2P}$	3	Yes	0.9949	0.9964	99.876%
229	643.455	643.877	1537.398	1538.585	2628.908	2633.020	0.9993	0.9992	0.9984	40	184.93	126.79	0.99	Yes	$f_{1A}/f_{1P}>f_{2A}/f_{2P}>f_{3A}/f_{3P}$	2	Yes	0.9998	0.9975	99.808%
230	643.773	644.677	1536.431	1542.938	2628.794	2635.476	0.9986	0.9958	0.9975	47.52	217.76	129.07	1.84	Yes	$f_{1A}/f_{1P}>f_{3A}/f_{3P}>f_{2A}/f_{2P}$	3	Yes	0.9875	0.9944	99.429%
231	644.243	647.287	1532.697	1541.532	2614.810	2623.983	0.9953	0.9943	0.9965	80.15	329.88	96.87	2.16	Yes	$f_{3A}/f_{3P}>f_{1A}/f_{1P}>f_{2A}/f_{2P}$	1	Yes	0.6383	0.9917	70.702%
232	643.042	643.282	1535.987	1537.330	2628.429	2629.513	0.9996	0.9991	0.9996	75.21	105.32	48.82	0.76	Yes	$f_{1A}/f_{1P}>f_{3A}/f_{3P}>f_{2A}/f_{2P}$	3	Yes	0.9995	0.9986	99.925%
233	642.751	646.292	1534.782	1536.513	2620.102	2632.037	0.9945	0.9989	0.9955	65.56	408.47	54.56	2.22	Yes	$f_{2A}/f_{2P}>f_{3A}/f_{3P}>f_{1A}/f_{1P}$	5	Yes	0.9948	0.9961	99.895%
234	642.115	642.795	1534.585	1537.142	2627.266	2630.082	0.9989	0.9983	0.9989	71.41	99.6	88.8	0.89	Yes	$f_{1A}/f_{1P}>f_{3A}/f_{3P}>f_{2A}/f_{2P}$	3	Yes	0.9915	0.9972	99.534%
235	643.424	643.998	1537.562	1538.823	2628.276	2635.288	0.9991	0.9992	0.9973	78.31	180.74	55.31	1.43	Yes	$f_{2A}/f_{2P}>f_{1A}/f_{1P}>f_{3A}/f_{3P}$	2	Yes	0.9987	0.9969	99.851%
236	644.627	644.662	1535.379	1543.034	2627.781	2628.844	0.9999	0.9950	0.9996	48.11	259.61	124.73	1.54	Yes	$f_{1A}/f_{1P}>f_{3A}/f_{3P}>f_{2A}/f_{2P}$	3	Yes	1.0032	0.9954	-
237	644.223	644.393	1536.108	1540.033	2626.481	2628.578	0.9997	0.9975	0.9992	75.58	277.14	80.08	0.84	Yes	$f_{1A}/f_{1P}>f_{3A}/f_{3P}>f_{2A}/f_{2P}$	3	Yes	1.0008	0.9974	-
238	643.531	643.961	1537.433	1538.658	2628.557	2633.806	0.9993	0.9992	0.9980	80.36	185.28	55.62	1.11	Yes	$f_{1A}/f_{1P}>f_{2A}/f_{2P}>f_{3A}/f_{3P}$	2	Yes	0.9998	0.9975	99.810%
239	640.975	642.210	1531.543	1537.445	2623.434	2630.609	0.9981	0.9962	0.9973	50.82	96.97	53.98	2.58	Yes	$f_{1A}/f_{1P}>f_{3A}/f_{3P}>f_{2A}/f_{2P}$	3	Yes	0.9815	0.9965	98.753%
240	644.124	647.189	1532.542	1541.665	2614.698	2624.316	0.9953	0.9941	0.9963	72.02	329.09	98.94	2.26	Yes	$f_{3A}/f_{3P}>f_{1A}/f_{1P}>f_{2A}/f_{2P}$	1	Yes	0.6383	0.9920	70.670%
241	643.939	644.481	1535.883	1538.975	2625.214	2628.797	0.9992	0.9980	0.9986	42.06	311.54	57.06	1.45	Yes	$f_{1A}/f_{1P}>f_{3A}/f_{3P}>f_{2A}/f_{2P}$	3	Yes	0.9941	0.9983	99.655%
242	643.884	645.154	1534.083	1539.948	2621.225	2627.846	0.9980	0.9962	0.9975	72.85	317.35	49.67	2	Yes	$f_{1A}/f_{1P}>f_{3A}/f_{3P}>f_{2A}/f_{2P}$	3	Yes	0.9809	0.9964	98.714%

243	642.388	642.871	1534.889	1537.426	2628.249	2630.070	0.9992	0.9983	0.9993	56.61	108.76	101.01	0.95	Yes	$f_{3A}/f_{3P}>f_{1A}/f_{1P}>f_{2A}/f_{2P}$	1	Yes	0.6440	0.9973	70.707%
244	643.280	644.369	1537.041	1540.717	2628.569	2635.993	0.9983	0.9976	0.9972	32.64	201.04	120.16	2.3	Yes	$f_{1A}/f_{1P}>f_{2A}/f_{2P}>f_{3A}/f_{3P}$	2	Yes	0.9949	0.9955	99.948%
245	643.963	644.632	1537.126	1542.605	2629.276	2635.933	0.9990	0.9964	0.9975	88.07	216.8	109.01	1.23	Yes	$f_{1A}/f_{1P}>f_{3A}/f_{3P}>f_{2A}/f_{2P}$	3	Yes	0.9918	0.9941	99.810%
246	643.612	643.846	1537.102	1538.035	2628.427	2631.158	0.9996	0.9994	0.9990	59.44	193.43	66.43	0.65	Yes	$f_{1A}/f_{1P}>f_{2A}/f_{2P}>f_{3A}/f_{3P}$	2	Yes	1.0012	0.9987	-
247	643.632	643.838	1537.148	1537.747	2628.496	2630.504	0.9997	0.9996	0.9992	28.02	190.18	86.51	0.76	Yes	$f_{1A}/f_{1P}>f_{2A}/f_{2P}>f_{3A}/f_{3P}$	2	Yes	1.0014	0.9991	-
248	645.030	645.252	1534.963	1544.294	2625.322	2628.403	0.9997	0.9940	0.9988	85.08	269.54	102.25	1.5	Yes	$f_{1A}/f_{1P}>f_{3A}/f_{3P}>f_{2A}/f_{2P}$	3	Yes	0.9998	0.9935	99.476%
249	643.634	643.869	1537.205	1538.014	2628.620	2631.012	0.9996	0.9995	0.9991	35.61	191.08	103.09	0.67	Yes	$f_{1A}/f_{1P}>f_{2A}/f_{2P}>f_{3A}/f_{3P}$	2	Yes	1.0012	0.9988	-
250	643.384	645.450	1535.788	1537.230	2625.426	2625.447	0.9968	0.9991	1.0000	38.43	365.46	61.81	2.4	Yes	$f_{3A}/f_{3P}>f_{2A}/f_{2P}>f_{1A}/f_{1P}$	4	Yes	0.9967	0.9972	99.960%
251	643.732	644.532	1536.849	1541.450	2628.002	2636.840	0.9988	0.9970	0.9966	87.72	206.85	67.48	1.66	Yes	$f_{1A}/f_{1P}>f_{2A}/f_{2P}>f_{3A}/f_{3P}$	2	Yes	0.9971	0.9951	99.838%
252	644.236	644.710	1535.751	1540.364	2625.016	2628.390	0.9993	0.9970	0.9987	66.43	293.15	106.2	0.89	Yes	$f_{1A}/f_{1P}>f_{3A}/f_{3P}>f_{2A}/f_{2P}$	3	Yes	0.9953	0.9968	99.872%
253	643.090	647.789	1533.494	1536.805	2619.830	2636.395	0.9927	0.9978	0.9937	48.77	428.36	115.93	2.04	Yes	$f_{2A}/f_{2P}>f_{3A}/f_{3P}>f_{1A}/f_{1P}$	5	Yes	0.9931	0.9943	99.903%
254	643.992	644.138	1536.631	1539.633	2628.743	2629.537	0.9998	0.9981	0.9997	48.12	242.65	134.78	0.66	Yes	$f_{1A}/f_{1P}>f_{3A}/f_{3P}>f_{2A}/f_{2P}$	3	Yes	1.0012	0.9978	-
255	643.431	643.909	1537.480	1537.842	2628.061	2633.060	0.9993	0.9998	0.9981	33	173.66	57.26	1.92	Yes	$f_{2A}/f_{2P}>f_{1A}/f_{1P}>f_{3A}/f_{3P}$	2	Yes	0.9994	0.9982	99.900%
256	643.720	644.289	1536.529	1537.474	2627.106	2628.010	0.9991	0.9994	0.9997	47.13	349.52	46.3	0.97	Yes	$f_{3A}/f_{3P}>f_{2A}/f_{2P}>f_{1A}/f_{1P}$	4	Yes	0.9998	0.9989	99.928%
257	644.368	647.567	1534.162	1540.048	2619.192	2623.484	0.9951	0.9962	0.9984	83.64	343.59	113.2	1.73	Yes	$f_{3A}/f_{3P}>f_{2A}/f_{2P}>f_{1A}/f_{1P}$	4	Yes	0.9943	0.9918	99.796%
258	644.540	644.675	1535.672	1541.795	2626.253	2628.308	0.9998	0.9960	0.9992	87.24	268.92	90.13	1.11	Yes	$f_{1A}/f_{1P}>f_{3A}/f_{3P}>f_{2A}/f_{2P}$	3	Yes	1.0014	0.9956	-
259	644.114	644.667	1535.959	1539.547	2625.051	2628.400	0.9991	0.9977	0.9987	65.02	305.65	92.95	0.84	Yes	$f_{1A}/f_{1P}>f_{3A}/f_{3P}>f_{2A}/f_{2P}$	3	Yes	0.9939	0.9974	99.706%
260	643.558	644.802	1536.654	1536.966	2626.599	2628.196	0.9981	0.9998	0.9994	41.11	378.28	48.52	1.62	Yes	$f_{2A}/f_{2P}>f_{3A}/f_{3P}>f_{1A}/f_{1P}$	5	Yes	0.9982	0.9984	99.983%
261	643.838	645.797	1536.388	1536.496	2625.246	2629.631	0.9970	0.9999	0.9983	74.31	393.03	122.78	0.78	Yes	$f_{2A}/f_{2P}>f_{3A}/f_{3P}>f_{1A}/f_{1P}$	5	Yes	0.9971	0.9964	99.944%
262	643.875	645.075	1536.028	1538.332	2625.594	2627.346	0.9981	0.9985	0.9993	49.03	344.11	113.41	0.96	Yes	$f_{3A}/f_{3P}>f_{2A}/f_{2P}>f_{1A}/f_{1P}$	4	Yes	0.9985	0.9973	99.902%
263	644.196	646.219	1535.001	1540.114	2622.447	2626.155	0.9969	0.9967	0.9986	61.96	334.07	133.71	1.31	Yes	$f_{3A}/f_{3P}>f_{1A}/f_{1P}>f_{2A}/f_{2P}$	1	Yes	0.6406	0.9946	70.648%
264	643.907	644.666	1534.672	1539.826	2623.044	2628.822	0.9988	0.9967	0.9978	44.51	306.25	50.61	2.19	Yes	$f_{1A}/f_{1P}>f_{3A}/f_{3P}>f_{2A}/f_{2P}$	3	Yes	0.9901	0.9976	99.382%
265	643.908	644.982	1535.954	1538.668	2625.300	2627.625	0.9983	0.9982	0.9991	41.52	335.28	122.4	1.03	Yes	$f_{3A}/f_{3P}>f_{1A}/f_{1P}>f_{2A}/f_{2P}$	1	Yes	0.6427	0.9974	70.588%
266	641.223	642.306	1532.774	1536.942	2625.732	2630.257	0.9983	0.9973	0.9983	53.31	100.49	112.2	1.37	Yes	$f_{1A}/f_{1P}>f_{3A}/f_{3P}>f_{2A}/f_{2P}$	3	Yes	0.9842	0.9959	99.032%
267	643.885	644.009	1536.523	1538.890	2628.532	2628.899	0.9998	0.9985	0.9999	30.36	245.86	85.98	1.01	Yes	$f_{3A}/f_{3P}>f_{1A}/f_{1P}>f_{2A}/f_{2P}$	1	Yes	0.6448	0.9987	70.654%
268	644.139	645.900	1536.082	1537.883	2625.857	2625.925	0.9973	0.9988	1.0000	86.09	360.35	112.13	0.89	Yes	$f_{3A}/f_{3P}>f_{2A}/f_{2P}>f_{1A}/f_{1P}$	4	Yes	0.9973	0.9957	99.866%
269	642.312	642.670	1534.498	1538.192	2629.580	2630.634	0.9994	0.9976	0.9996	59.1	117.03	78.54	1.54	Yes	$f_{3A}/f_{3P}>f_{1A}/f_{1P}>f_{2A}/f_{2P}$	1	Yes	0.6442	0.9964	70.793%
270	643.917	644.553	1536.196	1538.454	2625.657	2628.205	0.9990	0.9985	0.9990	60.25	326.05	75.34	0.82	Yes	$f_{3A}/f_{3P}>f_{1A}/f_{1P}>f_{2A}/f_{2P}$	1	Yes	0.6436	0.9981	70.606%
271	643.758	644.751	1536.324	1537.717	2626.440	2627.554	0.9985	0.9991	0.9996	36.46	352.73	89.59	1.14	Yes	$f_{3A}/f_{3P}>f_{2A}/f_{2P}>f_{1A}/f_{1P}$	4	Yes	0.9989	0.9981	99.936%
272	643.470	643.808	1537.395	1537.686	2628.596	2631.808	0.9995	0.9998	0.9988	26.19	174.47	90.65	1.27	Yes	$f_{2A}/f_{2P}>f_{1A}/f_{1P}>f_{3A}/f_{3P}$	2	Yes	1.0004	0.9985	-
273	643.811	644.420	1536.457	1537.805	2626.572	2628.146	0.9991	0.9991	0.9994	53.23	341.33	57.38	0.89	Yes	$f_{3A}/f_{3P}>f_{2A}/f_{2P}>f_{1A}/f_{1P}$	4	Yes	0.9997	0.9986	99.912%

274	643.538	644.202	1537.154	1539.743	2628.664	2633.913	0.9990	0.9983	0.9980	29.69	201.16	113.76	1.77	Yes	$f_{1A}/f_{1P}>f_{2A}/f_{2P}>f_{3A}/f_{3P}$	2	Yes	0.9981	0.9970	99.911%
275	643.832	645.792	1533.920	1539.679	2619.785	2626.273	0.9970	0.9963	0.9975	79.99	331.49	59.41	2.02	Yes	$f_{3A}/f_{3P}>f_{1A}/f_{1P}>f_{2A}/f_{2P}$	1	Yes	0.6407	0.9952	70.604%
276	644.718	645.839	1534.148	1543.581	2620.900	2627.913	0.9983	0.9939	0.9973	64.31	296.07	118.81	1.66	Yes	$f_{1A}/f_{1P}>f_{3A}/f_{3P}>f_{2A}/f_{2P}$	3	Yes	0.9836	0.9937	99.165%
277	642.203	644.205	1537.396	1542.723	2627.039	2636.730	0.9969	0.9965	0.9963	78.86	186.49	117.61	2.54	Yes	$f_{1A}/f_{1P}>f_{2A}/f_{2P}>f_{3A}/f_{3P}$	2	Yes	0.9882	0.9884	99.987%
278	644.842	645.283	1534.475	1544.369	2624.282	2628.533	0.9993	0.9936	0.9984	55.43	275.77	129.02	1.72	Yes	$f_{1A}/f_{1P}>f_{3A}/f_{3P}>f_{2A}/f_{2P}$	3	Yes	0.9959	0.9939	99.832%
279	643.502	644.725	1536.056	1537.259	2625.814	2631.232	0.9981	0.9992	0.9979	25.75	442.36	52.54	1.86	Yes	$f_{2A}/f_{2P}>f_{1A}/f_{1P}>f_{3A}/f_{3P}$	2	No	-	0.9987	-
280	642.923	646.155	1535.303	1536.378	2621.705	2630.194	0.9950	0.9993	0.9968	46.23	397.31	64.18	2.44	Yes	$f_{2A}/f_{2P}>f_{3A}/f_{3P}>f_{1A}/f_{1P}$	5	Yes	0.9952	0.9964	99.899%
281	643.380	645.969	1534.952	1536.895	2623.198	2633.527	0.9960	0.9987	0.9961	58.34	433.97	91.76	1.25	Yes	$f_{2A}/f_{2P}>f_{3A}/f_{3P}>f_{1A}/f_{1P}$	5	Yes	0.9962	0.9966	99.963%
282	643.628	644.593	1536.761	1542.112	2628.408	2636.856	0.9985	0.9965	0.9968	54.46	208.54	107.39	1.77	Yes	$f_{1A}/f_{1P}>f_{3A}/f_{3P}>f_{2A}/f_{2P}$	3	Yes	0.9864	0.9948	99.300%
283	644.131	644.899	1534.477	1544.606	2627.984	2630.856	0.9988	0.9934	0.9989	44.95	240.8	108.39	2.46	Yes	$f_{3A}/f_{3P}>f_{1A}/f_{1P}>f_{2A}/f_{2P}$	1	Yes	0.6433	0.9941	70.918%
284	643.258	644.013	1537.751	1539.718	2629.048	2636.868	0.9988	0.9987	0.9970	67.25	183.95	104.86	1.29	Yes	$f_{1A}/f_{1P}>f_{2A}/f_{2P}>f_{3A}/f_{3P}$	2	Yes	0.9974	0.9954	99.838%
285	643.265	646.816	1535.403	1536.227	2622.366	2629.399	0.9945	0.9995	0.9973	38.97	391.93	102.48	2.33	Yes	$f_{2A}/f_{2P}>f_{3A}/f_{3P}>f_{1A}/f_{1P}$	5	Yes	0.9948	0.9954	99.951%
286	644.668	645.764	1533.986	1543.078	2620.044	2627.761	0.9983	0.9941	0.9971	77.46	297.72	94.33	1.69	Yes	$f_{1A}/f_{1P}>f_{3A}/f_{3P}>f_{2A}/f_{2P}$	3	Yes	0.9841	0.9938	99.192%
287	643.926	644.227	1536.028	1539.972	2628.296	2629.738	0.9995	0.9974	0.9995	35.36	239.21	62.7	1.82	Yes	$f_{1A}/f_{1P}>f_{3A}/f_{3P}>f_{2A}/f_{2P}$	3	Yes	0.9984	0.9980	99.966%
288	643.094	643.309	1535.977	1537.424	2628.639	2629.488	0.9997	0.9991	0.9997	50.14	110.41	68.84	0.8	Yes	$f_{3A}/f_{3P}>f_{1A}/f_{1P}>f_{2A}/f_{2P}$	1	Yes	0.6446	0.9986	70.646%
289	644.049	644.062	1536.149	1539.539	2627.861	2628.548	1.0000	0.9978	0.9997	38.92	261.03	97.63	0.98	Yes	$f_{1A}/f_{1P}>f_{3A}/f_{3P}>f_{2A}/f_{2P}$	3	Yes	1.0036	0.9981	-
290	644.159	644.708	1535.601	1540.381	2624.710	2628.385	0.9991	0.9969	0.9986	31.52	296.07	133.74	1.44	Yes	$f_{1A}/f_{1P}>f_{3A}/f_{3P}>f_{2A}/f_{2P}$	3	Yes	0.9939	0.9970	99.746%
291	639.896	641.304	1530.726	1536.996	2625.879	2630.760	0.9978	0.9959	0.9981	77.83	105.62	107.68	1.68	Yes	$f_{3A}/f_{3P}>f_{1A}/f_{1P}>f_{2A}/f_{2P}$	1	Yes	0.6419	0.9930	70.889%
292	643.768	644.067	1536.541	1539.299	2628.407	2630.218	0.9995	0.9982	0.9993	30.03	229.17	103.55	1.2	Yes	$f_{1A}/f_{1P}>f_{3A}/f_{3P}>f_{2A}/f_{2P}$	3	Yes	0.9984	0.9981	99.978%
293	642.770	642.822	1536.319	1538.201	2630.492	2633.004	0.9999	0.9988	0.9990	72.97	138.46	114.39	1.01	Yes	$f_{1A}/f_{1P}>f_{3A}/f_{3P}>f_{2A}/f_{2P}$	3	Yes	1.0029	0.9958	-
294	644.338	645.030	1533.285	1545.606	2627.457	2629.397	0.9989	0.9920	0.9993	55.17	246.3	95.42	2.56	Yes	$f_{3A}/f_{3P}>f_{1A}/f_{1P}>f_{2A}/f_{2P}$	1	Yes	0.6435	0.9933	70.993%
295	643.630	646.055	1536.162	1536.562	2624.957	2628.997	0.9962	0.9997	0.9985	25.72	390.15	131.13	2.06	Yes	$f_{2A}/f_{2P}>f_{3A}/f_{3P}>f_{1A}/f_{1P}$	5	Yes	0.9964	0.9965	99.988%
296	642.521	642.708	1534.039	1538.378	2629.951	2630.182	0.9997	0.9972	0.9999	32.56	127.87	79.47	2.58	Yes	$f_{3A}/f_{3P}>f_{1A}/f_{1P}>f_{2A}/f_{2P}$	1	Yes	0.6446	0.9967	70.804%
297	643.360	646.703	1534.453	1536.962	2621.595	2635.145	0.9948	0.9984	0.9949	73.45	432.78	81.84	1.5	Yes	$f_{2A}/f_{2P}>f_{3A}/f_{3P}>f_{1A}/f_{1P}$	5	Yes	0.9951	0.9955	99.967%
298	643.808	645.796	1535.797	1537.748	2625.543	2625.741	0.9969	0.9987	0.9999	62.44	360.71	102.44	1.21	Yes	$f_{3A}/f_{3P}>f_{2A}/f_{2P}>f_{1A}/f_{1P}$	4	Yes	0.9968	0.9961	99.944%
299	644.063	644.698	1536.003	1542.759	2628.520	2633.140	0.9990	0.9956	0.9982	54.88	229.72	105.34	1.63	Yes	$f_{1A}/f_{1P}>f_{3A}/f_{3P}>f_{2A}/f_{2P}$	3	Yes	0.9924	0.9953	99.760%
300	643.857	644.140	1535.859	1539.550	2628.204	2628.834	0.9996	0.9976	0.9998	25.46	243.6	52.04	2.51	Yes	$f_{3A}/f_{3P}>f_{1A}/f_{1P}>f_{2A}/f_{2P}$	1	Yes	0.6444	0.9984	70.651%
301	643.999	645.338	1535.742	1539.040	2624.587	2627.284	0.9979	0.9979	0.9990	52.15	335.93	123.64	1.05	Yes	$f_{3A}/f_{3P}>f_{1A}/f_{1P}>f_{2A}/f_{2P}$	1	No	-	0.9966	-
302	643.144	647.784	1533.367	1536.816	2619.213	2637.506	0.9928	0.9978	0.9931	70.04	436.73	98.22	1.77	Yes	$f_{2A}/f_{2P}>f_{3A}/f_{3P}>f_{1A}/f_{1P}$	5	Yes	0.9932	0.9939	99.939%
303	642.731	643.358	1536.566	1538.459	2629.561	2634.841	0.9990	0.9988	0.9980	39.53	152.29	101.78	2.05	Yes	$f_{1A}/f_{1P}>f_{2A}/f_{2P}>f_{3A}/f_{3P}$	2	Yes	0.9983	0.9959	99.800%
304	643.625	644.027	1537.049	1539.149	2628.547	2632.532	0.9994	0.9986	0.9985	49.94	203.02	104.06	0.89	Yes	$f_{1A}/f_{1P}>f_{2A}/f_{2P}>f_{3A}/f_{3P}$	2	No	-	0.9977	-

305	644.800	646.835	1531.607	1545.321	2613.745	2625.826	0.9969	0.9911	0.9954	72.46	302.62	110.64	2.34	Yes	$f_{1A}/f_{1P}>f_{3A}/f_{3P}>f_{2A}/f_{2P}$	3	Yes	0.9673	0.9907	98.059%
306	643.988	645.752	1534.840	1539.707	2621.590	2626.998	0.9973	0.9968	0.9979	66.32	333.26	77.84	1.67	Yes	$f_{3A}/f_{3P}>f_{1A}/f_{1P}>f_{2A}/f_{2P}$	1	Yes	0.6411	0.9957	70.601%
307	644.075	645.902	1534.651	1540.505	2620.280	2627.127	0.9972	0.9962	0.9974	40.36	327.46	110.97	2.11	Yes	$f_{3A}/f_{3P}>f_{1A}/f_{1P}>f_{2A}/f_{2P}$	1	Yes	0.6410	0.9953	70.622%
308	644.599	645.117	1535.537	1542.042	2624.597	2628.191	0.9992	0.9958	0.9986	79.77	285.25	118.68	1.03	Yes	$f_{1A}/f_{1P}>f_{3A}/f_{3P}>f_{2A}/f_{2P}$	3	Yes	0.9945	0.9951	99.950%
309	643.612	645.248	1535.838	1536.703	2625.102	2630.805	0.9975	0.9994	0.9978	79.79	411.86	68.77	0.94	Yes	$f_{2A}/f_{2P}>f_{3A}/f_{3P}>f_{1A}/f_{1P}$	5	Yes	0.9976	0.9974	99.984%
310	641.953	642.337	1533.106	1537.925	2628.082	2630.614	0.9994	0.9969	0.9990	34.62	116.72	117.51	2.05	Yes	$f_{1A}/f_{1P}>f_{3A}/f_{3P}>f_{2A}/f_{2P}$	3	Yes	0.9969	0.9959	99.914%
311	643.670	645.341	1535.922	1537.772	2625.617	2626.461	0.9974	0.9988	0.9997	58.16	358.41	62.28	1.57	Yes	$f_{3A}/f_{3P}>f_{2A}/f_{2P}>f_{1A}/f_{1P}$	4	Yes	0.9975	0.9972	99.972%
312	643.365	644.539	1537.157	1542.437	2628.806	2638.986	0.9982	0.9966	0.9961	57.34	200.19	123.28	1.88	Yes	$f_{1A}/f_{1P}>f_{2A}/f_{2P}>f_{3A}/f_{3P}$	2	Yes	0.9943	0.9934	99.924%
313	643.676	643.974	1537.035	1538.554	2628.449	2631.395	0.9995	0.9990	0.9989	41.78	204.1	85.27	0.85	Yes	$f_{1A}/f_{1P}>f_{2A}/f_{2P}>f_{3A}/f_{3P}$	2	Yes	1.0007	0.9985	-
314	642.859	643.406	1537.253	1538.390	2629.952	2635.078	0.9991	0.9993	0.9981	42.03	156.2	122.83	1.69	Yes	$f_{2A}/f_{2P}>f_{1A}/f_{1P}>f_{3A}/f_{3P}$	2	No	-	0.9957	-
315	643.349	645.906	1534.906	1537.150	2623.574	2633.087	0.9960	0.9985	0.9964	29.07	426.77	92.84	2.17	Yes	$f_{2A}/f_{2P}>f_{3A}/f_{3P}>f_{1A}/f_{1P}$	5	Yes	0.9962	0.9971	99.926%
316	642.832	643.121	1536.705	1538.273	2630.041	2633.864	0.9996	0.9990	0.9985	51.78	147.65	120.08	1.33	Yes	$f_{1A}/f_{1P}>f_{2A}/f_{2P}>f_{3A}/f_{3P}$	2	Yes	1.0008	0.9959	-
317	643.265	644.111	1537.366	1539.277	2627.309	2636.989	0.9987	0.9988	0.9963	61.76	182.78	64.04	1.92	Yes	$f_{2A}/f_{2P}>f_{1A}/f_{1P}>f_{3A}/f_{3P}$	2	Yes	0.9967	0.9962	99.960%
318	644.484	644.946	1535.464	1541.880	2625.261	2628.391	0.9993	0.9958	0.9988	65.11	282.54	128.58	1.05	Yes	$f_{1A}/f_{1P}>f_{3A}/f_{3P}>f_{2A}/f_{2P}$	3	Yes	0.9955	0.9956	99.993%
319	643.156	643.979	1537.840	1538.819	2628.185	2636.940	0.9987	0.9994	0.9967	54.26	175.22	79.32	1.82	Yes	$f_{2A}/f_{2P}>f_{1A}/f_{1P}>f_{3A}/f_{3P}$	2	Yes	0.9969	0.9961	99.934%
320	643.620	644.979	1536.204	1537.398	2625.533	2626.956	0.9979	0.9992	0.9995	24.55	359.95	67.32	2.52	Yes	$f_{3A}/f_{3P}>f_{2A}/f_{2P}>f_{1A}/f_{1P}$	4	Yes	0.9981	0.9979	99.987%
321	643.090	644.433	1537.308	1541.676	2628.268	2639.801	0.9979	0.9972	0.9956	55.73	193.13	112.7	2.11	Yes	$f_{1A}/f_{1P}>f_{2A}/f_{2P}>f_{3A}/f_{3P}$	2	Yes	0.9931	0.9934	99.973%
322	643.116	643.803	1537.655	1537.957	2628.670	2634.773	0.9989	0.9998	0.9977	33.82	166.04	86.54	2.05	Yes	$f_{2A}/f_{2P}>f_{1A}/f_{1P}>f_{3A}/f_{3P}$	2	Yes	0.9979	0.9970	99.927%
323	644.085	647.228	1535.125	1538.746	2623.529	2624.157	0.9951	0.9976	0.9998	67.24	356.55	126.31	1.54	Yes	$f_{3A}/f_{3P}>f_{2A}/f_{2P}>f_{1A}/f_{1P}$	4	Yes	0.9945	0.9935	99.915%
324	644.007	644.147	1536.212	1539.181	2626.984	2628.603	0.9998	0.9981	0.9994	33.37	278.65	112.62	0.9	Yes	$f_{1A}/f_{1P}>f_{3A}/f_{3P}>f_{2A}/f_{2P}$	3	Yes	1.0013	0.9983	-
325	642.853	646.494	1535.252	1536.121	2620.877	2629.737	0.9944	0.9994	0.9966	53.2	394.03	66.33	2.41	Yes	$f_{2A}/f_{2P}>f_{3A}/f_{3P}>f_{1A}/f_{1P}$	5	Yes	0.9946	0.9958	99.901%
326	642.205	642.560	1533.702	1538.524	2630.133	2631.285	0.9994	0.9969	0.9996	34.09	133.07	99.67	2.58	Yes	$f_{3A}/f_{3P}>f_{1A}/f_{1P}>f_{2A}/f_{2P}$	1	Yes	0.6442	0.9957	70.857%
327	643.551	645.179	1536.236	1536.861	2625.797	2629.331	0.9975	0.9996	0.9987	23.58	394.19	80.39	2.17	Yes	$f_{2A}/f_{2P}>f_{3A}/f_{3P}>f_{1A}/f_{1P}$	5	Yes	0.9976	0.9980	99.970%
328	640.462	640.701	1531.553	1538.476	2628.234	2629.919	0.9996	0.9955	0.9994	78.97	130.44	113.7	2.22	Yes	$f_{1A}/f_{1P}>f_{3A}/f_{3P}>f_{2A}/f_{2P}$	3	Yes	0.9995	0.9901	99.222%
329	643.840	644.465	1536.347	1538.088	2626.447	2628.062	0.9990	0.9989	0.9994	34	332.72	121.57	0.78	Yes	$f_{3A}/f_{3P}>f_{1A}/f_{1P}>f_{2A}/f_{2P}$	1	Yes	0.6437	0.9984	70.587%
330	642.968	643.780	1538.047	1538.352	2627.798	2637.686	0.9987	0.9998	0.9963	77.02	167.86	61.55	1.94	Yes	$f_{2A}/f_{2P}>f_{1A}/f_{1P}>f_{3A}/f_{3P}$	2	Yes	0.9970	0.9954	99.870%
331	643.620	643.879	1537.283	1538.016	2628.550	2631.550	0.9996	0.9995	0.9989	44.5	185.38	74.61	0.79	Yes	$f_{1A}/f_{1P}>f_{2A}/f_{2P}>f_{3A}/f_{3P}$	2	Yes	1.0010	0.9987	-
332	639.824	641.671	1530.543	1536.194	2622.733	2630.410	0.9971	0.9963	0.9971	68.09	92.26	103.47	1.59	Yes	$f_{1A}/f_{1P}>f_{3A}/f_{3P}>f_{2A}/f_{2P}$	3	Yes	0.9704	0.9944	98.007%
333	644.438	645.094	1535.013	1545.642	2628.602	2632.344	0.9990	0.9931	0.9986	58.74	239.87	127.91	1.96	Yes	$f_{1A}/f_{1P}>f_{3A}/f_{3P}>f_{2A}/f_{2P}$	3	Yes	0.9920	0.9927	99.944%
334	643.807	644.467	1536.155	1541.384	2628.621	2630.568	0.9990	0.9966	0.9993	24.08	234.15	116.82	2.46	Yes	$f_{3A}/f_{3P}>f_{1A}/f_{1P}>f_{2A}/f_{2P}$	1	Yes	0.6436	0.9966	70.730%
335	644.275	646.390	1533.642	1542.148	2616.623	2626.147	0.9967	0.9945	0.9964	39.09	319.15	130.31	2.57	Yes	$f_{1A}/f_{1P}>f_{3A}/f_{3P}>f_{2A}/f_{2P}$	3	Yes	0.9658	0.9935	97.701%

336	643.124	646.886	1535.245	1536.422	2621.831	2625.402	0.9942	0.9992	0.9986	52.36	375	82.86	2.41	Yes	$f_{2A}/f_{2P}>f_{3A}/f_{3P}>f_{1A}/f_{1P}$	5	Yes	0.9945	0.9948	99.969%
337	643.628	645.511	1536.203	1536.526	2624.748	2627.744	0.9971	0.9998	0.9989	87.93	381.06	53.13	1.43	Yes	$f_{2A}/f_{2P}>f_{3A}/f_{3P}>f_{1A}/f_{1P}$	5	No	-	0.9967	-
338	643.898	644.984	1534.826	1539.743	2622.454	2628.424	0.9983	0.9968	0.9977	48.67	318.41	60.37	1.98	Yes	$f_{1A}/f_{1P}>f_{3A}/f_{3P}>f_{2A}/f_{2P}$	3	Yes	0.9843	0.9971	98.938%
339	643.651	644.052	1536.957	1538.635	2628.082	2631.990	0.9994	0.9989	0.9985	31.8	201.3	59.85	1.63	Yes	$f_{1A}/f_{1P}>f_{2A}/f_{2P}>f_{3A}/f_{3P}$	2	Yes	1.0000	0.9985	-
340	643.164	643.879	1537.529	1537.717	2628.411	2633.814	0.9989	0.9999	0.9979	24.06	170.45	80.87	2.46	Yes	$f_{2A}/f_{2P}>f_{1A}/f_{1P}>f_{3A}/f_{3P}$	2	Yes	0.9977	0.9976	99.994%
341	643.208	651.270	1530.537	1535.017	2613.633	2641.082	0.9876	0.9971	0.9896	89.1	428.66	130.94	2.16	Yes	$f_{2A}/f_{2P}>f_{3A}/f_{3P}>f_{1A}/f_{1P}$	5	Yes	0.9881	0.9875	99.950%
342	644.262	644.688	1534.879	1542.945	2628.099	2629.976	0.9993	0.9948	0.9993	58.5	244.61	80.03	1.91	Yes	$f_{1A}/f_{1P}>f_{3A}/f_{3P}>f_{2A}/f_{2P}$	3	Yes	0.9961	0.9956	99.955%
343	644.398	645.822	1534.364	1541.744	2620.261	2627.613	0.9978	0.9952	0.9972	69.52	311.98	95.7	1.61	Yes	$f_{1A}/f_{1P}>f_{3A}/f_{3P}>f_{2A}/f_{2P}$	3	Yes	0.9782	0.9947	98.635%
344	644.268	645.608	1533.372	1542.211	2618.817	2628.058	0.9979	0.9943	0.9965	61.2	305.67	80.68	2.11	Yes	$f_{1A}/f_{1P}>f_{3A}/f_{3P}>f_{2A}/f_{2P}$	3	Yes	0.9797	0.9948	98.746%
345	643.395	644.531	1535.889	1541.320	2625.364	2638.719	0.9982	0.9965	0.9949	87.56	201.16	55.65	2.32	Yes	$f_{1A}/f_{1P}>f_{2A}/f_{2P}>f_{3A}/f_{3P}$	2	Yes	0.9946	0.9944	99.983%
346	642.863	643.168	1535.683	1537.422	2628.468	2629.750	0.9995	0.9989	0.9995	68.31	106.99	63.81	0.82	Yes	$f_{1A}/f_{1P}>f_{3A}/f_{3P}>f_{2A}/f_{2P}$	3	Yes	0.9983	0.9982	99.989%
347	644.174	644.380	1535.477	1540.439	2624.803	2628.904	0.9997	0.9968	0.9984	26.64	281.76	83.69	2.17	Yes	$f_{1A}/f_{1P}>f_{3A}/f_{3P}>f_{2A}/f_{2P}$	3	Yes	1.0001	0.9976	-
348	643.784	644.100	1536.176	1539.104	2628.075	2629.673	0.9995	0.9981	0.9994	23.94	233.49	47.84	2.47	Yes	$f_{1A}/f_{1P}>f_{3A}/f_{3P}>f_{2A}/f_{2P}$	3	Yes	0.9981	0.9986	99.958%
349	642.148	642.701	1534.684	1537.345	2628.094	2630.264	0.9991	0.9983	0.9992	81.68	105.19	79.87	0.96	Yes	$f_{3A}/f_{3P}>f_{1A}/f_{1P}>f_{2A}/f_{2P}$	1	Yes	0.6438	0.9969	70.725%
350	643.855	644.833	1536.371	1538.052	2625.802	2627.902	0.9985	0.9989	0.9992	24.29	346.53	97.07	1.66	Yes	$f_{3A}/f_{3P}>f_{2A}/f_{2P}>f_{1A}/f_{1P}$	4	Yes	0.9989	0.9980	99.929%
351	643.898	644.535	1535.996	1538.734	2624.534	2628.653	0.9990	0.9982	0.9984	23.18	321.11	70.93	2.13	Yes	$f_{1A}/f_{1P}>f_{3A}/f_{3P}>f_{2A}/f_{2P}$	3	Yes	0.9924	0.9983	99.514%
352	643.700	645.580	1536.395	1536.865	2625.689	2628.340	0.9971	0.9997	0.9990	51.46	380.17	112.23	1.06	Yes	$f_{2A}/f_{2P}>f_{3A}/f_{3P}>f_{1A}/f_{1P}$	5	No	-	0.9969	-
353	643.817	644.471	1536.437	1537.936	2626.526	2628.086	0.9990	0.9990	0.9994	23.92	338.81	110.85	1.1	Yes	$f_{3A}/f_{3P}>f_{2A}/f_{2P}>f_{1A}/f_{1P}$	4	Yes	0.9996	0.9985	99.912%
354	642.630	643.118	1535.319	1537.151	2627.272	2629.679	0.9992	0.9988	0.9991	70.48	95.1	54.37	0.91	Yes	$f_{1A}/f_{1P}>f_{3A}/f_{3P}>f_{2A}/f_{2P}$	3	Yes	0.9950	0.9982	99.732%
355	644.160	644.592	1532.740	1541.732	2622.473	2628.395	0.9993	0.9942	0.9977	56.62	280.17	49.32	2.59	Yes	$f_{1A}/f_{1P}>f_{3A}/f_{3P}>f_{2A}/f_{2P}$	3	Yes	0.9960	0.9964	99.965%
356	644.005	644.109	1535.708	1539.643	2627.373	2628.561	0.9998	0.9974	0.9995	26.4	259.63	53.43	2.36	Yes	$f_{1A}/f_{1P}>f_{3A}/f_{3P}>f_{2A}/f_{2P}$	3	Yes	1.0020	0.9984	-
357	643.770	643.986	1536.655	1538.911	2628.506	2629.712	0.9997	0.9985	0.9995	23.14	230.98	131.26	1.07	Yes	$f_{1A}/f_{1P}>f_{3A}/f_{3P}>f_{2A}/f_{2P}$	3	Yes	0.9999	0.9984	99.873%
358	643.183	644.697	1535.843	1542.613	2625.039	2641.522	0.9977	0.9956	0.9938	83.77	200.4	72.77	2.46	Yes	$f_{1A}/f_{1P}>f_{2A}/f_{2P}>f_{3A}/f_{3P}$	2	Yes	0.9918	0.9926	99.935%
359	641.108	641.752	1533.500	1537.597	2629.999	2630.737	0.9990	0.9973	0.9997	84.74	120.05	120.63	1.3	Yes	$f_{3A}/f_{3P}>f_{1A}/f_{1P}>f_{2A}/f_{2P}$	1	Yes	0.6436	0.9933	70.999%
360	638.952	640.737	1528.931	1536.402	2623.927	2630.433	0.9972	0.9951	0.9975	81	102.82	108.07	1.86	Yes	$f_{3A}/f_{3P}>f_{1A}/f_{1P}>f_{2A}/f_{2P}$	1	Yes	0.6411	0.9919	70.912%
361	643.639	644.377	1536.733	1537.050	2627.232	2629.246	0.9989	0.9998	0.9992	22.56	398.63	47.01	1.62	Yes	$f_{2A}/f_{2P}>f_{3A}/f_{3P}>f_{1A}/f_{1P}$	5	Yes	0.9989	0.9991	99.980%
362	644.563	646.090	1532.538	1544.294	2616.407	2627.342	0.9976	0.9924	0.9958	54.72	300.18	109.48	2.39	Yes	$f_{1A}/f_{1P}>f_{3A}/f_{3P}>f_{2A}/f_{2P}$	3	Yes	0.9764	0.9929	98.631%
363	643.870	645.843	1535.845	1536.576	2624.920	2630.929	0.9969	0.9995	0.9977	87.25	407.41	108.23	0.78	Yes	$f_{2A}/f_{2P}>f_{3A}/f_{3P}>f_{1A}/f_{1P}$	5	Yes	0.9971	0.9963	99.933%
364	643.862	645.043	1536.408	1537.920	2626.356	2627.470	0.9982	0.9990	0.9996	24.84	353.68	124.47	1.47	Yes	$f_{3A}/f_{3P}>f_{2A}/f_{2P}>f_{1A}/f_{1P}$	4	Yes	0.9985	0.9977	99.936%
365	643.944	644.418	1535.909	1539.016	2625.269	2628.651	0.9993	0.9980	0.9987	39.34	307.02	73.21	1.25	Yes	$f_{1A}/f_{1P}>f_{3A}/f_{3P}>f_{2A}/f_{2P}$	3	Yes	0.9953	0.9982	99.760%
366	643.475	647.751	1534.532	1537.049	2620.966	2623.387	0.9934	0.9984	0.9991	73.69	368.72	95.15	2.09	Yes	$f_{3A}/f_{3P}>f_{2A}/f_{2P}>f_{1A}/f_{1P}$	4	Yes	0.9921	0.9927	99.948%

367	642.795	643.637	1538.461	1538.802	2629.840	2637.846	0.9987	0.9998	0.9970	57.17	168.06	124.01	1.69	Yes	$f_{2A}/f_{2P}>f_{1A}/f_{1P}>f_{3A}/f_{3P}$	2	Yes	0.9967	0.9940	99.778%
368	643.241	644.509	1536.976	1541.797	2627.519	2639.820	0.9980	0.9969	0.9953	63.42	196.21	94.32	2.07	Yes	$f_{1A}/f_{1P}>f_{2A}/f_{2P}>f_{3A}/f_{3P}$	2	Yes	0.9936	0.9939	99.979%
369	643.157	643.775	1538.113	1538.308	2629.074	2635.994	0.9990	0.9999	0.9974	62.64	167.42	85.75	1.4	Yes	$f_{2A}/f_{2P}>f_{1A}/f_{1P}>f_{3A}/f_{3P}$	2	Yes	0.9984	0.9962	99.821%
370	643.214	647.614	1532.749	1537.543	2617.478	2619.329	0.9932	0.9969	0.9993	89.74	357.22	73.55	2.51	Yes	$f_{3A}/f_{3P}>f_{2A}/f_{2P}>f_{1A}/f_{1P}$	4	Yes	0.9919	0.9918	99.992%
371	644.269	644.609	1535.377	1540.486	2624.563	2628.114	0.9995	0.9967	0.9986	86.92	284.74	63.72	1.22	Yes	$f_{1A}/f_{1P}>f_{3A}/f_{3P}>f_{2A}/f_{2P}$	3	Yes	0.9977	0.9966	99.910%
372	643.176	646.541	1534.625	1536.906	2622.010	2633.298	0.9948	0.9985	0.9957	39.41	415.99	92.92	2.15	Yes	$f_{2A}/f_{2P}>f_{3A}/f_{3P}>f_{1A}/f_{1P}$	5	Yes	0.9950	0.9961	99.909%
373	644.024	645.661	1532.566	1541.177	2617.424	2626.957	0.9975	0.9944	0.9964	83.65	312.77	56.8	2.3	Yes	$f_{1A}/f_{1P}>f_{3A}/f_{3P}>f_{2A}/f_{2P}$	3	Yes	0.9743	0.9946	98.318%
374	643.892	644.426	1536.121	1538.473	2625.629	2628.392	0.9992	0.9985	0.9989	57.62	319.73	60.25	0.96	Yes	$f_{1A}/f_{1P}>f_{3A}/f_{3P}>f_{2A}/f_{2P}$	3	Yes	0.9942	0.9983	99.658%
375	643.347	643.998	1537.522	1538.408	2628.207	2634.445	0.9990	0.9994	0.9976	33.37	179.64	78.05	1.95	Yes	$f_{2A}/f_{2P}>f_{1A}/f_{1P}>f_{3A}/f_{3P}$	2	Yes	0.9981	0.9975	99.948%
376	643.070	644.108	1537.624	1539.484	2628.367	2637.390	0.9984	0.9988	0.9966	41.46	183.17	103.5	2.1	Yes	$f_{2A}/f_{2P}>f_{1A}/f_{1P}>f_{3A}/f_{3P}$	2	Yes	0.9953	0.9955	99.981%
377	643.847	644.096	1536.260	1538.310	2626.480	2628.641	0.9996	0.9987	0.9992	29.69	300.9	58.45	1.22	Yes	$f_{1A}/f_{1P}>f_{3A}/f_{3P}>f_{2A}/f_{2P}$	3	Yes	0.9993	0.9989	99.970%
378	643.346	646.462	1534.437	1536.949	2621.997	2635.041	0.9952	0.9984	0.9950	68.7	446.83	86.46	1.39	Yes	$f_{2A}/f_{2P}>f_{1A}/f_{1P}>f_{3A}/f_{3P}$	2	No	-	0.9959	-
379	644.036	645.573	1534.009	1540.997	2619.480	2627.875	0.9976	0.9955	0.9968	48.05	317.4	84.18	2.2	Yes	$f_{1A}/f_{1P}>f_{3A}/f_{3P}>f_{2A}/f_{2P}$	3	Yes	0.9762	0.9956	98.393%
380	644.239	644.581	1535.289	1542.056	2628.147	2629.984	0.9995	0.9956	0.9993	68.5	244.06	67.36	1.68	Yes	$f_{1A}/f_{1P}>f_{3A}/f_{3P}>f_{2A}/f_{2P}$	3	Yes	0.9977	0.9961	99.870%
381	644.441	644.466	1536.465	1541.041	2628.005	2628.588	1.0000	0.9970	0.9998	89.56	261.33	116.14	0.73	Yes	$f_{1A}/f_{1P}>f_{3A}/f_{3P}>f_{2A}/f_{2P}$	3	Yes	1.0034	0.9962	-
382	642.128	648.729	1531.682	1535.999	2613.573	2637.348	0.9898	0.9972	0.9910	74.51	420.34	83.84	2.54	Yes	$f_{2A}/f_{2P}>f_{3A}/f_{3P}>f_{1A}/f_{1P}$	5	Yes	0.9903	0.9922	99.846%
383	642.808	644.017	1538.683	1542.312	2630.452	2638.358	0.9981	0.9976	0.9970	89.33	183.05	131.91	1.78	Yes	$f_{1A}/f_{1P}>f_{2A}/f_{2P}>f_{3A}/f_{3P}$	2	Yes	0.9940	0.9896	99.631%
384	643.720	645.560	1535.908	1536.767	2625.217	2631.262	0.9971	0.9994	0.9977	70.52	414.9	113.78	0.73	Yes	$f_{2A}/f_{2P}>f_{3A}/f_{3P}>f_{1A}/f_{1P}$	5	Yes	0.9973	0.9971	99.979%
385	643.924	644.388	1536.355	1538.203	2626.011	2628.175	0.9993	0.9988	0.9992	80.69	322.86	51.15	0.78	Yes	$f_{1A}/f_{1P}>f_{3A}/f_{3P}>f_{2A}/f_{2P}$	3	Yes	0.9955	0.9984	99.761%
386	643.976	644.048	1536.343	1539.154	2628.486	2628.672	0.9999	0.9982	0.9999	54.07	251.02	59.35	0.94	Yes	$f_{3A}/f_{3P}>f_{1A}/f_{1P}>f_{2A}/f_{2P}$	1	Yes	0.6449	0.9985	70.679%
387	642.802	642.934	1536.516	1538.130	2630.201	2633.193	0.9998	0.9990	0.9989	57.1	143.81	130.41	1.13	Yes	$f_{1A}/f_{1P}>f_{2A}/f_{2P}>f_{3A}/f_{3P}$	2	Yes	1.0020	0.9958	-
388	641.182	641.764	1535.837	1538.831	2629.744	2630.236	0.9991	0.9981	0.9998	81.46	150.37	132.89	2.09	Yes	$f_{3A}/f_{3P}>f_{1A}/f_{1P}>f_{2A}/f_{2P}$	1	Yes	0.6437	0.9888	71.388%
389	643.943	644.085	1536.387	1538.507	2626.823	2628.288	0.9998	0.9986	0.9994	87.68	284.26	49.11	0.68	Yes	$f_{1A}/f_{1P}>f_{3A}/f_{3P}>f_{2A}/f_{2P}$	3	Yes	1.0013	0.9985	-
390	643.294	643.379	1536.850	1537.653	2629.521	2631.218	0.9999	0.9995	0.9994	83.39	140.62	68.51	0.66	Yes	$f_{1A}/f_{1P}>f_{2A}/f_{2P}>f_{3A}/f_{3P}$	2	Yes	1.0023	0.9981	-
391	642.566	643.649	1538.194	1538.419	2628.363	2638.413	0.9983	0.9999	0.9962	60.03	165.92	92.07	2.16	Yes	$f_{2A}/f_{2P}>f_{1A}/f_{1P}>f_{3A}/f_{3P}$	2	Yes	0.9950	0.9941	99.923%
392	641.844	642.084	1535.912	1538.948	2631.025	2633.791	0.9996	0.9980	0.9989	86.23	143.36	120.29	1.64	Yes	$f_{1A}/f_{1P}>f_{3A}/f_{3P}>f_{2A}/f_{2P}$	3	Yes	0.9995	0.9915	99.339%
393	643.554	646.947	1535.120	1536.401	2622.425	2632.458	0.9948	0.9992	0.9962	67.34	408.41	117.85	1.29	Yes	$f_{2A}/f_{2P}>f_{3A}/f_{3P}>f_{1A}/f_{1P}$	5	Yes	0.9950	0.9949	99.990%
394	642.700	642.809	1534.673	1538.247	2630.221	2630.340	0.9998	0.9977	1.0000	30.37	128.28	100.12	2.12	Yes	$f_{3A}/f_{3P}>f_{1A}/f_{1P}>f_{2A}/f_{2P}$	1	Yes	0.6448	0.9968	70.813%
395	643.263	644.698	1536.528	1542.867	2626.757	2641.203	0.9978	0.9959	0.9945	76.93	200.15	87.74	2.21	Yes	$f_{1A}/f_{1P}>f_{2A}/f_{2P}>f_{3A}/f_{3P}$	2	Yes	0.9924	0.9926	99.983%
396	644.449	645.213	1532.457	1543.181	2619.872	2628.105	0.9988	0.9931	0.9969	71.79	286.77	66.13	2.3	Yes	$f_{1A}/f_{1P}>f_{3A}/f_{3P}>f_{2A}/f_{2P}$	3	Yes	0.9901	0.9946	99.628%
397	643.149	645.321	1535.545	1537.060	2623.496	2631.642	0.9966	0.9990	0.9969	39.14	416.97	50.14	2.22	Yes	$f_{2A}/f_{2P}>f_{3A}/f_{3P}>f_{1A}/f_{1P}$	5	Yes	0.9968	0.9978	99.914%

398	643.531	643.731	1537.345	1537.453	2628.567	2631.153	0.9997	0.9999	0.9990	39.85	167.51	69.37	0.84	Yes	$f_{2A}/f_{2P}>f_{1A}/f_{1P}>f_{3A}/f_{3P}$	2	Yes	1.0015	0.9988	-
399	644.115	644.226	1535.572	1539.801	2626.724	2628.692	0.9998	0.9973	0.9993	54.32	271.97	47.7	1.5	Yes	$f_{1A}/f_{1P}>f_{3A}/f_{3P}>f_{2A}/f_{2P}$	3	Yes	1.0018	0.9981	-
400	642.606	642.633	1535.378	1538.591	2630.493	2632.610	1.0000	0.9979	0.9992	69.05	131.69	80.53	1.56	Yes	$f_{1A}/f_{1P}>f_{3A}/f_{3P}>f_{2A}/f_{2P}$	3	Yes	1.0034	0.9957	-
401	643.895	644.113	1536.402	1539.552	2628.492	2629.531	0.9997	0.9980	0.9996	29.14	239.58	95.61	1.32	Yes	$f_{1A}/f_{1P}>f_{3A}/f_{3P}>f_{2A}/f_{2P}$	3	Yes	0.9999	0.9982	99.856%
402	643.589	643.865	1537.196	1537.969	2628.518	2631.224	0.9996	0.9995	0.9990	26.55	188.52	96.15	1	Yes	$f_{1A}/f_{1P}>f_{2A}/f_{2P}>f_{3A}/f_{3P}$	2	Yes	1.0009	0.9987	-
403	644.096	646.278	1534.433	1540.574	2620.145	2626.483	0.9966	0.9960	0.9976	53.44	331.36	108.7	1.87	Yes	$f_{3A}/f_{3P}>f_{1A}/f_{1P}>f_{2A}/f_{2P}$	1	Yes	0.6402	0.9946	70.617%
404	637.624	639.976	1525.762	1535.611	2619.125	2629.758	0.9963	0.9936	0.9960	86.95	96.03	75.54	2.49	Yes	$f_{1A}/f_{1P}>f_{3A}/f_{3P}>f_{2A}/f_{2P}$	3	Yes	0.9611	0.9919	97.446%
405	644.482	644.616	1535.013	1542.201	2625.920	2628.498	0.9998	0.9953	0.9990	62.01	268.82	94.81	1.41	Yes	$f_{1A}/f_{1P}>f_{3A}/f_{3P}>f_{2A}/f_{2P}$	3	Yes	1.0014	0.9959	-
406	643.949	645.470	1535.109	1548.278	2627.926	2640.253	0.9976	0.9915	0.9953	81.77	221.7	127.22	2.36	Yes	$f_{1A}/f_{1P}>f_{3A}/f_{3P}>f_{2A}/f_{2P}$	3	Yes	0.9764	0.9878	99.051%
407	644.901	645.084	1534.697	1545.213	2628.347	2628.545	0.9997	0.9932	0.9999	71.59	255.32	110.03	1.73	Yes	$f_{3A}/f_{3P}>f_{1A}/f_{1P}>f_{2A}/f_{2P}$	1	Yes	0.6446	0.9932	71.096%
408	643.442	645.452	1534.029	1538.892	2621.160	2626.438	0.9969	0.9968	0.9980	62.21	338.09	49.17	2.38	Yes	$f_{3A}/f_{3P}>f_{1A}/f_{1P}>f_{2A}/f_{2P}$	1	No	-	0.9964	-
409	644.210	644.388	1535.581	1539.948	2625.812	2628.248	0.9997	0.9972	0.9991	89.97	277.04	46.59	1.3	Yes	$f_{1A}/f_{1P}>f_{3A}/f_{3P}>f_{2A}/f_{2P}$	3	Yes	1.0006	0.9973	-
410	643.553	644.825	1536.654	1536.916	2626.590	2628.064	0.9980	0.9998	0.9994	32.03	378.15	53.66	1.9	Yes	$f_{2A}/f_{2P}>f_{3A}/f_{3P}>f_{1A}/f_{1P}$	5	Yes	0.9981	0.9984	99.981%
411	643.629	644.268	1536.688	1536.823	2627.348	2628.858	0.9990	0.9999	0.9994	23.57	395.28	97.44	0.77	Yes	$f_{2A}/f_{2P}>f_{3A}/f_{3P}>f_{1A}/f_{1P}$	5	Yes	0.9991	0.9991	99.999%
412	643.056	643.611	1537.816	1538.211	2629.656	2635.076	0.9991	0.9997	0.9979	45.64	162.01	117.14	1.44	Yes	$f_{2A}/f_{2P}>f_{1A}/f_{1P}>f_{3A}/f_{3P}$	2	Yes	0.9989	0.9962	99.772%
413	642.426	643.118	1536.765	1538.617	2628.401	2636.684	0.9989	0.9988	0.9969	82.97	152.83	65.67	2.11	Yes	$f_{1A}/f_{1P}>f_{2A}/f_{2P}>f_{3A}/f_{3P}$	2	Yes	0.9978	0.9943	99.706%
414	643.431	644.244	1537.204	1540.043	2628.638	2635.085	0.9987	0.9982	0.9976	33.15	198.12	114.75	1.9	Yes	$f_{1A}/f_{1P}>f_{2A}/f_{2P}>f_{3A}/f_{3P}$	2	Yes	0.9970	0.9964	99.951%
415	644.005	646.435	1533.862	1540.699	2618.515	2626.016	0.9962	0.9956	0.9971	56	331.51	99.9	2.1	Yes	$f_{3A}/f_{3P}>f_{1A}/f_{1P}>f_{2A}/f_{2P}$	1	Yes	0.6397	0.9942	70.607%
416	638.978	640.262	1529.187	1537.039	2625.921	2630.058	0.9980	0.9949	0.9984	84.82	116.04	121.41	2.01	Yes	$f_{3A}/f_{3P}>f_{1A}/f_{1P}>f_{2A}/f_{2P}$	1	Yes	0.6422	0.9897	71.185%
417	643.658	644.179	1536.380	1539.383	2627.327	2632.751	0.9992	0.9980	0.9979	44.38	208.55	49.34	1.95	Yes	$f_{1A}/f_{1P}>f_{2A}/f_{2P}>f_{3A}/f_{3P}$	2	Yes	0.9991	0.9979	99.898%
418	643.794	646.724	1534.554	1539.146	2620.361	2624.772	0.9955	0.9970	0.9983	43.8	347.63	112.55	2.31	Yes	$f_{3A}/f_{3P}>f_{2A}/f_{2P}>f_{1A}/f_{1P}$	4	Yes	0.9949	0.9944	99.955%
419	644.668	645.034	1533.098	1543.854	2622.455	2628.253	0.9994	0.9930	0.9978	67.72	275.38	76.38	2.15	Yes	$f_{1A}/f_{1P}>f_{3A}/f_{3P}>f_{2A}/f_{2P}$	3	Yes	0.9972	0.9945	99.775%
420	643.663	644.339	1535.722	1540.295	2626.649	2633.523	0.9990	0.9970	0.9974	55.45	213.96	49.52	2.15	Yes	$f_{1A}/f_{1P}>f_{3A}/f_{3P}>f_{2A}/f_{2P}$	3	Yes	0.9917	0.9971	99.555%
421	643.306	644.352	1537.097	1540.618	2627.571	2637.995	0.9984	0.9977	0.9960	55.61	193.45	85.15	1.98	Yes	$f_{1A}/f_{1P}>f_{2A}/f_{2P}>f_{3A}/f_{3P}$	2	Yes	0.9952	0.9953	99.988%
422	644.706	647.692	1532.464	1543.908	2614.634	2624.037	0.9954	0.9926	0.9964	68.26	318.71	134.61	2.17	Yes	$f_{3A}/f_{3P}>f_{1A}/f_{1P}>f_{2A}/f_{2P}$	1	Yes	0.6385	0.9901	70.843%
423	643.853	643.939	1536.461	1538.445	2628.385	2628.497	0.9999	0.9987	1.0000	28.01	252.28	52.89	1.28	Yes	$f_{3A}/f_{3P}>f_{1A}/f_{1P}>f_{2A}/f_{2P}$	1	Yes	0.6448	0.9991	70.630%
424	642.784	642.962	1534.839	1537.910	2628.630	2630.181	0.9997	0.9980	0.9994	29.14	116.07	91.11	1.81	Yes	$f_{1A}/f_{1P}>f_{3A}/f_{3P}>f_{2A}/f_{2P}$	3	Yes	1.0006	0.9976	-
425	643.940	646.458	1534.847	1539.504	2621.468	2625.678	0.9961	0.9970	0.9984	44.98	343.58	118.66	1.98	Yes	$f_{3A}/f_{3P}>f_{2A}/f_{2P}>f_{1A}/f_{1P}$	4	Yes	0.9957	0.9947	99.921%
426	644.476	644.704	1532.784	1543.480	2626.730	2627.069	0.9996	0.9931	0.9999	77.68	255.11	57.94	2.37	Yes	$f_{3A}/f_{3P}>f_{1A}/f_{1P}>f_{2A}/f_{2P}$	1	Yes	0.6445	0.9947	70.963%
427	644.255	645.004	1534.489	1541.511	2622.043	2628.749	0.9988	0.9954	0.9974	46.18	296.61	84.41	1.94	Yes	$f_{1A}/f_{1P}>f_{3A}/f_{3P}>f_{2A}/f_{2P}$	3	Yes	0.9903	0.9962	99.508%
428	644.343	647.470	1531.387	1543.368	2611.428	2623.867	0.9952	0.9922	0.9953	71.86	320.34	106.88	2.48	Yes	$f_{3A}/f_{3P}>f_{1A}/f_{1P}>f_{2A}/f_{2P}$	1	Yes	0.6382	0.9906	70.778%

429	643.682	644.310	1537.228	1541.078	2629.131	2635.269	0.9990	0.9975	0.9977	68.88	206.25	122.35	1.08	Yes	$f_{1A}/f_{1P}>f_{3A}/f_{3P}>f_{2A}/f_{2P}$	3	Yes	0.9925	0.9954	99.757%
430	642.903	643.176	1535.821	1537.284	2628.876	2629.457	0.9996	0.9990	0.9998	37.25	117.45	132.5	0.77	Yes	$f_{3A}/f_{3P}>f_{1A}/f_{1P}>f_{2A}/f_{2P}$	1	Yes	0.6444	0.9981	70.673%
431	643.116	643.302	1536.077	1537.389	2628.975	2629.441	0.9997	0.9991	0.9998	31.24	118.42	124.29	0.8	Yes	$f_{3A}/f_{3P}>f_{1A}/f_{1P}>f_{2A}/f_{2P}$	1	Yes	0.6446	0.9984	70.660%
432	644.048	644.199	1536.147	1540.215	2628.525	2628.972	0.9998	0.9974	0.9998	51.77	248.76	97.1	0.98	Yes	$f_{3A}/f_{3P}>f_{1A}/f_{1P}>f_{2A}/f_{2P}$	1	Yes	0.6447	0.9975	70.744%
433	642.706	644.050	1537.948	1540.076	2627.627	2640.121	0.9979	0.9986	0.9953	69.62	178.24	92.16	2.18	Yes	$f_{2A}/f_{2P}>f_{1A}/f_{1P}>f_{3A}/f_{3P}$	2	Yes	0.9931	0.9931	99.997%
434	643.339	643.468	1536.689	1537.743	2629.156	2631.033	0.9998	0.9993	0.9993	45.13	140.48	65.44	1.1	Yes	$f_{1A}/f_{1P}>f_{2A}/f_{2P}>f_{3A}/f_{3P}$	2	Yes	1.0020	0.9984	-
435	644.245	646.388	1535.664	1538.521	2624.639	2625.279	0.9967	0.9981	0.9998	83.44	353.68	127.9	1.02	Yes	$f_{3A}/f_{3P}>f_{2A}/f_{2P}>f_{1A}/f_{1P}$	4	Yes	0.9965	0.9945	99.837%
436	641.481	642.056	1533.482	1537.861	2629.494	2630.728	0.9991	0.9972	0.9995	70.89	117.72	107.57	1.42	Yes	$f_{3A}/f_{3P}>f_{1A}/f_{1P}>f_{2A}/f_{2P}$	1	Yes	0.6438	0.9946	70.913%
437	643.350	643.390	1536.438	1537.478	2629.279	2629.538	0.9999	0.9993	0.9999	31.1	127.23	105.59	0.78	Yes	$f_{1A}/f_{1P}>f_{3A}/f_{3P}>f_{2A}/f_{2P}$	3	Yes	1.0031	0.9987	-
438	644.460	645.483	1535.183	1547.604	2628.289	2637.140	0.9984	0.9920	0.9966	86.26	230.93	118.66	2.07	Yes	$f_{1A}/f_{1P}>f_{3A}/f_{3P}>f_{2A}/f_{2P}$	3	Yes	0.9854	0.9895	99.662%
439	641.677	642.501	1533.295	1537.713	2626.638	2630.673	0.9987	0.9971	0.9985	58.74	103.57	67.86	1.77	Yes	$f_{1A}/f_{1P}>f_{3A}/f_{3P}>f_{2A}/f_{2P}$	3	Yes	0.9889	0.9965	99.372%
440	644.862	645.132	1534.764	1543.932	2625.152	2628.548	0.9996	0.9941	0.9987	73.09	271.23	104.89	1.52	Yes	$f_{1A}/f_{1P}>f_{3A}/f_{3P}>f_{2A}/f_{2P}$	3	Yes	0.9990	0.9942	99.600%
441	643.014	643.161	1535.391	1538.054	2628.876	2630.338	0.9998	0.9983	0.9994	33.61	131.03	47.13	2.6	Yes	$f_{1A}/f_{1P}>f_{3A}/f_{3P}>f_{2A}/f_{2P}$	3	Yes	1.0012	0.9980	-
442	643.273	643.303	1535.881	1537.812	2629.426	2629.469	1.0000	0.9987	1.0000	25.5	123.94	60.33	1.91	Yes	$f_{3A}/f_{3P}>f_{1A}/f_{1P}>f_{2A}/f_{2P}$	1	Yes	0.6450	0.9985	70.685%
443	642.037	642.900	1533.986	1537.196	2625.509	2630.222	0.9987	0.9979	0.9982	41.63	92.21	74.19	1.55	Yes	$f_{1A}/f_{1P}>f_{3A}/f_{3P}>f_{2A}/f_{2P}$	3	Yes	0.9882	0.9976	99.222%
444	643.546	644.368	1537.639	1540.887	2628.793	2638.207	0.9987	0.9979	0.9964	89.76	194.39	75.35	1.52	Yes	$f_{1A}/f_{1P}>f_{2A}/f_{2P}>f_{3A}/f_{3P}$	2	Yes	0.9969	0.9949	99.831%
445	643.527	644.121	1537.099	1539.031	2627.566	2634.488	0.9991	0.9987	0.9974	60.5	189.98	51.39	1.66	Yes	$f_{1A}/f_{1P}>f_{2A}/f_{2P}>f_{3A}/f_{3P}$	2	Yes	0.9986	0.9974	99.903%
446	642.259	642.981	1534.594	1537.108	2626.251	2629.880	0.9989	0.9984	0.9986	55.5	92.46	60.7	1.23	Yes	$f_{1A}/f_{1P}>f_{3A}/f_{3P}>f_{2A}/f_{2P}$	3	Yes	0.9908	0.9979	99.409%
447	643.332	644.867	1536.841	1544.977	2628.618	2641.072	0.9976	0.9947	0.9953	75.66	204.21	125.59	2.12	Yes	$f_{1A}/f_{1P}>f_{3A}/f_{3P}>f_{2A}/f_{2P}$	3	Yes	0.9762	0.9900	98.856%
448	644.305	645.550	1535.310	1540.463	2622.764	2627.328	0.9981	0.9967	0.9983	72.83	318.49	115.95	1.08	Yes	$f_{3A}/f_{3P}>f_{1A}/f_{1P}>f_{2A}/f_{2P}$	1	Yes	0.6423	0.9954	70.719%
449	643.717	644.039	1536.929	1538.978	2628.513	2631.366	0.9995	0.9987	0.9989	49.82	212.94	92.81	0.79	Yes	$f_{1A}/f_{1P}>f_{3A}/f_{3P}>f_{2A}/f_{2P}$	3	Yes	0.9980	0.9982	99.986%
450	643.310	643.508	1536.830	1537.708	2629.099	2631.425	0.9997	0.9994	0.9991	37.46	146.89	83.39	1.12	Yes	$f_{1A}/f_{1P}>f_{2A}/f_{2P}>f_{3A}/f_{3P}$	2	Yes	1.0015	0.9982	-
451	643.768	643.828	1536.672	1537.959	2628.403	2628.559	0.9999	0.9992	0.9999	24.52	247.3	87.92	0.67	Yes	$f_{3A}/f_{3P}>f_{1A}/f_{1P}>f_{2A}/f_{2P}$	1	Yes	0.6449	0.9993	70.616%
452	643.237	643.713	1537.950	1537.996	2628.956	2635.025	0.9993	1.0000	0.9977	89.5	165	52.11	1.4	Yes	$f_{2A}/f_{2P}>f_{1A}/f_{1P}>f_{3A}/f_{3P}$	2	Yes	0.9994	0.9967	99.779%
453	639.779	640.748	1529.914	1537.694	2626.761	2630.262	0.9985	0.9949	0.9987	89.11	113.7	78.8	2.26	Yes	$f_{3A}/f_{3P}>f_{1A}/f_{1P}>f_{2A}/f_{2P}$	1	Yes	0.6429	0.9921	71.042%
454	642.227	642.799	1536.229	1538.869	2630.132	2635.787	0.9991	0.9983	0.9979	60.36	148.93	112.68	1.92	Yes	$f_{1A}/f_{1P}>f_{2A}/f_{2P}>f_{3A}/f_{3P}$	2	Yes	0.9987	0.9935	99.567%
455	643.450	646.429	1534.446	1536.619	2622.119	2634.197	0.9954	0.9986	0.9954	83.57	432.11	82.53	1.3	Yes	$f_{2A}/f_{2P}>f_{3A}/f_{3P}>f_{1A}/f_{1P}$	5	Yes	0.9956	0.9955	99.993%
456	643.596	645.179	1536.347	1536.985	2626.052	2627.611	0.9975	0.9996	0.9994	54.52	374.55	67.94	1.31	Yes	$f_{2A}/f_{2P}>f_{3A}/f_{3P}>f_{1A}/f_{1P}$	5	Yes	0.9977	0.9976	99.991%
457	644.204	644.554	1535.727	1541.494	2628.066	2630.633	0.9995	0.9963	0.9990	87.33	239.37	56.21	1.57	Yes	$f_{1A}/f_{1P}>f_{3A}/f_{3P}>f_{2A}/f_{2P}$	3	Yes	0.9975	0.9962	99.888%
458	644.215	644.991	1535.921	1539.917	2625.095	2627.955	0.9988	0.9974	0.9989	67.01	310.08	134.96	0.74	Yes	$f_{3A}/f_{3P}>f_{1A}/f_{1P}>f_{2A}/f_{2P}$	1	Yes	0.6433	0.9966	70.704%
459	640.818	641.382	1531.774	1538.340	2629.144	2630.612	0.9991	0.9957	0.9994	80.58	119.36	80.96	2.12	Yes	$f_{3A}/f_{3P}>f_{1A}/f_{1P}>f_{2A}/f_{2P}$	1	Yes	0.6438	0.9931	71.034%

460	643.619	644.251	1536.503	1537.105	2627.086	2629.938	0.9990	0.9996	0.9989	28.14	442.89	49.17	1.02	Yes	$f_{2A}/f_{2P}>f_{1A}/f_{1P}>f_{3A}/f_{3P}$	2	No	-	0.9993	-
461	642.208	642.307	1534.960	1538.812	2630.584	2633.498	0.9998	0.9975	0.9989	70.17	135.04	94.63	1.76	Yes	$f_{1A}/f_{1P}>f_{3A}/f_{3P}>f_{2A}/f_{2P}$	3	Yes	1.0021	0.9942	-
462	643.801	643.923	1536.475	1538.016	2627.320	2628.454	0.9998	0.9990	0.9996	22.57	287.29	90.28	0.8	Yes	$f_{1A}/f_{1P}>f_{3A}/f_{3P}>f_{2A}/f_{2P}$	3	Yes	1.0016	0.9992	-
463	642.258	642.413	1534.816	1538.474	2628.899	2633.068	0.9998	0.9976	0.9984	85.52	134.59	51	2.21	Yes	$f_{1A}/f_{1P}>f_{3A}/f_{3P}>f_{2A}/f_{2P}$	3	Yes	1.0010	0.9952	-
464	645.493	645.614	1534.517	1547.032	2626.695	2628.275	0.9998	0.9919	0.9994	84.64	262.75	127.26	1.75	Yes	$f_{1A}/f_{1P}>f_{3A}/f_{3P}>f_{2A}/f_{2P}$	3	Yes	1.0017	0.9906	-
465	643.997	644.835	1536.186	1538.362	2625.282	2627.639	0.9987	0.9986	0.9991	78.7	335.91	79.88	0.79	Yes	$f_{3A}/f_{3P}>f_{1A}/f_{1P}>f_{2A}/f_{2P}$	1	No	-	0.9975	-
466	641.809	642.648	1533.216	1537.005	2625.241	2630.110	0.9987	0.9975	0.9981	28.24	98.48	128.45	1.73	Yes	$f_{1A}/f_{1P}>f_{3A}/f_{3P}>f_{2A}/f_{2P}$	3	Yes	0.9887	0.9969	99.319%
467	643.827	645.234	1536.531	1537.072	2626.454	2628.326	0.9978	0.9996	0.9993	63.04	377.9	124.99	0.66	Yes	$f_{2A}/f_{2P}>f_{3A}/f_{3P}>f_{1A}/f_{1P}$	5	Yes	0.9979	0.9974	99.953%
468	643.589	646.755	1534.681	1536.529	2622.316	2633.669	0.9951	0.9988	0.9957	80.94	420.41	99.93	1.23	Yes	$f_{2A}/f_{2P}>f_{3A}/f_{3P}>f_{1A}/f_{1P}$	5	Yes	0.9953	0.9950	99.977%
469	639.583	640.330	1530.285	1537.624	2626.953	2629.614	0.9988	0.9952	0.9990	76.25	126.6	134.21	2.16	Yes	$f_{3A}/f_{3P}>f_{1A}/f_{1P}>f_{2A}/f_{2P}$	1	Yes	0.6434	0.9890	71.341%
470	643.684	648.847	1534.358	1535.865	2619.641	2633.750	0.9920	0.9990	0.9946	77.54	406.36	132.92	1.62	Yes	$f_{2A}/f_{2P}>f_{3A}/f_{3P}>f_{1A}/f_{1P}$	5	Yes	0.9924	0.9917	99.940%
471	643.710	645.230	1536.629	1536.941	2626.551	2627.799	0.9976	0.9998	0.9995	22.75	377	109.1	1.85	Yes	$f_{2A}/f_{2P}>f_{3A}/f_{3P}>f_{1A}/f_{1P}$	5	Yes	0.9978	0.9977	99.995%
472	643.689	644.205	1536.754	1540.120	2628.401	2633.038	0.9992	0.9978	0.9982	48.18	212.52	102.78	1.2	Yes	$f_{1A}/f_{1P}>f_{3A}/f_{3P}>f_{2A}/f_{2P}$	3	Yes	0.9945	0.9970	99.791%
473	643.650	644.773	1536.777	1543.725	2628.884	2638.661	0.9983	0.9955	0.9963	64.31	209.67	124.84	1.81	Yes	$f_{1A}/f_{1P}>f_{3A}/f_{3P}>f_{2A}/f_{2P}$	3	Yes	0.9836	0.9928	99.240%
474	643.507	645.214	1535.813	1536.983	2625.054	2631.513	0.9974	0.9992	0.9975	51.16	421	76.44	1.11	Yes	$f_{2A}/f_{2P}>f_{3A}/f_{3P}>f_{1A}/f_{1P}$	5	Yes	0.9975	0.9978	99.973%
475	642.612	646.458	1534.532	1535.865	2619.521	2624.766	0.9941	0.9991	0.9980	82.85	376.4	49.65	2.55	Yes	$f_{2A}/f_{2P}>f_{3A}/f_{3P}>f_{1A}/f_{1P}$	5	Yes	0.9943	0.9948	99.959%
476	644.381	644.428	1535.273	1541.600	2626.619	2628.518	0.9999	0.9959	0.9993	56.53	265.55	94.54	1.34	Yes	$f_{1A}/f_{1P}>f_{3A}/f_{3P}>f_{2A}/f_{2P}$	3	Yes	1.0030	0.9964	-
477	643.073	643.420	1536.195	1538.073	2628.786	2632.170	0.9995	0.9988	0.9987	34.61	143.65	59.45	2.24	Yes	$f_{1A}/f_{1P}>f_{2A}/f_{2P}>f_{3A}/f_{3P}$	2	Yes	1.0004	0.9977	-
478	644.163	644.317	1535.634	1539.957	2626.088	2628.522	0.9998	0.9972	0.9991	71.75	276.38	55.5	1.21	Yes	$f_{1A}/f_{1P}>f_{3A}/f_{3P}>f_{2A}/f_{2P}$	3	Yes	1.0010	0.9976	-
479	643.687	644.980	1536.535	1536.946	2626.477	2628.056	0.9980	0.9997	0.9994	63.99	377.21	65.93	1.01	Yes	$f_{2A}/f_{2P}>f_{3A}/f_{3P}>f_{1A}/f_{1P}$	5	Yes	0.9981	0.9979	99.979%
480	644.647	644.909	1533.110	1544.118	2622.308	2628.433	0.9996	0.9929	0.9977	50.64	273.76	88.08	2.43	Yes	$f_{1A}/f_{1P}>f_{3A}/f_{3P}>f_{2A}/f_{2P}$	3	Yes	0.9991	0.9946	99.630%
481	643.740	645.611	1536.480	1537.002	2626.108	2627.544	0.9971	0.9997	0.9995	24.27	375.86	129.78	1.87	Yes	$f_{2A}/f_{2P}>f_{3A}/f_{3P}>f_{1A}/f_{1P}$	5	Yes	0.9973	0.9971	99.985%
482	644.154	644.155	1535.299	1540.196	2626.249	2628.703	1.0000	0.9968	0.9991	30.8	268.01	57.96	2.33	Yes	$f_{1A}/f_{1P}>f_{3A}/f_{3P}>f_{2A}/f_{2P}$	3	Yes	1.0038	0.9979	-
483	640.847	641.837	1532.577	1537.059	2626.993	2630.554	0.9985	0.9971	0.9986	84.32	105.38	96.29	1.33	Yes	$f_{3A}/f_{3P}>f_{1A}/f_{1P}>f_{2A}/f_{2P}$	1	Yes	0.6428	0.9946	70.829%
484	642.305	642.786	1536.174	1538.939	2630.143	2635.560	0.9993	0.9982	0.9979	70.02	146.27	96.65	1.83	Yes	$f_{1A}/f_{1P}>f_{2A}/f_{2P}>f_{3A}/f_{3P}$	2	Yes	0.9994	0.9938	99.539%
485	644.232	645.958	1535.304	1539.462	2622.433	2626.200	0.9973	0.9973	0.9986	82.05	336.73	103.86	1.18	Yes	$f_{3A}/f_{3P}>f_{1A}/f_{1P}>f_{2A}/f_{2P}$	1	No	-	0.9950	-
486	644.128	644.228	1535.013	1540.569	2627.423	2628.328	0.9998	0.9964	0.9997	42.72	257.93	52.71	2.11	Yes	$f_{1A}/f_{1P}>f_{3A}/f_{3P}>f_{2A}/f_{2P}$	3	Yes	1.0020	0.9976	-
487	644.318	645.380	1531.709	1543.508	2617.545	2628.104	0.9984	0.9924	0.9960	61.88	292.16	71.62	2.6	Yes	$f_{1A}/f_{1P}>f_{3A}/f_{3P}>f_{2A}/f_{2P}$	3	Yes	0.9847	0.9943	99.203%
488	644.174	644.373	1535.569	1540.351	2625.938	2628.702	0.9997	0.9969	0.9989	47.98	277.49	80.69	1.29	Yes	$f_{1A}/f_{1P}>f_{3A}/f_{3P}>f_{2A}/f_{2P}$	3	Yes	1.0002	0.9975	-
489	644.131	646.189	1535.202	1539.488	2623.038	2625.976	0.9968	0.9972	0.9989	65.73	340.62	126.97	1.24	Yes	$f_{3A}/f_{3P}>f_{2A}/f_{2P}>f_{1A}/f_{1P}$	4	Yes	0.9967	0.9948	99.844%
490	643.251	648.072	1534.441	1535.918	2619.324	2632.236	0.9926	0.9990	0.9951	73.47	400.77	100.2	1.91	Yes	$f_{2A}/f_{2P}>f_{3A}/f_{3P}>f_{1A}/f_{1P}$	5	Yes	0.9929	0.9930	99.991%

491	643.909	647.575	1535.107	1536.865	2621.753	2625.054	0.9943	0.9989	0.9987	89.84	372.27	95.25	1.71	Yes	$f_{2A}/f_{2P}>f_{3A}/f_{3P}>f_{1A}/f_{1P}$	5	Yes	0.9946	0.9927	99.843%
492	644.027	644.361	1535.675	1539.491	2625.440	2628.938	0.9995	0.9975	0.9987	39.66	292.49	56.59	1.62	Yes	$f_{1A}/f_{1P}>f_{3A}/f_{3P}>f_{2A}/f_{2P}$	3	Yes	0.9978	0.9982	99.968%
493	642.602	643.294	1536.276	1538.287	2629.984	2633.915	0.9989	0.9987	0.9985	29.04	153.89	131.44	2.26	Yes	$f_{1A}/f_{1P}>f_{2A}/f_{2P}>f_{3A}/f_{3P}$	2	Yes	0.9978	0.9957	99.824%
494	643.216	646.721	1535.032	1536.521	2622.145	2631.176	0.9946	0.9990	0.9966	40.03	400.98	97.98	2.23	Yes	$f_{2A}/f_{2P}>f_{3A}/f_{3P}>f_{1A}/f_{1P}$	5	Yes	0.9948	0.9957	99.928%
495	643.843	644.263	1536.462	1538.047	2626.638	2628.402	0.9993	0.9990	0.9993	45.26	323.89	78.62	0.67	Yes	$f_{1A}/f_{1P}>f_{3A}/f_{3P}>f_{2A}/f_{2P}$	3	Yes	0.9963	0.9988	99.793%
496	643.229	644.147	1537.715	1539.701	2628.522	2637.385	0.9986	0.9987	0.9966	50.2	183.65	97.79	1.78	Yes	$f_{2A}/f_{2P}>f_{1A}/f_{1P}>f_{3A}/f_{3P}$	2	Yes	0.9962	0.9956	99.954%
497	643.427	646.151	1534.974	1536.990	2623.500	2633.610	0.9958	0.9987	0.9962	39.95	438.75	119.86	1.42	Yes	$f_{2A}/f_{2P}>f_{3A}/f_{3P}>f_{1A}/f_{1P}$	5	Yes	0.9960	0.9966	99.950%
498	644.424	646.293	1533.543	1542.940	2617.565	2626.746	0.9971	0.9939	0.9965	45.26	311.89	128.24	2.3	Yes	$f_{1A}/f_{1P}>f_{3A}/f_{3P}>f_{2A}/f_{2P}$	3	Yes	0.9702	0.9934	98.078%
499	644.145	644.359	1536.152	1541.028	2628.510	2628.847	0.9997	0.9968	0.9999	24.34	251.6	132.81	1.85	Yes	$f_{3A}/f_{3P}>f_{1A}/f_{1P}>f_{2A}/f_{2P}$	1	Yes	0.6446	0.9970	70.774%
500	643.659	643.952	1536.900	1538.362	2628.196	2631.068	0.9995	0.9990	0.9989	39.24	205.29	62.06	1.07	Yes	$f_{1A}/f_{1P}>f_{2A}/f_{2P}>f_{3A}/f_{3P}$	2	Yes	1.0008	0.9987	-
501	643.816	644.485	1536.196	1538.239	2625.821	2628.480	0.9990	0.9987	0.9990	35.38	331.54	53.22	1.54	Yes	$f_{3A}/f_{3P}>f_{1A}/f_{1P}>f_{2A}/f_{2P}$	1	Yes	0.6436	0.9986	70.564%
502	642.348	642.923	1534.304	1537.683	2626.836	2630.108	0.9991	0.9978	0.9988	43.37	103.05	49.76	2.05	Yes	$f_{1A}/f_{1P}>f_{3A}/f_{3P}>f_{2A}/f_{2P}$	3	Yes	0.9934	0.9978	99.637%
503	643.903	644.129	1536.776	1539.320	2628.524	2630.529	0.9996	0.9983	0.9992	82.96	229.04	56.22	0.86	Yes	$f_{1A}/f_{1P}>f_{3A}/f_{3P}>f_{2A}/f_{2P}$	3	Yes	0.9998	0.9980	99.849%
504	644.112	645.285	1535.351	1540.113	2622.922	2628.162	0.9982	0.9969	0.9980	43.98	320.57	101.55	1.55	Yes	$f_{1A}/f_{1P}>f_{3A}/f_{3P}>f_{2A}/f_{2P}$	3	Yes	0.9827	0.9965	98.852%
505	643.825	644.403	1536.746	1541.037	2628.930	2632.732	0.9991	0.9972	0.9986	33.58	220.45	132.18	1.6	Yes	$f_{1A}/f_{1P}>f_{3A}/f_{3P}>f_{2A}/f_{2P}$	3	Yes	0.9934	0.9965	99.747%
506	643.546	645.024	1536.363	1536.793	2625.795	2629.779	0.9977	0.9997	0.9985	47.94	397.98	69.15	1.18	Yes	$f_{2A}/f_{2P}>f_{3A}/f_{3P}>f_{1A}/f_{1P}$	5	Yes	0.9978	0.9980	99.980%
507	644.234	644.414	1535.185	1541.762	2626.335	2628.661	0.9997	0.9957	0.9991	28.64	260.66	94.62	2.44	Yes	$f_{1A}/f_{1P}>f_{3A}/f_{3P}>f_{2A}/f_{2P}$	3	Yes	1.0006	0.9967	-
508	640.141	641.149	1530.115	1537.907	2626.280	2630.452	0.9984	0.9949	0.9984	78.14	111.33	70.46	2.4	Yes	$f_{1A}/f_{1P}>f_{3A}/f_{3P}>f_{2A}/f_{2P}$	3	Yes	0.9856	0.9935	99.344%
509	643.712	644.230	1536.741	1537.220	2627.863	2627.927	0.9992	0.9997	1.0000	42.49	363	53.82	0.75	Yes	$f_{3A}/f_{3P}>f_{2A}/f_{2P}>f_{1A}/f_{1P}$	4	Yes	0.9999	0.9991	99.937%
510	642.529	643.067	1535.748	1538.199	2626.454	2634.665	0.9992	0.9984	0.9969	69.53	147.28	45.65	2.58	Yes	$f_{1A}/f_{1P}>f_{2A}/f_{2P}>f_{3A}/f_{3P}$	2	Yes	0.9990	0.9960	99.750%
511	638.716	640.312	1528.699	1536.506	2624.880	2630.163	0.9975	0.9949	0.9980	88.62	108.37	110.75	1.93	Yes	$f_{3A}/f_{3P}>f_{1A}/f_{1P}>f_{2A}/f_{2P}$	1	Yes	0.6415	0.9906	71.055%
512	642.553	648.269	1533.640	1535.549	2616.685	2631.453	0.9912	0.9988	0.9944	65.54	399.1	88.24	2.5	Yes	$f_{2A}/f_{2P}>f_{3A}/f_{3P}>f_{1A}/f_{1P}$	5	Yes	0.9916	0.9929	99.896%
513	644.153	644.227	1535.990	1540.278	2627.472	2628.805	0.9999	0.9972	0.9995	27.18	261.25	90.82	1.77	Yes	$f_{1A}/f_{1P}>f_{3A}/f_{3P}>f_{2A}/f_{2P}$	3	Yes	1.0025	0.9978	-
514	643.792	647.963	1534.971	1535.789	2621.021	2631.623	0.9936	0.9995	0.9960	82.82	399.96	126.35	1.4	Yes	$f_{2A}/f_{2P}>f_{3A}/f_{3P}>f_{1A}/f_{1P}$	5	Yes	0.9939	0.9927	99.899%
515	644.401	644.441	1535.759	1541.702	2627.079	2628.848	0.9999	0.9961	0.9993	28.5	263.05	133.72	1.85	Yes	$f_{1A}/f_{1P}>f_{3A}/f_{3P}>f_{2A}/f_{2P}$	3	Yes	1.0031	0.9965	-
516	645.461	645.780	1533.339	1547.875	2624.480	2627.802	0.9995	0.9906	0.9987	72.65	267.41	134.15	2.05	Yes	$f_{1A}/f_{1P}>f_{3A}/f_{3P}>f_{2A}/f_{2P}$	3	Yes	0.9981	0.9901	99.335%
517	643.654	644.126	1536.696	1537.027	2627.543	2629.310	0.9993	0.9998	0.9993	23.66	416.7	54.19	0.87	Yes	$f_{2A}/f_{2P}>f_{3A}/f_{3P}>f_{1A}/f_{1P}$	5	Yes	0.9993	0.9994	99.988%
518	643.845	644.141	1536.439	1538.191	2626.913	2628.415	0.9995	0.9989	0.9994	25.59	307.24	121.18	0.74	Yes	$f_{1A}/f_{1P}>f_{3A}/f_{3P}>f_{2A}/f_{2P}$	3	Yes	0.9985	0.9988	99.971%
519	645.063	645.417	1533.734	1545.965	2623.740	2628.248	0.9995	0.9921	0.9983	55.8	271.67	131.93	2.06	Yes	$f_{1A}/f_{1P}>f_{3A}/f_{3P}>f_{2A}/f_{2P}$	3	Yes	0.9974	0.9925	99.591%
520	644.195	644.818	1535.402	1540.561	2623.790	2628.803	0.9990	0.9967	0.9981	38.14	299.7	96.98	1.61	Yes	$f_{1A}/f_{1P}>f_{3A}/f_{3P}>f_{2A}/f_{2P}$	3	Yes	0.9926	0.9970	99.633%
521	644.209	645.047	1536.013	1539.469	2624.805	2627.631	0.9987	0.9978	0.9989	78.51	318.54	125.59	0.69	Yes	$f_{3A}/f_{3P}>f_{1A}/f_{1P}>f_{2A}/f_{2P}$	1	Yes	0.6432	0.9966	70.698%

522	643.869	646.316	1535.739	1536.522	2624.316	2631.434	0.9962	0.9995	0.9973	79.26	409.21	133.9	0.82	Yes	$f_{2A}/f_{2P}>f_{3A}/f_{3P}>f_{1A}/f_{1P}$	5	Yes	0.9964	0.9956	99.936%
523	644.131	644.175	1535.478	1540.020	2626.118	2628.794	0.9999	0.9971	0.9990	29.7	271.56	60.55	2.2	Yes	$f_{1A}/f_{1P}>f_{3A}/f_{3P}>f_{2A}/f_{2P}$	3	Yes	1.0030	0.9980	-
524	642.606	647.394	1534.611	1535.169	2617.847	2625.934	0.9926	0.9996	0.9969	79.11	382.25	66.44	2.52	Yes	$f_{2A}/f_{2P}>f_{3A}/f_{3P}>f_{1A}/f_{1P}$	5	No	-	0.9935	-
525	644.240	644.564	1533.967	1542.660	2627.770	2627.846	0.9995	0.9944	1.0000	51.44	252.11	66.89	2.36	Yes	$f_{3A}/f_{3P}>f_{1A}/f_{1P}>f_{2A}/f_{2P}$	1	Yes	0.6443	0.9960	70.841%
526	643.630	645.335	1536.411	1536.604	2625.438	2628.381	0.9974	0.9999	0.9989	73.28	383.21	64.05	1.19	Yes	$f_{2A}/f_{2P}>f_{3A}/f_{3P}>f_{1A}/f_{1P}$	5	Yes	0.9975	0.9972	99.975%
527	643.518	644.661	1536.641	1542.901	2628.849	2636.975	0.9982	0.9959	0.9969	45.37	210.5	132.72	2.08	Yes	$f_{1A}/f_{1P}>f_{3A}/f_{3P}>f_{2A}/f_{2P}$	3	Yes	0.9832	0.9938	99.123%
528	643.640	645.647	1535.821	1536.620	2624.465	2630.752	0.9969	0.9995	0.9976	83.47	404.87	69.56	1.12	Yes	$f_{2A}/f_{2P}>f_{3A}/f_{3P}>f_{1A}/f_{1P}$	5	Yes	0.9971	0.9967	99.970%
529	644.359	644.466	1535.658	1541.556	2628.069	2628.347	0.9998	0.9962	0.9999	80.99	255.64	78.38	1.22	Yes	$f_{3A}/f_{3P}>f_{1A}/f_{1P}>f_{2A}/f_{2P}$	1	Yes	0.6448	0.9961	70.869%
530	643.032	644.035	1537.726	1538.794	2628.747	2636.094	0.9984	0.9993	0.9972	30.64	180.75	111.1	2.31	Yes	$f_{2A}/f_{2P}>f_{1A}/f_{1P}>f_{3A}/f_{3P}$	2	Yes	0.9956	0.9961	99.958%
531	642.556	647.301	1533.663	1535.801	2616.791	2632.182	0.9927	0.9986	0.9942	87.15	406.65	61.54	2.34	Yes	$f_{2A}/f_{2P}>f_{3A}/f_{3P}>f_{1A}/f_{1P}$	5	Yes	0.9930	0.9938	99.935%
532	644.257	645.278	1534.918	1541.246	2620.987	2628.193	0.9984	0.9959	0.9973	29.91	307.8	120.72	2.29	Yes	$f_{1A}/f_{1P}>f_{3A}/f_{3P}>f_{2A}/f_{2P}$	3	Yes	0.9854	0.9959	99.129%
533	643.501	645.419	1535.671	1537.197	2624.843	2632.265	0.9970	0.9990	0.9972	35.1	427.42	85.73	1.5	Yes	$f_{2A}/f_{2P}>f_{3A}/f_{3P}>f_{1A}/f_{1P}$	5	Yes	0.9972	0.9977	99.955%
534	643.998	644.378	1536.138	1541.170	2628.430	2631.055	0.9994	0.9967	0.9990	59.3	234.99	97.03	1.19	Yes	$f_{1A}/f_{1P}>f_{3A}/f_{3P}>f_{2A}/f_{2P}$	3	Yes	0.9970	0.9966	99.964%
535	644.224	644.434	1536.079	1540.269	2626.822	2628.670	0.9997	0.9973	0.9993	54.38	277.13	122.54	0.8	Yes	$f_{1A}/f_{1P}>f_{3A}/f_{3P}>f_{2A}/f_{2P}$	3	Yes	1.0000	0.9973	-
536	642.882	643.164	1537.091	1538.256	2630.122	2634.330	0.9996	0.9992	0.9984	63.94	150.09	117.64	1.13	Yes	$f_{1A}/f_{1P}>f_{2A}/f_{2P}>f_{3A}/f_{3P}$	2	Yes	1.0009	0.9957	-
537	644.315	645.847	1535.758	1539.254	2623.952	2626.370	0.9976	0.9977	0.9991	84.71	337.98	126.8	0.89	Yes	$f_{3A}/f_{3P}>f_{2A}/f_{2P}>f_{1A}/f_{1P}$	4	Yes	0.9978	0.9952	99.784%
538	643.906	645.969	1534.456	1539.992	2619.266	2626.628	0.9968	0.9964	0.9972	37.78	333.13	101.12	2.47	Yes	$f_{3A}/f_{3P}>f_{1A}/f_{1P}>f_{2A}/f_{2P}$	1	Yes	0.6405	0.9953	70.577%
539	643.796	644.294	1536.698	1540.677	2628.612	2632.897	0.9992	0.9974	0.9984	54.26	218.42	112.52	1.11	Yes	$f_{1A}/f_{1P}>f_{3A}/f_{3P}>f_{2A}/f_{2P}$	3	Yes	0.9949	0.9966	99.859%
540	639.331	641.491	1529.769	1535.478	2621.924	2629.844	0.9966	0.9963	0.9970	59.8	91.93	133.84	1.58	Yes	$f_{3A}/f_{3P}>f_{1A}/f_{1P}>f_{2A}/f_{2P}$	1	Yes	0.6402	0.9937	70.689%
541	643.624	646.367	1535.649	1537.345	2624.354	2624.696	0.9958	0.9989	0.9999	33.13	365.47	111.95	2.39	Yes	$f_{3A}/f_{3P}>f_{2A}/f_{2P}>f_{1A}/f_{1P}$	4	Yes	0.9953	0.9956	99.974%
542	644.006	644.829	1536.056	1538.698	2625.128	2627.679	0.9987	0.9983	0.9990	73.09	328.22	101.15	0.71	Yes	$f_{3A}/f_{3P}>f_{1A}/f_{1P}>f_{2A}/f_{2P}$	1	Yes	0.6432	0.9974	70.635%
543	643.605	644.618	1537.002	1542.521	2628.808	2637.819	0.9984	0.9964	0.9966	59.51	206.19	117.81	1.7	Yes	$f_{1A}/f_{1P}>f_{3A}/f_{3P}>f_{2A}/f_{2P}$	3	Yes	0.9856	0.9941	99.299%
544	637.483	640.368	1525.741	1534.684	2617.696	2629.669	0.9955	0.9942	0.9954	69.06	90.17	113.59	2.11	Yes	$f_{1A}/f_{1P}>f_{3A}/f_{3P}>f_{2A}/f_{2P}$	3	No	-	0.9918	-
545	642.689	646.747	1534.824	1535.851	2619.699	2625.576	0.9937	0.9993	0.9978	72.95	378.4	58.62	2.51	Yes	$f_{2A}/f_{2P}>f_{3A}/f_{3P}>f_{1A}/f_{1P}$	5	Yes	0.9940	0.9947	99.943%
546	642.405	642.687	1535.870	1538.682	2630.483	2634.507	0.9996	0.9982	0.9985	55.63	143.04	119.8	1.7	Yes	$f_{1A}/f_{1P}>f_{3A}/f_{3P}>f_{2A}/f_{2P}$	3	Yes	0.9987	0.9944	99.636%
547	643.870	644.386	1536.872	1541.187	2628.863	2633.689	0.9992	0.9972	0.9982	69.45	217.98	111.15	1.04	Yes	$f_{1A}/f_{1P}>f_{3A}/f_{3P}>f_{2A}/f_{2P}$	3	Yes	0.9945	0.9960	99.878%
548	643.726	644.244	1536.834	1540.512	2628.729	2633.247	0.9992	0.9976	0.9983	46.53	213.17	125.87	1.17	Yes	$f_{1A}/f_{1P}>f_{3A}/f_{3P}>f_{2A}/f_{2P}$	3	Yes	0.9945	0.9966	99.829%
549	643.822	643.876	1536.648	1538.217	2628.466	2628.571	0.9999	0.9990	1.0000	25.28	249.74	96.86	0.72	Yes	$f_{3A}/f_{3P}>f_{1A}/f_{1P}>f_{2A}/f_{2P}$	1	Yes	0.6449	0.9991	70.631%
550	643.577	645.833	1535.273	1536.993	2624.178	2633.269	0.9965	0.9989	0.9965	62.47	449.33	104.28	0.96	Yes	$f_{2A}/f_{2P}>f_{3A}/f_{3P}>f_{1A}/f_{1P}$	5	Yes	0.9967	0.9969	99.987%
551	643.492	644.029	1537.374	1539.243	2628.688	2634.349	0.9992	0.9988	0.9979	60.63	191.12	94.81	1.04	Yes	$f_{1A}/f_{1P}>f_{2A}/f_{2P}>f_{3A}/f_{3P}$	2	Yes	0.9990	0.9970	99.834%
552	643.798	646.461	1536.085	1536.326	2624.028	2630.276	0.9959	0.9998	0.9976	73.63	395.32	123.45	1.01	Yes	$f_{2A}/f_{2P}>f_{3A}/f_{3P}>f_{1A}/f_{1P}$	5	Yes	0.9961	0.9954	99.941%

553	643.257	646.824	1534.678	1536.802	2621.441	2633.883	0.9945	0.9986	0.9953	62.35	416.09	86.81	1.7	Yes	$f_{2A}/f_{2P}>f_{3A}/f_{3P}>f_{1A}/f_{1P}$	5	Yes	0.9947	0.9954	99.941%
554	640.602	641.699	1532.233	1537.076	2627.507	2630.529	0.9983	0.9968	0.9989	73.34	110.25	122.96	1.39	Yes	$f_{3A}/f_{3P}>f_{1A}/f_{1P}>f_{2A}/f_{2P}$	1	Yes	0.6426	0.9937	70.885%
555	643.452	645.448	1535.810	1536.789	2624.448	2630.983	0.9969	0.9994	0.9975	60.78	407.75	67.69	1.29	Yes	$f_{2A}/f_{2P}>f_{3A}/f_{3P}>f_{1A}/f_{1P}$	5	Yes	0.9971	0.9973	99.980%
556	644.284	644.389	1536.041	1541.390	2628.713	2629.038	0.9998	0.9965	0.9999	53.33	251.95	130.65	1.02	Yes	$f_{3A}/f_{3P}>f_{1A}/f_{1P}>f_{2A}/f_{2P}$	1	Yes	0.6448	0.9964	70.843%
557	644.235	644.500	1534.032	1542.492	2626.974	2628.036	0.9996	0.9945	0.9996	44.21	255.3	69.89	2.49	Yes	$f_{3A}/f_{3P}>f_{1A}/f_{1P}>f_{2A}/f_{2P}$	1	Yes	0.6444	0.9962	70.834%
558	642.520	642.670	1534.604	1538.295	2629.535	2630.291	0.9998	0.9976	0.9997	59.67	123.9	49.64	2.17	Yes	$f_{1A}/f_{1P}>f_{3A}/f_{3P}>f_{2A}/f_{2P}$	3	Yes	1.0011	0.9968	-
559	641.007	641.306	1533.421	1538.941	2629.141	2629.670	0.9995	0.9964	0.9998	86.02	138.57	100.25	2.25	Yes	$f_{3A}/f_{3P}>f_{1A}/f_{1P}>f_{2A}/f_{2P}$	1	Yes	0.6444	0.9904	71.312%
560	643.514	643.966	1537.425	1538.719	2628.491	2634.109	0.9993	0.9992	0.9979	86.18	185.21	51.39	1.21	Yes	$f_{1A}/f_{1P}>f_{2A}/f_{2P}>f_{3A}/f_{3P}$	2	Yes	0.9996	0.9973	99.811%
561	642.911	643.099	1536.361	1538.137	2629.859	2632.757	0.9997	0.9988	0.9989	58.09	141.28	96.66	1.19	Yes	$f_{1A}/f_{1P}>f_{3A}/f_{3P}>f_{2A}/f_{2P}$	3	Yes	1.0004	0.9967	-
562	643.892	644.134	1536.431	1539.523	2628.341	2630.185	0.9996	0.9980	0.9993	58.08	233.68	64.73	1.03	Yes	$f_{1A}/f_{1P}>f_{3A}/f_{3P}>f_{2A}/f_{2P}$	3	Yes	0.9995	0.9981	99.880%
563	644.066	644.139	1536.333	1539.695	2628.549	2628.647	0.9999	0.9978	1.0000	49.58	254.13	91.53	0.86	Yes	$f_{3A}/f_{3P}>f_{1A}/f_{1P}>f_{2A}/f_{2P}$	1	Yes	0.6449	0.9980	70.716%
564	643.538	644.464	1536.202	1537.152	2626.341	2630.518	0.9986	0.9994	0.9984	28.45	443.2	49.63	1.42	Yes	$f_{2A}/f_{2P}>f_{1A}/f_{1P}>f_{3A}/f_{3P}$	2	No	-	0.9990	-
565	644.759	645.005	1535.569	1544.094	2628.641	2629.558	0.9996	0.9945	0.9997	86.22	250.76	96.66	1.48	Yes	$f_{3A}/f_{3P}>f_{1A}/f_{1P}>f_{2A}/f_{2P}$	1	Yes	0.6445	0.9938	71.033%
566	643.922	645.063	1535.882	1544.917	2627.809	2638.721	0.9982	0.9942	0.9959	83.08	218.64	97.85	1.99	Yes	$f_{1A}/f_{1P}>f_{3A}/f_{3P}>f_{2A}/f_{2P}$	3	Yes	0.9833	0.9920	99.282%
567	643.666	645.599	1535.938	1537.124	2625.106	2626.312	0.9970	0.9992	0.9995	84.56	369.48	53.9	1.54	Yes	$f_{3A}/f_{3P}>f_{2A}/f_{2P}>f_{1A}/f_{1P}$	4	Yes	0.9969	0.9965	99.966%
568	643.536	645.017	1535.914	1537.038	2625.482	2631.390	0.9977	0.9993	0.9978	47.57	427.33	76.35	1.02	Yes	$f_{2A}/f_{2P}>f_{3A}/f_{3P}>f_{1A}/f_{1P}$	5	Yes	0.9978	0.9981	99.972%
569	643.634	645.022	1536.408	1536.707	2626.053	2629.555	0.9978	0.9998	0.9987	56.07	396.28	91.02	0.82	Yes	$f_{2A}/f_{2P}>f_{3A}/f_{3P}>f_{1A}/f_{1P}$	5	Yes	0.9980	0.9979	99.991%
570	642.163	642.529	1533.873	1538.264	2628.998	2630.174	0.9994	0.9971	0.9996	57.84	116.99	56.77	2.13	Yes	$f_{3A}/f_{3P}>f_{1A}/f_{1P}>f_{2A}/f_{2P}$	1	Yes	0.6442	0.9965	70.785%
571	643.433	645.434	1536.298	1536.763	2625.288	2627.492	0.9969	0.9997	0.9992	38.68	377.37	66.55	2.06	Yes	$f_{2A}/f_{2P}>f_{3A}/f_{3P}>f_{1A}/f_{1P}$	5	Yes	0.9971	0.9974	99.975%
572	643.658	644.495	1536.800	1536.870	2627.265	2628.481	0.9987	1.0000	0.9995	26.65	382.3	75.05	1.18	Yes	$f_{2A}/f_{2P}>f_{3A}/f_{3P}>f_{1A}/f_{1P}$	5	No	-	0.9988	-
573	643.590	645.108	1535.848	1537.216	2625.735	2631.808	0.9976	0.9991	0.9977	23.64	446.27	100.84	1.5	Yes	$f_{2A}/f_{2P}>f_{3A}/f_{3P}>f_{1A}/f_{1P}$	5	Yes	0.9978	0.9982	99.966%
574	643.711	646.802	1534.752	1538.417	2622.244	2624.080	0.9952	0.9976	0.9993	63.29	355.67	94.43	1.92	Yes	$f_{3A}/f_{3P}>f_{2A}/f_{2P}>f_{1A}/f_{1P}$	4	Yes	0.9946	0.9943	99.975%
575	643.269	643.659	1537.056	1537.911	2629.134	2632.546	0.9994	0.9994	0.9987	27.17	155.21	89.09	1.74	Yes	$f_{2A}/f_{2P}>f_{1A}/f_{1P}>f_{3A}/f_{3P}$	2	No	-	0.9979	-
576	643.530	644.138	1537.209	1539.147	2628.467	2633.471	0.9991	0.9987	0.9981	27.63	197.15	95.84	1.82	Yes	$f_{1A}/f_{1P}>f_{2A}/f_{2P}>f_{3A}/f_{3P}$	2	Yes	0.9985	0.9976	99.925%
577	643.219	647.355	1534.623	1536.442	2620.711	2633.062	0.9936	0.9988	0.9953	60.96	407	98.22	1.84	Yes	$f_{2A}/f_{2P}>f_{3A}/f_{3P}>f_{1A}/f_{1P}$	5	Yes	0.9939	0.9945	99.949%
578	643.961	645.605	1535.457	1538.477	2623.440	2626.227	0.9975	0.9980	0.9989	87.07	345.82	63.39	1.44	Yes	$f_{3A}/f_{3P}>f_{2A}/f_{2P}>f_{1A}/f_{1P}$	4	Yes	0.9975	0.9960	99.878%
579	643.659	643.870	1537.112	1537.942	2628.507	2630.578	0.9997	0.9995	0.9992	30.78	196.84	91.34	0.72	Yes	$f_{1A}/f_{1P}>f_{2A}/f_{2P}>f_{3A}/f_{3P}$	2	Yes	1.0014	0.9990	-
580	644.603	644.789	1534.117	1544.218	2626.367	2628.447	0.9997	0.9935	0.9992	47.62	259.26	101.43	2.23	Yes	$f_{1A}/f_{1P}>f_{3A}/f_{3P}>f_{2A}/f_{2P}$	3	Yes	1.0005	0.9947	-
581	644.229	644.290	1536.288	1540.183	2627.741	2628.621	0.9999	0.9975	0.9997	66.13	265.6	120.44	0.67	Yes	$f_{1A}/f_{1P}>f_{3A}/f_{3P}>f_{2A}/f_{2P}$	3	Yes	1.0027	0.9973	-
582	643.544	643.552	1536.734	1537.340	2628.910	2629.231	1.0000	0.9996	0.9999	42.29	127.72	48.72	0.65	Yes	$f_{1A}/f_{1P}>f_{3A}/f_{3P}>f_{2A}/f_{2P}$	3	Yes	1.0037	0.9993	-
583	643.829	643.910	1536.675	1538.239	2628.475	2628.809	0.9999	0.9990	0.9999	45.06	244.92	55.72	0.69	Yes	$f_{1A}/f_{1P}>f_{3A}/f_{3P}>f_{2A}/f_{2P}$	3	Yes	1.0024	0.9991	-

584	644.047	646.047	1535.221	1538.836	2622.767	2625.695	0.9969	0.9977	0.9989	78.69	345.49	95.67	1.31	Yes	$f_{3A}/f_{3P}>f_{2A}/f_{2P}>f_{1A}/f_{1P}$	4	Yes	0.9968	0.9951	99.856%
585	643.755	645.412	1536.084	1537.889	2625.859	2626.720	0.9974	0.9988	0.9997	42.6	358.26	89.6	1.5	Yes	$f_{3A}/f_{3P}>f_{2A}/f_{2P}>f_{1A}/f_{1P}$	4	Yes	0.9975	0.9971	99.970%
586	644.895	645.159	1535.246	1545.346	2628.772	2629.844	0.9996	0.9935	0.9996	77.13	250.95	117.65	1.59	Yes	$f_{3A}/f_{3P}>f_{1A}/f_{1P}>f_{2A}/f_{2P}$	1	Yes	0.6445	0.9928	71.116%
587	643.886	644.060	1536.503	1539.150	2628.471	2629.462	0.9997	0.9983	0.9996	43.78	239.52	76.49	0.93	Yes	$f_{1A}/f_{1P}>f_{3A}/f_{3P}>f_{2A}/f_{2P}$	3	Yes	1.0007	0.9984	-
588	643.344	643.980	1537.709	1538.878	2628.486	2635.828	0.9990	0.9992	0.9972	73.43	178.8	66.42	1.4	Yes	$f_{2A}/f_{2P}>f_{1A}/f_{1P}>f_{3A}/f_{3P}$	2	Yes	0.9983	0.9966	99.858%
589	643.782	644.606	1536.484	1537.229	2627.210	2627.226	0.9987	0.9995	1.0000	79.31	363.24	63.51	0.7	Yes	$f_{3A}/f_{3P}>f_{2A}/f_{2P}>f_{1A}/f_{1P}$	4	Yes	0.9992	0.9982	99.919%
590	642.205	643.170	1536.558	1538.587	2627.359	2636.903	0.9985	0.9987	0.9964	69.01	155.66	73.53	2.44	Yes	$f_{2A}/f_{2P}>f_{1A}/f_{1P}>f_{3A}/f_{3P}$	2	No	-	0.9939	-
591	643.354	645.699	1535.334	1536.974	2623.527	2632.511	0.9964	0.9989	0.9966	58.06	421.21	69.55	1.44	Yes	$f_{2A}/f_{2P}>f_{3A}/f_{3P}>f_{1A}/f_{1P}$	5	Yes	0.9966	0.9971	99.959%
592	643.394	647.858	1534.103	1536.717	2620.859	2635.720	0.9931	0.9983	0.9944	55.19	426.57	132.77	1.64	Yes	$f_{2A}/f_{2P}>f_{3A}/f_{3P}>f_{1A}/f_{1P}$	5	Yes	0.9934	0.9940	99.950%
593	643.602	646.548	1534.484	1538.670	2620.250	2624.553	0.9954	0.9973	0.9984	42.47	350.15	99.38	2.51	Yes	$f_{3A}/f_{3P}>f_{2A}/f_{2P}>f_{1A}/f_{1P}$	4	Yes	0.9949	0.9948	99.989%
594	643.360	644.153	1537.796	1540.763	2629.499	2637.662	0.9988	0.9981	0.9969	75.1	190.47	118.13	1.25	Yes	$f_{1A}/f_{1P}>f_{2A}/f_{2P}>f_{3A}/f_{3P}$	2	Yes	0.9971	0.9944	99.780%
595	644.351	644.403	1535.019	1541.388	2627.393	2628.365	0.9999	0.9959	0.9996	65.99	259.29	59.12	1.66	Yes	$f_{1A}/f_{1P}>f_{3A}/f_{3P}>f_{2A}/f_{2P}$	3	Yes	1.0029	0.9968	-
596	643.368	645.900	1534.828	1537.163	2623.672	2633.134	0.9961	0.9985	0.9964	26.71	429.07	96.26	2.26	Yes	$f_{2A}/f_{2P}>f_{3A}/f_{3P}>f_{1A}/f_{1P}$	5	Yes	0.9963	0.9971	99.932%
597	642.512	643.258	1536.509	1538.609	2629.112	2636.040	0.9988	0.9986	0.9974	49.86	153.04	91.55	2.18	Yes	$f_{1A}/f_{1P}>f_{2A}/f_{2P}>f_{3A}/f_{3P}$	2	Yes	0.9974	0.9951	99.803%
598	644.058	644.480	1535.786	1539.628	2624.567	2628.910	0.9993	0.9975	0.9983	26.46	299.57	82.3	1.9	Yes	$f_{1A}/f_{1P}>f_{3A}/f_{3P}>f_{2A}/f_{2P}$	3	Yes	0.9962	0.9979	99.856%
599	644.007	644.595	1535.604	1542.389	2628.340	2631.204	0.9991	0.9956	0.9989	38.86	236.37	102.68	2.06	Yes	$f_{1A}/f_{1P}>f_{3A}/f_{3P}>f_{2A}/f_{2P}$	3	Yes	0.9932	0.9959	99.775%
600	643.674	644.816	1536.266	1537.667	2626.200	2627.447	0.9982	0.9991	0.9995	33.98	355.6	58.58	1.83	Yes	$f_{3A}/f_{3P}>f_{2A}/f_{2P}>f_{1A}/f_{1P}$	4	Yes	0.9986	0.9982	99.966%
601	643.708	644.072	1536.834	1539.096	2628.350	2631.591	0.9994	0.9985	0.9988	37.68	212.62	85.87	1.14	Yes	$f_{1A}/f_{1P}>f_{3A}/f_{3P}>f_{2A}/f_{2P}$	3	Yes	0.9973	0.9982	99.929%
602	644.597	645.006	1536.051	1544.826	2629.066	2632.555	0.9994	0.9943	0.9987	80.26	239.89	133.97	1.37	Yes	$f_{1A}/f_{1P}>f_{3A}/f_{3P}>f_{2A}/f_{2P}$	3	Yes	0.9965	0.9926	99.679%
603	643.010	646.661	1534.347	1536.809	2621.174	2633.120	0.9944	0.9984	0.9955	39.79	413.24	86.02	2.42	Yes	$f_{2A}/f_{2P}>f_{3A}/f_{3P}>f_{1A}/f_{1P}$	5	Yes	0.9946	0.9959	99.892%
604	642.909	644.068	1538.639	1541.646	2630.119	2639.752	0.9982	0.9980	0.9964	89.72	181.87	116.54	1.73	Yes	$f_{1A}/f_{1P}>f_{2A}/f_{2P}>f_{3A}/f_{3P}$	2	Yes	0.9944	0.9910	99.717%
605	645.319	645.845	1533.938	1546.530	2623.620	2627.950	0.9992	0.9919	0.9984	76.97	273.56	128.87	1.79	Yes	$f_{1A}/f_{1P}>f_{3A}/f_{3P}>f_{2A}/f_{2P}$	3	Yes	0.9944	0.9912	99.731%
606	643.683	644.343	1537.056	1541.051	2628.822	2635.156	0.9990	0.9974	0.9976	66.02	207.9	110.52	1.18	Yes	$f_{1A}/f_{1P}>f_{3A}/f_{3P}>f_{2A}/f_{2P}$	3	Yes	0.9919	0.9957	99.686%
607	641.729	642.342	1532.724	1537.393	2626.419	2630.404	0.9990	0.9970	0.9985	29.82	110.32	133.66	2.01	Yes	$f_{1A}/f_{1P}>f_{3A}/f_{3P}>f_{2A}/f_{2P}$	3	Yes	0.9928	0.9960	99.729%
608	643.594	644.146	1537.162	1540.028	2628.940	2633.884	0.9991	0.9981	0.9981	45.61	202.16	132.43	1.14	Yes	$f_{1A}/f_{1P}>f_{2A}/f_{2P}>f_{3A}/f_{3P}$	2	Yes	0.9989	0.9965	99.805%
609	639.864	641.629	1529.890	1536.901	2621.599	2630.554	0.9972	0.9954	0.9966	66.93	94.26	55.25	2.53	Yes	$f_{1A}/f_{1P}>f_{3A}/f_{3P}>f_{2A}/f_{2P}$	3	Yes	0.9719	0.9954	98.050%
610	644.072	644.155	1536.089	1539.669	2626.972	2628.565	0.9999	0.9977	0.9994	24.9	271.83	128.55	1.3	Yes	$f_{1A}/f_{1P}>f_{3A}/f_{3P}>f_{2A}/f_{2P}$	3	Yes	1.0023	0.9979	-
611	643.180	649.281	1532.397	1535.712	2616.283	2637.017	0.9906	0.9978	0.9921	87.78	417.93	105.51	2.01	Yes	$f_{2A}/f_{2P}>f_{3A}/f_{3P}>f_{1A}/f_{1P}$	5	Yes	0.9910	0.9907	99.979%
612	643.482	646.694	1535.031	1537.661	2623.407	2623.707	0.9950	0.9983	0.9999	50.25	362.72	94.07	2.19	Yes	$f_{3A}/f_{3P}>f_{2A}/f_{2P}>f_{1A}/f_{1P}$	4	Yes	0.9943	0.9949	99.953%
613	643.335	646.030	1534.727	1537.059	2623.432	2632.605	0.9958	0.9985	0.9965	26.32	419.65	96.45	2.47	Yes	$f_{2A}/f_{2P}>f_{3A}/f_{3P}>f_{1A}/f_{1P}$	5	Yes	0.9960	0.9969	99.926%
614	643.752	644.922	1536.377	1537.689	2626.298	2627.439	0.9982	0.9991	0.9996	27.43	356.88	84.24	1.72	Yes	$f_{3A}/f_{3P}>f_{2A}/f_{2P}>f_{1A}/f_{1P}$	4	Yes	0.9985	0.9980	99.960%

615	643.561	644.614	1536.394	1537.165	2626.349	2630.420	0.9984	0.9995	0.9985	32.28	418.9	48.71	1.46	Yes	$f_{2A}/f_{2P}>f_{3A}/f_{3P}>f_{1A}/f_{1P}$	5	Yes	0.9985	0.9989	99.971%
616	644.224	646.788	1532.916	1541.735	2615.817	2625.343	0.9960	0.9943	0.9964	75.4	324.94	92.11	2.12	Yes	$f_{3A}/f_{3P}>f_{1A}/f_{1P}>f_{2A}/f_{2P}$	1	Yes	0.6394	0.9927	70.705%
617	643.358	648.405	1534.347	1536.045	2619.607	2633.248	0.9922	0.9989	0.9948	66.24	404.46	120.13	1.84	Yes	$f_{2A}/f_{2P}>f_{3A}/f_{3P}>f_{1A}/f_{1P}$	5	Yes	0.9926	0.9927	99.991%
618	643.840	646.268	1532.545	1541.114	2616.039	2626.029	0.9962	0.9944	0.9962	68.14	324.98	74.18	2.41	Yes	$f_{1A}/f_{1P}>f_{3A}/f_{3P}>f_{2A}/f_{2P}$	3	Yes	0.9602	0.9940	97.200%
619	644.969	645.160	1533.196	1546.072	2622.234	2628.037	0.9997	0.9917	0.9978	44.69	270.61	127.87	2.59	Yes	$f_{1A}/f_{1P}>f_{3A}/f_{3P}>f_{2A}/f_{2P}$	3	Yes	1.0004	0.9927	-
620	642.788	643.610	1538.855	1539.692	2630.597	2638.639	0.9987	0.9995	0.9970	84.52	169.54	118.74	1.5	Yes	$f_{2A}/f_{2P}>f_{1A}/f_{1P}>f_{3A}/f_{3P}$	2	Yes	0.9969	0.9925	99.634%
621	644.235	644.318	1535.761	1540.864	2627.331	2628.820	0.9999	0.9967	0.9994	31.42	261.21	93.42	1.79	Yes	$f_{1A}/f_{1P}>f_{3A}/f_{3P}>f_{2A}/f_{2P}$	3	Yes	1.0023	0.9974	-
622	643.472	645.179	1535.891	1536.967	2625.132	2631.135	0.9974	0.9993	0.9977	39.41	414.93	77.14	1.36	Yes	$f_{2A}/f_{2P}>f_{3A}/f_{3P}>f_{1A}/f_{1P}$	5	Yes	0.9975	0.9979	99.964%
623	640.662	641.540	1531.400	1537.835	2626.855	2630.252	0.9986	0.9958	0.9987	83.62	110.49	56.45	2.33	Yes	$f_{3A}/f_{3P}>f_{1A}/f_{1P}>f_{2A}/f_{2P}$	1	Yes	0.6431	0.9946	70.858%
624	638.995	641.044	1528.424	1536.322	2621.319	2630.556	0.9968	0.9949	0.9965	69.86	95.63	91.71	2.1	Yes	$f_{1A}/f_{1P}>f_{3A}/f_{3P}>f_{2A}/f_{2P}$	3	Yes	0.9667	0.9933	97.791%
625	642.193	642.914	1534.226	1536.666	2625.635	2629.337	0.9989	0.9984	0.9986	24.64	93.35	134.84	1.33	Yes	$f_{1A}/f_{1P}>f_{3A}/f_{3P}>f_{2A}/f_{2P}$	3	Yes	0.9908	0.9978	99.421%
626	642.714	642.716	1534.868	1538.430	2630.038	2631.007	1.0000	0.9977	0.9996	47.83	127.52	71.16	1.98	Yes	$f_{1A}/f_{1P}>f_{3A}/f_{3P}>f_{2A}/f_{2P}$	3	Yes	1.0038	0.9967	-
627	643.656	644.665	1536.560	1537.172	2627.223	2627.443	0.9984	0.9996	0.9999	35.05	368.09	63.26	1.39	Yes	$f_{3A}/f_{3P}>f_{2A}/f_{2P}>f_{1A}/f_{1P}$	4	Yes	0.9989	0.9985	99.963%
628	643.128	645.662	1535.414	1536.969	2623.898	2625.640	0.9961	0.9990	0.9993	59.6	369.37	49.39	2.27	Yes	$f_{3A}/f_{3P}>f_{2A}/f_{2P}>f_{1A}/f_{1P}$	4	Yes	0.9957	0.9967	99.918%
629	643.989	644.425	1536.128	1540.742	2627.952	2632.063	0.9993	0.9970	0.9984	82.38	227.14	51.67	1.53	Yes	$f_{1A}/f_{1P}>f_{3A}/f_{3P}>f_{2A}/f_{2P}$	3	Yes	0.9960	0.9967	99.938%
630	643.301	646.670	1535.297	1536.564	2622.444	2631.385	0.9948	0.9992	0.9966	46.37	401.06	96.79	1.93	Yes	$f_{2A}/f_{2P}>f_{3A}/f_{3P}>f_{1A}/f_{1P}$	5	Yes	0.9950	0.9957	99.943%
631	644.160	645.056	1535.066	1540.591	2622.875	2628.144	0.9986	0.9964	0.9980	53.18	307.05	103.08	1.34	Yes	$f_{1A}/f_{1P}>f_{3A}/f_{3P}>f_{2A}/f_{2P}$	3	Yes	0.9877	0.9963	99.285%
632	644.584	645.196	1534.400	1543.178	2622.692	2628.590	0.9991	0.9943	0.9978	48.06	285.16	114.16	1.85	Yes	$f_{1A}/f_{1P}>f_{3A}/f_{3P}>f_{2A}/f_{2P}$	3	Yes	0.9928	0.9949	99.822%
633	643.748	645.375	1536.468	1536.638	2625.801	2629.503	0.9975	0.9999	0.9986	66.05	392.96	107.86	0.77	Yes	$f_{2A}/f_{2P}>f_{3A}/f_{3P}>f_{1A}/f_{1P}$	5	Yes	0.9976	0.9972	99.970%
634	645.072	645.793	1532.226	1546.966	2619.310	2627.414	0.9989	0.9905	0.9969	56.32	279.09	124.96	2.49	Yes	$f_{1A}/f_{1P}>f_{3A}/f_{3P}>f_{2A}/f_{2P}$	3	Yes	0.9908	0.9913	99.956%
635	644.551	645.061	1534.519	1542.460	2623.179	2628.545	0.9992	0.9949	0.9980	71.04	283.95	80.92	1.61	Yes	$f_{1A}/f_{1P}>f_{3A}/f_{3P}>f_{2A}/f_{2P}$	3	Yes	0.9946	0.9954	99.935%
636	643.964	644.811	1535.747	1539.184	2623.137	2628.435	0.9987	0.9978	0.9980	23.14	322.33	90.79	2.3	Yes	$f_{1A}/f_{1P}>f_{3A}/f_{3P}>f_{2A}/f_{2P}$	3	Yes	0.9886	0.9976	99.253%
637	643.960	644.725	1536.285	1538.120	2625.707	2627.609	0.9988	0.9988	0.9993	79.56	338.32	85.31	0.67	Yes	$f_{3A}/f_{3P}>f_{1A}/f_{1P}>f_{2A}/f_{2P}$	1	No	-	0.9977	-
638	643.674	644.208	1536.722	1539.558	2628.146	2632.420	0.9992	0.9982	0.9984	29.52	212.19	77.67	1.91	Yes	$f_{1A}/f_{1P}>f_{3A}/f_{3P}>f_{2A}/f_{2P}$	3	Yes	0.9942	0.9978	99.700%
639	643.689	644.193	1536.754	1539.699	2627.953	2633.414	0.9992	0.9981	0.9979	68.24	207.25	59.41	1.33	Yes	$f_{1A}/f_{1P}>f_{2A}/f_{2P}>f_{3A}/f_{3P}$	2	Yes	0.9992	0.9973	99.840%
640	644.248	644.402	1535.440	1541.560	2626.486	2628.761	0.9998	0.9960	0.9991	27.04	261.48	101.36	2.32	Yes	$f_{1A}/f_{1P}>f_{3A}/f_{3P}>f_{2A}/f_{2P}$	3	Yes	1.0011	0.9968	-
641	643.322	645.471	1535.263	1537.082	2623.650	2633.047	0.9967	0.9988	0.9964	56.04	449.71	64.4	1.39	Yes	$f_{2A}/f_{2P}>f_{1A}/f_{1P}>f_{3A}/f_{3P}$	2	No	-	0.9975	-
642	642.919	644.004	1537.807	1538.792	2628.152	2637.459	0.9983	0.9994	0.9965	41.15	176.78	95.09	2.28	Yes	$f_{2A}/f_{2P}>f_{1A}/f_{1P}>f_{3A}/f_{3P}$	2	Yes	0.9950	0.9956	99.952%
643	644.023	644.122	1536.499	1539.472	2628.595	2628.928	0.9998	0.9981	0.9999	65.48	249.23	77.33	0.74	Yes	$f_{3A}/f_{3P}>f_{1A}/f_{1P}>f_{2A}/f_{2P}$	1	Yes	0.6448	0.9981	70.705%
644	643.389	643.796	1537.752	1538.460	2629.323	2634.057	0.9994	0.9995	0.9982	70.89	175.28	102.95	0.8	Yes	$f_{2A}/f_{2P}>f_{1A}/f_{1P}>f_{3A}/f_{3P}$	2	Yes	0.9999	0.9971	99.765%
645	643.503	643.947	1537.297	1537.877	2628.131	2632.175	0.9993	0.9996	0.9985	22.77	181.85	58.14	2.15	Yes	$f_{2A}/f_{2P}>f_{1A}/f_{1P}>f_{3A}/f_{3P}$	2	Yes	0.9997	0.9986	99.908%

646	643.647	643.849	1536.976	1538.089	2628.421	2630.540	0.9997	0.9993	0.9992	28.77	202.5	107.11	0.75	Yes	$f_{1A}/f_{1P}>f_{2A}/f_{2P}>f_{3A}/f_{3P}$	2	Yes	1.0014	0.9988	-
647	644.413	644.499	1535.865	1541.970	2628.649	2628.692	0.9999	0.9960	1.0000	65.97	254.54	117.86	1.06	Yes	$f_{3A}/f_{3P}>f_{1A}/f_{1P}>f_{2A}/f_{2P}$	1	Yes	0.6448	0.9959	70.894%
648	644.153	644.716	1534.130	1541.222	2622.239	2628.893	0.9991	0.9954	0.9975	42.15	291.03	65.15	2.37	Yes	$f_{1A}/f_{1P}>f_{3A}/f_{3P}>f_{2A}/f_{2P}$	3	Yes	0.9937	0.9968	99.745%
649	643.971	644.107	1535.583	1539.679	2627.860	2628.352	0.9998	0.9973	0.9998	29.77	254.59	49.69	2.31	Yes	$f_{3A}/f_{3P}>f_{1A}/f_{1P}>f_{2A}/f_{2P}$	1	Yes	0.6447	0.9983	70.682%
650	643.553	646.773	1535.982	1536.370	2623.542	2629.783	0.9950	0.9997	0.9976	39.69	391.76	126.48	1.83	Yes	$f_{2A}/f_{2P}>f_{3A}/f_{3P}>f_{1A}/f_{1P}$	5	Yes	0.9953	0.9954	99.989%
651	644.476	645.299	1533.780	1546.791	2627.474	2632.603	0.9987	0.9916	0.9981	72.22	240.29	103.84	2.27	Yes	$f_{1A}/f_{1P}>f_{3A}/f_{3P}>f_{2A}/f_{2P}$	3	Yes	0.9890	0.9916	99.788%
652	643.830	644.832	1535.612	1543.941	2628.367	2634.329	0.9984	0.9946	0.9977	43.55	226.5	124.11	2.27	Yes	$f_{1A}/f_{1P}>f_{3A}/f_{3P}>f_{2A}/f_{2P}$	3	Yes	0.9858	0.9939	99.327%
653	643.705	644.614	1536.367	1537.483	2626.802	2627.456	0.9986	0.9993	0.9998	57.83	356.76	51.09	1.1	Yes	$f_{3A}/f_{3P}>f_{2A}/f_{2P}>f_{1A}/f_{1P}$	4	Yes	0.9991	0.9984	99.940%
654	644.390	645.063	1532.574	1545.421	2626.407	2629.932	0.9990	0.9917	0.9987	76.8	244.6	74.43	2.5	Yes	$f_{1A}/f_{1P}>f_{3A}/f_{3P}>f_{2A}/f_{2P}$	3	Yes	0.9917	0.9929	99.898%
655	643.837	645.564	1534.503	1539.499	2621.284	2627.016	0.9973	0.9968	0.9978	69.45	332.27	60.92	1.9	Yes	$f_{3A}/f_{3P}>f_{1A}/f_{1P}>f_{2A}/f_{2P}$	1	Yes	0.6412	0.9960	70.583%
656	642.765	644.062	1537.767	1539.892	2626.982	2640.180	0.9980	0.9986	0.9950	73.68	178.35	80.34	2.22	Yes	$f_{2A}/f_{2P}>f_{1A}/f_{1P}>f_{3A}/f_{3P}$	2	Yes	0.9934	0.9935	99.993%
657	643.023	643.533	1538.233	1538.493	2630.069	2635.995	0.9992	0.9998	0.9978	68.79	165.9	127.04	1.11	Yes	$f_{2A}/f_{2P}>f_{1A}/f_{1P}>f_{3A}/f_{3P}$	2	Yes	0.9992	0.9951	99.663%
658	643.561	643.701	1537.157	1537.361	2628.622	2630.355	0.9998	0.9999	0.9993	39.69	156.65	50.36	0.84	Yes	$f_{2A}/f_{2P}>f_{1A}/f_{1P}>f_{3A}/f_{3P}$	2	No	-	0.9992	-
659	642.076	642.796	1534.563	1536.733	2626.511	2629.634	0.9989	0.9986	0.9988	76.32	92.53	86.26	0.76	Yes	$f_{1A}/f_{1P}>f_{3A}/f_{3P}>f_{2A}/f_{2P}$	3	Yes	0.9908	0.9975	99.447%
660	643.136	643.276	1536.152	1537.483	2629.187	2629.549	0.9998	0.9991	0.9999	75.67	115.25	51.35	0.78	Yes	$f_{3A}/f_{3P}>f_{1A}/f_{1P}>f_{2A}/f_{2P}$	1	Yes	0.6447	0.9985	70.665%
661	643.498	645.411	1535.526	1537.496	2625.100	2625.429	0.9970	0.9987	0.9999	75.82	360.52	45.82	1.84	Yes	$f_{3A}/f_{3P}>f_{2A}/f_{2P}>f_{1A}/f_{1P}$	4	Yes	0.9970	0.9968	99.985%
662	643.354	645.844	1534.982	1536.972	2622.574	2633.437	0.9961	0.9987	0.9959	85.38	437.2	52.61	1.52	Yes	$f_{2A}/f_{2P}>f_{1A}/f_{1P}>f_{3A}/f_{3P}$	2	No	-	0.9966	-
663	643.634	644.963	1536.048	1537.739	2625.964	2627.077	0.9979	0.9989	0.9996	50.21	355.61	51.67	1.65	Yes	$f_{3A}/f_{3P}>f_{2A}/f_{2P}>f_{1A}/f_{1P}$	4	Yes	0.9982	0.9979	99.972%
664	643.611	644.891	1536.116	1537.052	2626.134	2631.067	0.9980	0.9994	0.9981	34.48	438.35	111.3	0.84	Yes	$f_{2A}/f_{2P}>f_{3A}/f_{3P}>f_{1A}/f_{1P}$	5	Yes	0.9981	0.9984	99.977%
665	644.251	645.423	1536.217	1538.682	2625.355	2626.879	0.9982	0.9984	0.9994	88.37	340.88	129.2	0.66	Yes	$f_{3A}/f_{3P}>f_{2A}/f_{2P}>f_{1A}/f_{1P}$	4	Yes	0.9985	0.9962	99.810%
666	643.134	648.245	1533.574	1536.403	2619.699	2634.674	0.9921	0.9982	0.9943	45.48	415.12	130.05	2.22	Yes	$f_{2A}/f_{2P}>f_{3A}/f_{3P}>f_{1A}/f_{1P}$	5	Yes	0.9925	0.9935	99.918%
667	643.878	646.075	1535.709	1536.537	2624.505	2631.328	0.9966	0.9995	0.9974	88.67	408.57	106.37	0.86	Yes	$f_{2A}/f_{2P}>f_{3A}/f_{3P}>f_{1A}/f_{1P}$	5	Yes	0.9968	0.9959	99.927%
668	644.158	645.447	1535.076	1547.571	2627.733	2639.250	0.9980	0.9919	0.9956	86.1	225.14	114.33	2.23	Yes	$f_{1A}/f_{1P}>f_{3A}/f_{3P}>f_{2A}/f_{2P}$	3	Yes	0.9806	0.9891	99.294%
669	643.769	643.980	1536.795	1538.544	2628.355	2630.297	0.9997	0.9989	0.9993	45.74	220.55	61.96	0.86	Yes	$f_{1A}/f_{1P}>f_{3A}/f_{3P}>f_{2A}/f_{2P}$	3	Yes	1.0000	0.9988	-
670	643.806	644.303	1536.499	1537.804	2626.738	2628.252	0.9992	0.9992	0.9994	45.25	336.06	70.7	0.74	Yes	$f_{3A}/f_{3P}>f_{1A}/f_{1P}>f_{2A}/f_{2P}$	1	No	-	0.9988	-
671	643.093	643.173	1535.862	1537.842	2629.549	2629.865	0.9999	0.9987	0.9999	52.08	123.15	52.39	1.39	Yes	$f_{3A}/f_{3P}>f_{1A}/f_{1P}>f_{2A}/f_{2P}$	1	Yes	0.6449	0.9981	70.713%
672	643.941	645.635	1536.386	1536.933	2625.791	2628.148	0.9974	0.9996	0.9991	80.56	378.77	115.98	0.75	Yes	$f_{2A}/f_{2P}>f_{3A}/f_{3P}>f_{1A}/f_{1P}$	5	Yes	0.9975	0.9965	99.913%
673	643.279	643.302	1536.393	1537.653	2629.413	2630.135	1.0000	0.9992	0.9997	67.84	127.52	51.12	0.92	Yes	$f_{1A}/f_{1P}>f_{3A}/f_{3P}>f_{2A}/f_{2P}$	3	Yes	1.0034	0.9984	-
674	637.390	640.064	1525.538	1535.040	2618.966	2629.728	0.9958	0.9938	0.9959	76.2	94.85	108.57	2.17	Yes	$f_{3A}/f_{3P}>f_{1A}/f_{1P}>f_{2A}/f_{2P}$	1	Yes	0.6391	0.9911	70.813%
675	643.806	644.015	1536.557	1538.778	2628.261	2629.950	0.9997	0.9986	0.9994	46.68	229.3	53.05	1.07	Yes	$f_{1A}/f_{1P}>f_{3A}/f_{3P}>f_{2A}/f_{2P}$	3	Yes	1.0001	0.9987	-
676	644.074	644.478	1535.933	1541.299	2628.181	2631.329	0.9994	0.9965	0.9988	70.71	234.52	66.4	1.46	Yes	$f_{1A}/f_{1P}>f_{3A}/f_{3P}>f_{2A}/f_{2P}$	3	Yes	0.9966	0.9966	99.998%

677	644.653	645.454	1533.564	1543.405	2620.627	2628.139	0.9988	0.9936	0.9971	74.64	288.84	83.1	1.89	Yes	$f_{1A}/f_{1P}>f_{3A}/f_{3P}>f_{2A}/f_{2P}$	3	Yes	0.9894	0.9942	99.605%
678	643.134	644.308	1537.106	1540.309	2626.844	2638.865	0.9982	0.9979	0.9954	58.94	188.6	77.12	2.2	Yes	$f_{1A}/f_{1P}>f_{2A}/f_{2P}>f_{3A}/f_{3P}$	2	Yes	0.9943	0.9950	99.939%
679	643.914	644.291	1536.389	1540.327	2628.629	2630.206	0.9994	0.9974	0.9994	25.95	235.94	101.25	1.83	Yes	$f_{1A}/f_{1P}>f_{3A}/f_{3P}>f_{2A}/f_{2P}$	3	Yes	0.9970	0.9976	99.950%
680	643.370	646.478	1534.558	1536.907	2622.408	2634.645	0.9952	0.9985	0.9954	57.99	440.53	105.25	1.33	Yes	$f_{2A}/f_{2P}>f_{3A}/f_{3P}>f_{1A}/f_{1P}$	5	Yes	0.9954	0.9959	99.955%
681	644.909	645.055	1533.228	1546.382	2624.640	2628.078	0.9998	0.9915	0.9987	48.04	262.02	122.94	2.55	Yes	$f_{1A}/f_{1P}>f_{3A}/f_{3P}>f_{2A}/f_{2P}$	3	Yes	1.0012	0.9926	-
682	641.194	642.140	1532.817	1537.532	2627.248	2630.842	0.9985	0.9969	0.9986	67.36	106.8	99.43	1.46	Yes	$f_{3A}/f_{3P}>f_{1A}/f_{1P}>f_{2A}/f_{2P}$	1	Yes	0.6429	0.9951	70.795%
683	644.131	646.821	1533.892	1541.058	2618.031	2625.338	0.9958	0.9953	0.9972	46.78	332	126.09	2.26	Yes	$f_{3A}/f_{3P}>f_{1A}/f_{1P}>f_{2A}/f_{2P}$	1	Yes	0.6391	0.9934	70.624%
684	642.975	643.257	1535.679	1537.283	2628.013	2629.429	0.9996	0.9990	0.9995	32.97	106.04	89.61	0.95	Yes	$f_{1A}/f_{1P}>f_{3A}/f_{3P}>f_{2A}/f_{2P}$	3	Yes	0.9987	0.9986	99.988%
685	642.650	648.226	1532.046	1536.339	2615.563	2638.675	0.9914	0.9972	0.9912	87.27	438.76	78.97	2.16	Yes	$f_{2A}/f_{2P}>f_{1A}/f_{1P}>f_{3A}/f_{3P}$	2	No	-	0.9926	-
686	643.561	644.182	1537.174	1540.250	2628.819	2634.720	0.9990	0.9980	0.9978	53.02	200.88	119.92	1.18	Yes	$f_{1A}/f_{1P}>f_{2A}/f_{2P}>f_{3A}/f_{3P}$	2	Yes	0.9984	0.9962	99.821%
687	642.674	649.845	1531.294	1536.027	2615.807	2638.891	0.9890	0.9969	0.9913	52.76	428.03	134.6	2.52	Yes	$f_{2A}/f_{2P}>f_{3A}/f_{3P}>f_{1A}/f_{1P}$	5	Yes	0.9894	0.9912	99.855%
688	643.718	644.306	1536.699	1540.237	2628.196	2633.429	0.9991	0.9977	0.9980	43.03	212.13	84.45	1.57	Yes	$f_{1A}/f_{1P}>f_{3A}/f_{3P}>f_{2A}/f_{2P}$	3	Yes	0.9932	0.9972	99.672%
689	644.011	645.038	1534.457	1544.854	2626.885	2635.213	0.9984	0.9933	0.9968	71.15	228.77	86.84	2.26	Yes	$f_{1A}/f_{1P}>f_{3A}/f_{3P}>f_{2A}/f_{2P}$	3	Yes	0.9853	0.9931	99.355%
690	642.856	644.060	1537.287	1538.831	2625.820	2638.352	0.9981	0.9990	0.9953	53.87	177.87	65.91	2.58	Yes	$f_{2A}/f_{2P}>f_{1A}/f_{1P}>f_{3A}/f_{3P}$	2	Yes	0.9941	0.9955	99.886%
691	644.477	644.878	1534.882	1542.257	2624.323	2628.444	0.9994	0.9952	0.9984	61.07	280.18	105.81	1.38	Yes	$f_{1A}/f_{1P}>f_{3A}/f_{3P}>f_{2A}/f_{2P}$	3	Yes	0.9966	0.9955	99.912%
692	643.524	644.372	1537.552	1540.918	2628.684	2638.019	0.9987	0.9978	0.9965	79.39	194.7	83.42	1.5	Yes	$f_{1A}/f_{1P}>f_{2A}/f_{2P}>f_{3A}/f_{3P}$	2	Yes	0.9967	0.9950	99.862%
693	643.724	643.933	1536.745	1538.287	2628.215	2630.114	0.9997	0.9990	0.9993	30.89	218.18	55.19	1.19	Yes	$f_{1A}/f_{1P}>f_{3A}/f_{3P}>f_{2A}/f_{2P}$	3	Yes	1.0001	0.9990	-
694	642.767	648.427	1532.309	1536.770	2617.535	2638.772	0.9913	0.9971	0.9920	52.67	438.43	111.12	2.29	Yes	$f_{2A}/f_{2P}>f_{3A}/f_{3P}>f_{1A}/f_{1P}$	5	Yes	0.9917	0.9934	99.862%
695	642.523	643.009	1535.412	1538.631	2629.418	2633.827	0.9992	0.9979	0.9983	45.35	142.5	82.09	2.27	Yes	$f_{1A}/f_{1P}>f_{3A}/f_{3P}>f_{2A}/f_{2P}$	3	Yes	0.9950	0.9958	99.936%
696	642.199	643.173	1535.966	1538.602	2628.945	2636.131	0.9985	0.9983	0.9973	43.96	154.03	99.12	2.55	Yes	$f_{1A}/f_{1P}>f_{2A}/f_{2P}>f_{3A}/f_{3P}$	2	Yes	0.9958	0.9945	99.896%
697	643.444	645.133	1535.717	1536.887	2624.517	2631.328	0.9974	0.9992	0.9974	77.03	422.4	46.91	1.26	Yes	$f_{2A}/f_{2P}>f_{3A}/f_{3P}>f_{1A}/f_{1P}$	5	Yes	0.9975	0.9977	99.982%
698	643.561	643.773	1537.205	1537.618	2628.580	2630.707	0.9997	0.9997	0.9992	23.07	180.36	108.91	0.87	Yes	$f_{2A}/f_{2P}>f_{1A}/f_{1P}>f_{3A}/f_{3P}$	2	Yes	1.0014	0.9989	-
699	643.200	645.865	1534.831	1537.004	2621.939	2633.759	0.9959	0.9986	0.9955	81.22	443.45	50.48	1.65	Yes	$f_{2A}/f_{2P}>f_{1A}/f_{1P}>f_{3A}/f_{3P}$	2	No	-	0.9966	-
700	644.145	644.851	1534.540	1544.238	2628.074	2630.183	0.9989	0.9937	0.9992	43.14	242.67	105.05	2.46	Yes	$f_{3A}/f_{3P}>f_{1A}/f_{1P}>f_{2A}/f_{2P}$	1	Yes	0.6435	0.9945	70.895%
701	642.859	643.435	1537.509	1538.488	2629.171	2636.542	0.9991	0.9994	0.9972	78.67	156.18	68.9	1.7	Yes	$f_{2A}/f_{2P}>f_{1A}/f_{1P}>f_{3A}/f_{3P}$	2	No	-	0.9954	-
702	643.452	643.581	1536.777	1537.513	2628.758	2630.340	0.9998	0.9995	0.9994	31.45	143.1	51.59	1.31	Yes	$f_{1A}/f_{1P}>f_{2A}/f_{2P}>f_{3A}/f_{3P}$	2	Yes	1.0020	0.9989	-
703	643.656	645.792	1535.230	1536.758	2623.974	2632.606	0.9967	0.9990	0.9967	89.72	427.17	71.45	1.09	Yes	$f_{2A}/f_{2P}>f_{3A}/f_{3P}>f_{1A}/f_{1P}$	5	Yes	0.9969	0.9965	99.967%
704	643.138	643.248	1535.602	1537.709	2628.597	2629.775	0.9998	0.9986	0.9996	22.6	114.87	83.11	1.67	Yes	$f_{1A}/f_{1P}>f_{3A}/f_{3P}>f_{2A}/f_{2P}$	3	Yes	1.0018	0.9984	-
705	643.349	645.645	1535.688	1536.758	2623.429	2631.157	0.9964	0.9993	0.9971	75.34	407.61	51.15	1.58	Yes	$f_{2A}/f_{2P}>f_{3A}/f_{3P}>f_{1A}/f_{1P}$	5	Yes	0.9966	0.9970	99.970%
706	644.017	646.051	1535.591	1538.559	2624.502	2625.864	0.9969	0.9981	0.9995	66.52	351.26	120.95	1.14	Yes	$f_{3A}/f_{3P}>f_{2A}/f_{2P}>f_{1A}/f_{1P}$	4	Yes	0.9967	0.9954	99.892%
707	644.702	645.389	1533.086	1544.017	2620.648	2628.159	0.9989	0.9929	0.9971	71.4	283.85	83.37	2.06	Yes	$f_{1A}/f_{1P}>f_{3A}/f_{3P}>f_{2A}/f_{2P}$	3	Yes	0.9915	0.9939	99.800%

708	643.769	644.651	1536.516	1536.838	2626.791	2629.396	0.9986	0.9998	0.9990	88.12	400.64	55.31	0.67	Yes	$f_{2A}/f_{2P}>f_{3A}/f_{3P}>f_{1A}/f_{1P}$	5	Yes	0.9987	0.9984	99.971%
709	643.387	643.479	1536.368	1537.360	2628.663	2629.117	0.9999	0.9994	0.9998	30.4	113.49	54.12	0.98	Yes	$f_{1A}/f_{1P}>f_{3A}/f_{3P}>f_{2A}/f_{2P}$	3	Yes	1.0022	0.9992	-
710	643.889	644.413	1535.993	1538.689	2625.422	2628.384	0.9992	0.9982	0.9989	47.44	314.04	78.44	0.96	Yes	$f_{1A}/f_{1P}>f_{3A}/f_{3P}>f_{2A}/f_{2P}$	3	Yes	0.9944	0.9982	99.684%
711	644.066	644.422	1536.145	1539.030	2625.485	2628.221	0.9994	0.9981	0.9990	84.55	300.36	64.6	0.79	Yes	$f_{1A}/f_{1P}>f_{3A}/f_{3P}>f_{2A}/f_{2P}$	3	Yes	0.9974	0.9978	99.964%
712	643.905	644.022	1536.552	1538.894	2628.446	2629.111	0.9998	0.9985	0.9997	67.4	243.39	56.07	0.77	Yes	$f_{1A}/f_{1P}>f_{3A}/f_{3P}>f_{2A}/f_{2P}$	3	Yes	1.0017	0.9985	-
713	643.067	643.106	1536.442	1537.938	2630.085	2631.655	0.9999	0.9990	0.9994	86.59	133.17	70.76	0.89	Yes	$f_{1A}/f_{1P}>f_{3A}/f_{3P}>f_{2A}/f_{2P}$	3	Yes	1.0031	0.9973	-
714	644.529	648.968	1533.217	1540.103	2617.033	2620.400	0.9932	0.9955	0.9987	87.01	347.22	130.45	1.98	Yes	$f_{3A}/f_{3P}>f_{2A}/f_{2P}>f_{1A}/f_{1P}$	4	Yes	0.9918	0.9888	99.754%
715	644.284	644.352	1535.402	1541.429	2627.555	2628.487	0.9999	0.9961	0.9996	50.34	258.88	94.7	1.4	Yes	$f_{1A}/f_{1P}>f_{3A}/f_{3P}>f_{2A}/f_{2P}$	3	Yes	1.0026	0.9967	-
716	643.284	647.286	1533.844	1536.949	2621.583	2635.585	0.9938	0.9980	0.9947	36.12	442.99	133.15	2.04	Yes	$f_{2A}/f_{2P}>f_{3A}/f_{3P}>f_{1A}/f_{1P}$	5	Yes	0.9941	0.9951	99.915%
717	643.728	645.228	1535.723	1537.973	2624.896	2626.626	0.9977	0.9985	0.9993	74.36	351.7	50.52	1.55	Yes	$f_{3A}/f_{3P}>f_{2A}/f_{2P}>f_{1A}/f_{1P}$	4	Yes	0.9978	0.9971	99.941%
718	644.231	644.783	1533.839	1541.108	2622.741	2628.309	0.9991	0.9953	0.9979	78.52	287.85	47.31	2	Yes	$f_{1A}/f_{1P}>f_{3A}/f_{3P}>f_{2A}/f_{2P}$	3	Yes	0.9939	0.9963	99.800%
719	644.089	644.402	1535.814	1539.635	2625.559	2628.605	0.9995	0.9975	0.9988	62.81	289.8	68.41	1.03	Yes	$f_{1A}/f_{1P}>f_{3A}/f_{3P}>f_{2A}/f_{2P}$	3	Yes	0.9982	0.9978	99.965%
720	644.584	645.207	1534.668	1542.249	2622.575	2628.219	0.9990	0.9951	0.9979	82.1	288.69	78.72	1.52	Yes	$f_{1A}/f_{1P}>f_{3A}/f_{3P}>f_{2A}/f_{2P}$	3	Yes	0.9926	0.9951	99.793%
721	643.621	648.556	1534.502	1536.747	2619.982	2624.303	0.9924	0.9985	0.9984	73.53	372.59	115.34	2.04	Yes	$f_{2A}/f_{2P}>f_{3A}/f_{3P}>f_{1A}/f_{1P}$	5	Yes	0.9927	0.9914	99.890%
722	643.131	645.279	1535.510	1537.134	2623.344	2631.986	0.9967	0.9989	0.9967	43.04	423.27	46.48	2.12	Yes	$f_{2A}/f_{2P}>f_{3A}/f_{3P}>f_{1A}/f_{1P}$	5	Yes	0.9968	0.9979	99.909%
723	644.065	644.170	1536.186	1539.461	2626.791	2628.702	0.9998	0.9979	0.9993	23.35	276.05	112.62	1.37	Yes	$f_{1A}/f_{1P}>f_{3A}/f_{3P}>f_{2A}/f_{2P}$	3	Yes	1.0020	0.9982	-
724	643.583	644.487	1536.321	1537.064	2626.558	2630.423	0.9986	0.9995	0.9985	38.92	433.95	59.8	0.91	Yes	$f_{2A}/f_{2P}>f_{1A}/f_{1P}>f_{3A}/f_{3P}$	2	No	-	0.9989	-
725	643.448	643.820	1537.337	1537.891	2628.641	2631.961	0.9994	0.9996	0.9987	26.25	179.68	107.45	1.22	Yes	$f_{2A}/f_{2P}>f_{1A}/f_{1P}>f_{3A}/f_{3P}$	2	Yes	1.0002	0.9983	-
726	643.774	643.963	1536.803	1538.413	2628.407	2629.937	0.9997	0.9990	0.9994	50.15	224.27	57.16	0.77	Yes	$f_{1A}/f_{1P}>f_{3A}/f_{3P}>f_{2A}/f_{2P}$	3	Yes	1.0004	0.9989	-
727	643.098	643.410	1536.786	1538.221	2629.222	2633.474	0.9995	0.9991	0.9984	58.18	146.84	63.94	1.55	Yes	$f_{1A}/f_{1P}>f_{2A}/f_{2P}>f_{3A}/f_{3P}$	2	Yes	1.0006	0.9971	-
728	643.704	645.780	1535.448	1538.204	2622.634	2626.126	0.9968	0.9982	0.9987	31.13	351.51	96.2	2.48	Yes	$f_{3A}/f_{3P}>f_{2A}/f_{2P}>f_{1A}/f_{1P}$	4	Yes	0.9967	0.9963	99.969%
729	643.755	644.164	1536.858	1539.948	2628.733	2632.152	0.9994	0.9980	0.9987	43.25	215.69	128.91	1	Yes	$f_{1A}/f_{1P}>f_{3A}/f_{3P}>f_{2A}/f_{2P}$	3	Yes	0.9965	0.9972	99.942%
730	640.969	641.404	1530.198	1537.766	2626.510	2630.366	0.9993	0.9951	0.9985	37.02	117.66	127.24	2.6	Yes	$f_{1A}/f_{1P}>f_{3A}/f_{3P}>f_{2A}/f_{2P}$	3	Yes	0.9960	0.9939	99.834%
731	642.324	642.393	1534.090	1538.414	2630.557	2630.765	0.9999	0.9972	0.9999	42.37	126.88	106.09	1.97	Yes	$f_{3A}/f_{3P}>f_{1A}/f_{1P}>f_{2A}/f_{2P}$	1	Yes	0.6449	0.9956	70.921%
732	642.521	646.517	1534.194	1536.578	2618.587	2633.242	0.9938	0.9984	0.9944	67.33	415.22	54.37	2.36	Yes	$f_{2A}/f_{2P}>f_{3A}/f_{3P}>f_{1A}/f_{1P}$	5	Yes	0.9941	0.9957	99.866%
733	644.525	648.765	1532.954	1540.934	2615.901	2621.038	0.9935	0.9948	0.9980	82.32	341.55	131.3	2.04	Yes	$f_{3A}/f_{3P}>f_{2A}/f_{2P}>f_{1A}/f_{1P}$	4	Yes	0.9922	0.9890	99.738%
734	642.846	647.521	1533.490	1536.568	2618.293	2635.724	0.9928	0.9980	0.9934	72.48	421.56	79.48	2.04	Yes	$f_{2A}/f_{2P}>f_{3A}/f_{3P}>f_{1A}/f_{1P}$	5	Yes	0.9931	0.9942	99.912%
735	643.961	644.521	1536.002	1539.015	2624.608	2628.713	0.9991	0.9980	0.9984	22.66	313.97	84.08	1.95	Yes	$f_{1A}/f_{1P}>f_{3A}/f_{3P}>f_{2A}/f_{2P}$	3	Yes	0.9937	0.9982	99.631%
736	643.470	644.355	1537.491	1540.843	2628.343	2638.524	0.9986	0.9978	0.9961	86.42	193.23	73.61	1.64	Yes	$f_{1A}/f_{1P}>f_{2A}/f_{2P}>f_{3A}/f_{3P}$	2	Yes	0.9964	0.9948	99.867%
737	642.935	645.546	1535.365	1536.797	2622.405	2631.161	0.9960	0.9991	0.9967	41.98	408.44	49.66	2.48	Yes	$f_{2A}/f_{2P}>f_{3A}/f_{3P}>f_{1A}/f_{1P}$	5	Yes	0.9962	0.9974	99.897%
738	642.336	642.836	1534.896	1537.252	2627.941	2629.944	0.9992	0.9985	0.9992	73.15	104.75	81.32	0.88	Yes	$f_{3A}/f_{3P}>f_{1A}/f_{1P}>f_{2A}/f_{2P}$	1	Yes	0.6439	0.9974	70.691%

739	644.134	645.679	1534.805	1540.671	2620.799	2627.605	0.9976	0.9962	0.9974	38.26	322.25	112.4	2.05	Yes	$f_{1A}/f_{1P}>f_{3A}/f_{3P}>f_{2A}/f_{2P}$	3	Yes	0.9760	0.9956	98.373%
740	642.803	643.990	1537.386	1538.664	2625.649	2638.540	0.9982	0.9992	0.9951	58.71	175.35	63.88	2.55	Yes	$f_{2A}/f_{2P}>f_{1A}/f_{1P}>f_{3A}/f_{3P}$	2	Yes	0.9942	0.9953	99.911%
741	644.171	645.942	1534.494	1540.646	2620.360	2627.102	0.9973	0.9960	0.9974	67.78	325.44	87.84	1.69	Yes	$f_{3A}/f_{3P}>f_{1A}/f_{1P}>f_{2A}/f_{2P}$	1	Yes	0.6411	0.9950	70.659%
742	643.680	643.967	1536.895	1538.464	2628.265	2631.012	0.9996	0.9990	0.9990	31.12	207.98	72.44	1.17	Yes	$f_{1A}/f_{1P}>f_{2A}/f_{2P}>f_{3A}/f_{3P}$	2	Yes	1.0008	0.9987	-
743	643.577	645.372	1536.406	1536.833	2625.710	2628.008	0.9972	0.9997	0.9991	47.46	378.68	78.29	1.42	Yes	$f_{2A}/f_{2P}>f_{3A}/f_{3P}>f_{1A}/f_{1P}$	5	Yes	0.9974	0.9974	100.000%
744	643.416	644.630	1535.766	1542.048	2626.978	2636.213	0.9981	0.9959	0.9965	42.61	212.79	88.29	2.59	Yes	$f_{1A}/f_{1P}>f_{3A}/f_{3P}>f_{2A}/f_{2P}$	3	Yes	0.9819	0.9952	98.898%
745	644.423	647.136	1534.198	1541.003	2619.167	2624.911	0.9958	0.9956	0.9978	75.57	334.12	118.9	1.67	Yes	$f_{3A}/f_{3P}>f_{1A}/f_{1P}>f_{2A}/f_{2P}$	1	Yes	0.6391	0.9925	70.693%
746	644.687	644.777	1534.602	1543.685	2624.883	2628.666	0.9999	0.9941	0.9986	38.78	268.7	122.3	2.16	Yes	$f_{1A}/f_{1P}>f_{3A}/f_{3P}>f_{2A}/f_{2P}$	3	Yes	1.0022	0.9949	-
747	642.592	642.640	1533.678	1538.047	2628.172	2630.260	0.9999	0.9972	0.9992	23.83	119.78	96.18	2.55	Yes	$f_{1A}/f_{1P}>f_{3A}/f_{3P}>f_{2A}/f_{2P}$	3	Yes	1.0030	0.9971	-
748	642.944	643.578	1538.299	1538.553	2629.870	2636.697	0.9990	0.9998	0.9974	67.63	166.61	117.22	1.29	Yes	$f_{2A}/f_{2P}>f_{1A}/f_{1P}>f_{3A}/f_{3P}$	2	Yes	0.9983	0.9949	99.717%
749	644.491	645.332	1530.927	1543.970	2617.920	2627.334	0.9987	0.9916	0.9964	80.09	284.48	63.23	2.59	Yes	$f_{1A}/f_{1P}>f_{3A}/f_{3P}>f_{2A}/f_{2P}$	3	Yes	0.9887	0.9935	99.600%
750	643.857	644.043	1536.728	1538.946	2628.458	2630.060	0.9997	0.9986	0.9994	70.12	230.54	59.11	0.76	Yes	$f_{1A}/f_{1P}>f_{3A}/f_{3P}>f_{2A}/f_{2P}$	3	Yes	1.0005	0.9984	-
751	642.370	643.129	1535.702	1538.525	2629.670	2634.690	0.9988	0.9982	0.9981	36.23	150.13	109.97	2.42	Yes	$f_{1A}/f_{1P}>f_{2A}/f_{2P}>f_{3A}/f_{3P}$	2	Yes	0.9973	0.9952	99.824%
752	642.746	643.015	1536.594	1538.187	2630.076	2633.727	0.9996	0.9990	0.9986	49.24	147.79	130.68	1.37	Yes	$f_{1A}/f_{1P}>f_{2A}/f_{2P}>f_{3A}/f_{3P}$	2	Yes	1.0010	0.9956	-
753	641.132	641.903	1532.035	1537.916	2627.774	2630.841	0.9988	0.9962	0.9988	51.9	113.93	111.13	1.93	Yes	$f_{3A}/f_{3P}>f_{1A}/f_{1P}>f_{2A}/f_{2P}$	1	Yes	0.6433	0.9945	70.882%
754	643.620	644.709	1536.740	1536.767	2626.767	2628.772	0.9983	1.0000	0.9992	37.67	386.12	74.25	1.08	Yes	$f_{2A}/f_{2P}>f_{3A}/f_{3P}>f_{1A}/f_{1P}$	5	Yes	0.9984	0.9985	99.993%
755	643.924	644.786	1536.105	1538.370	2625.312	2627.790	0.9987	0.9985	0.9991	69.06	335.61	73.48	0.9	Yes	$f_{3A}/f_{3P}>f_{1A}/f_{1P}>f_{2A}/f_{2P}$	1	No	-	0.9977	-
756	643.299	647.428	1534.027	1536.886	2620.876	2636.096	0.9936	0.9981	0.9942	57.42	432.72	114.34	1.63	Yes	$f_{2A}/f_{2P}>f_{3A}/f_{3P}>f_{1A}/f_{1P}$	5	Yes	0.9939	0.9947	99.937%
757	640.896	641.569	1532.993	1537.625	2629.518	2630.828	0.9990	0.9970	0.9995	89.45	117.86	108.93	1.42	Yes	$f_{3A}/f_{3P}>f_{1A}/f_{1P}>f_{2A}/f_{2P}$	1	Yes	0.6435	0.9931	71.013%
758	645.179	645.248	1532.480	1547.359	2624.434	2627.746	0.9999	0.9904	0.9987	63.48	262.36	111.68	2.43	Yes	$f_{1A}/f_{1P}>f_{3A}/f_{3P}>f_{2A}/f_{2P}$	3	Yes	1.0026	0.9915	-
759	643.608	645.620	1536.094	1536.684	2624.806	2627.475	0.9969	0.9996	0.9990	78.66	377.87	63.83	1.37	Yes	$f_{2A}/f_{2P}>f_{3A}/f_{3P}>f_{1A}/f_{1P}$	5	Yes	0.9970	0.9966	99.960%
760	643.301	644.167	1537.519	1539.978	2629.100	2635.947	0.9987	0.9984	0.9974	36.19	190.92	129.31	1.83	Yes	$f_{1A}/f_{1P}>f_{2A}/f_{2P}>f_{3A}/f_{3P}$	2	Yes	0.9966	0.9957	99.928%
761	643.389	648.768	1533.155	1537.808	2618.338	2619.256	0.9917	0.9970	0.9996	62.37	360.54	116.68	2.58	Yes	$f_{3A}/f_{3P}>f_{2A}/f_{2P}>f_{1A}/f_{1P}$	4	Yes	0.9899	0.9907	99.932%
762	645.005	646.124	1532.514	1545.744	2618.132	2627.320	0.9983	0.9914	0.9965	73.26	288.4	107.91	2.13	Yes	$f_{1A}/f_{1P}>f_{3A}/f_{3P}>f_{2A}/f_{2P}$	3	Yes	0.9837	0.9916	99.341%
763	641.619	642.648	1533.300	1537.288	2625.136	2630.452	0.9984	0.9974	0.9980	53.98	94.45	59.96	1.81	Yes	$f_{1A}/f_{1P}>f_{3A}/f_{3P}>f_{2A}/f_{2P}$	3	Yes	0.9852	0.9971	99.017%
764	642.440	642.822	1535.258	1538.619	2628.625	2633.903	0.9994	0.9978	0.9980	63.61	140.47	60.78	2.3	Yes	$f_{1A}/f_{1P}>f_{3A}/f_{3P}>f_{2A}/f_{2P}$	3	Yes	0.9969	0.9956	99.889%
765	643.661	644.707	1536.735	1536.759	2626.878	2628.847	0.9984	1.0000	0.9993	32.47	388.03	107.2	0.89	Yes	$f_{2A}/f_{2P}>f_{3A}/f_{3P}>f_{1A}/f_{1P}$	5	Yes	0.9985	0.9984	99.995%
766	643.908	644.017	1536.399	1539.190	2628.502	2628.681	0.9998	0.9982	0.9999	25.24	248.91	114.54	1.13	Yes	$f_{3A}/f_{3P}>f_{1A}/f_{1P}>f_{2A}/f_{2P}$	1	Yes	0.6448	0.9984	70.683%
767	642.084	643.904	1537.942	1541.728	2627.907	2635.640	0.9972	0.9975	0.9971	78.02	181.08	122.35	2.43	Yes	$f_{2A}/f_{2P}>f_{1A}/f_{1P}>f_{3A}/f_{3P}$	2	Yes	0.9895	0.9885	99.919%
768	644.164	645.016	1534.801	1545.012	2628.055	2632.964	0.9987	0.9934	0.9981	54.98	235.61	110.26	2.19	Yes	$f_{1A}/f_{1P}>f_{3A}/f_{3P}>f_{2A}/f_{2P}$	3	Yes	0.9885	0.9934	99.592%
769	636.017	639.273	1523.065	1533.655	2615.678	2628.804	0.9949	0.9931	0.9950	84.94	90.44	102.97	2.3	Yes	$f_{3A}/f_{3P}>f_{1A}/f_{1P}>f_{2A}/f_{2P}$	1	Yes	0.6378	0.9900	70.793%

770	643.366	646.333	1534.791	1536.882	2623.278	2632.308	0.9954	0.9986	0.9966	28.19	414.37	111.99	2.3	Yes	$f_{2A}/f_{2P}>f_{3A}/f_{3P}>f_{1A}/f_{1P}$	5	Yes	0.9956	0.9964	99.933%
771	641.037	642.137	1532.568	1537.004	2626.262	2630.363	0.9983	0.9971	0.9984	58.12	104.04	117.68	1.38	Yes	$f_{3A}/f_{3P}>f_{1A}/f_{1P}>f_{2A}/f_{2P}$	1	Yes	0.6426	0.9953	70.756%
772	642.523	647.413	1533.955	1535.962	2617.504	2632.076	0.9924	0.9987	0.9945	68.86	403.34	70.51	2.42	Yes	$f_{2A}/f_{2P}>f_{3A}/f_{3P}>f_{1A}/f_{1P}$	5	Yes	0.9928	0.9942	99.885%
773	643.250	643.564	1537.819	1538.014	2629.893	2633.953	0.9995	0.9999	0.9985	85.9	160.37	91.21	0.79	Yes	$f_{2A}/f_{2P}>f_{1A}/f_{1P}>f_{3A}/f_{3P}$	2	Yes	1.0006	0.9969	-
774	644.086	644.523	1535.394	1540.040	2624.324	2628.959	0.9993	0.9970	0.9982	35.02	294.86	70.19	1.88	Yes	$f_{1A}/f_{1P}>f_{3A}/f_{3P}>f_{2A}/f_{2P}$	3	Yes	0.9960	0.9977	99.859%
775	643.896	644.423	1536.086	1538.641	2625.623	2628.747	0.9992	0.9983	0.9988	37.09	316.8	56.27	1.44	Yes	$f_{1A}/f_{1P}>f_{3A}/f_{3P}>f_{2A}/f_{2P}$	3	Yes	0.9943	0.9985	99.652%
776	644.621	644.944	1535.119	1542.759	2625.251	2628.423	0.9995	0.9950	0.9988	68.73	274.9	115.6	1.26	Yes	$f_{1A}/f_{1P}>f_{3A}/f_{3P}>f_{2A}/f_{2P}$	3	Yes	0.9980	0.9950	99.750%
777	643.426	644.392	1536.752	1540.599	2627.134	2637.117	0.9985	0.9975	0.9962	58.51	197.8	71.83	2	Yes	$f_{1A}/f_{1P}>f_{2A}/f_{2P}>f_{3A}/f_{3P}$	2	Yes	0.9958	0.9958	99.998%
778	644.319	644.601	1535.315	1541.169	2625.146	2628.915	0.9996	0.9962	0.9986	44.81	280.22	88.22	1.55	Yes	$f_{1A}/f_{1P}>f_{3A}/f_{3P}>f_{2A}/f_{2P}$	3	Yes	0.9988	0.9969	99.846%
779	641.355	641.893	1531.791	1538.364	2627.778	2630.496	0.9992	0.9957	0.9990	54.47	115.55	72.45	2.48	Yes	$f_{1A}/f_{1P}>f_{3A}/f_{3P}>f_{2A}/f_{2P}$	3	Yes	0.9941	0.9952	99.911%
780	644.299	644.308	1536.376	1540.394	2627.899	2628.623	1.0000	0.9974	0.9997	80.22	261.93	101.18	0.72	Yes	$f_{1A}/f_{1P}>f_{3A}/f_{3P}>f_{2A}/f_{2P}$	3	Yes	1.0037	0.9971	-
781	642.783	651.301	1530.547	1534.587	2612.124	2638.096	0.9869	0.9974	0.9902	85.47	415.65	123.85	2.4	Yes	$f_{2A}/f_{2P}>f_{3A}/f_{3P}>f_{1A}/f_{1P}$	5	Yes	0.9875	0.9874	99.994%
782	643.914	645.233	1535.996	1538.538	2624.157	2627.500	0.9980	0.9983	0.9987	24.06	342.5	115.4	2.04	Yes	$f_{3A}/f_{3P}>f_{2A}/f_{2P}>f_{1A}/f_{1P}$	4	Yes	0.9982	0.9972	99.916%
783	643.596	646.533	1535.834	1537.043	2624.261	2626.293	0.9955	0.9992	0.9992	40.48	372.75	113.22	1.99	Yes	$f_{3A}/f_{3P}>f_{2A}/f_{2P}>f_{1A}/f_{1P}$	4	Yes	0.9949	0.9955	99.953%
784	644.343	648.715	1533.474	1539.927	2618.324	2621.143	0.9933	0.9958	0.9989	79.02	348.85	132.91	1.96	Yes	$f_{3A}/f_{3P}>f_{2A}/f_{2P}>f_{1A}/f_{1P}$	4	Yes	0.9919	0.9898	99.823%
785	643.817	644.671	1536.318	1537.974	2626.422	2627.750	0.9987	0.9989	0.9995	29.4	343.56	124.96	1.01	Yes	$f_{3A}/f_{3P}>f_{2A}/f_{2P}>f_{1A}/f_{1P}$	4	Yes	0.9992	0.9981	99.912%
786	643.009	643.136	1536.559	1538.030	2629.886	2632.572	0.9998	0.9990	0.9990	73.23	140.22	83.69	1	Yes	$f_{1A}/f_{1P}>f_{2A}/f_{2P}>f_{3A}/f_{3P}$	2	Yes	1.0020	0.9969	-
787	642.456	650.297	1530.289	1535.466	2612.183	2640.820	0.9879	0.9966	0.9892	85.09	428.04	105.33	2.41	Yes	$f_{2A}/f_{2P}>f_{3A}/f_{3P}>f_{1A}/f_{1P}$	5	Yes	0.9885	0.9893	99.933%
788	643.369	643.531	1537.096	1537.761	2629.397	2631.683	0.9997	0.9996	0.9991	71.44	147.51	69.16	0.74	Yes	$f_{1A}/f_{1P}>f_{2A}/f_{2P}>f_{3A}/f_{3P}$	2	Yes	1.0017	0.9982	-
789	643.573	646.258	1535.307	1537.847	2623.614	2624.836	0.9958	0.9983	0.9995	44.09	359.8	92.15	2.17	Yes	$f_{3A}/f_{3P}>f_{2A}/f_{2P}>f_{1A}/f_{1P}$	4	Yes	0.9954	0.9956	99.981%
790	644.564	644.791	1534.538	1543.037	2624.087	2628.732	0.9996	0.9945	0.9982	40.26	274.27	108.19	2.09	Yes	$f_{1A}/f_{1P}>f_{3A}/f_{3P}>f_{2A}/f_{2P}$	3	Yes	0.9997	0.9955	99.650%
791	643.970	645.987	1536.172	1536.519	2625.107	2630.344	0.9969	0.9998	0.9980	85.28	401.27	134.53	0.69	Yes	$f_{2A}/f_{2P}>f_{3A}/f_{3P}>f_{1A}/f_{1P}$	5	Yes	0.9970	0.9960	99.918%
792	644.181	644.758	1535.471	1540.484	2623.389	2628.762	0.9991	0.9967	0.9980	27.68	299.66	105.37	2	Yes	$f_{1A}/f_{1P}>f_{3A}/f_{3P}>f_{2A}/f_{2P}$	3	Yes	0.9934	0.9971	99.693%
793	643.650	645.929	1536.217	1537.096	2625.326	2627.266	0.9965	0.9994	0.9993	41.53	374.13	102.44	1.65	Yes	$f_{2A}/f_{2P}>f_{3A}/f_{3P}>f_{1A}/f_{1P}$	5	Yes	0.9966	0.9965	99.988%
794	642.004	648.086	1531.767	1536.512	2613.658	2638.393	0.9906	0.9969	0.9906	74.94	432.96	71.24	2.56	Yes	$f_{2A}/f_{2P}>f_{3A}/f_{3P}>f_{1A}/f_{1P}$	5	Yes	0.9910	0.9932	99.814%
795	643.916	644.840	1535.643	1539.110	2624.132	2628.425	0.9986	0.9977	0.9984	43.01	324.43	68.21	1.6	Yes	$f_{1A}/f_{1P}>f_{3A}/f_{3P}>f_{2A}/f_{2P}$	3	Yes	0.9872	0.9977	99.133%
796	644.106	644.859	1535.428	1540.143	2623.101	2628.689	0.9988	0.9969	0.9979	30.78	308.54	95.45	1.94	Yes	$f_{1A}/f_{1P}>f_{3A}/f_{3P}>f_{2A}/f_{2P}$	3	Yes	0.9903	0.9972	99.431%
797	643.774	645.225	1535.883	1538.265	2624.813	2627.264	0.9978	0.9985	0.9991	38.88	348.3	82.15	1.69	Yes	$f_{3A}/f_{3P}>f_{2A}/f_{2P}>f_{1A}/f_{1P}$	4	Yes	0.9979	0.9973	99.951%
798	643.572	645.289	1536.169	1536.806	2625.483	2630.336	0.9973	0.9996	0.9982	43.81	402.89	95.97	1.11	Yes	$f_{2A}/f_{2P}>f_{3A}/f_{3P}>f_{1A}/f_{1P}$	5	Yes	0.9975	0.9977	99.987%
799	644.806	646.754	1533.898	1543.055	2618.081	2626.085	0.9970	0.9941	0.9970	81.73	313.29	116.57	1.67	Yes	$f_{1A}/f_{1P}>f_{3A}/f_{3P}>f_{2A}/f_{2P}$	3	Yes	0.9688	0.9921	98.071%
800	643.529	643.835	1537.535	1538.075	2628.874	2632.646	0.9995	0.9996	0.9986	70.71	176.31	68.45	0.78	Yes	$f_{2A}/f_{2P}>f_{1A}/f_{1P}>f_{3A}/f_{3P}$	2	Yes	1.0007	0.9981	-
